# Occupational Noise and Hypertension Risk: A Systematic Review and Meta-Analysis

**DOI:** 10.3390/ijerph17176281

**Published:** 2020-08-28

**Authors:** Ulrich Bolm-Audorff, Janice Hegewald, Anna Pretzsch, Alice Freiberg, Albert Nienhaus, Andreas Seidler

**Affiliations:** 1 Division of Occupational Health, Department of Occupational Safety and Environment, Regional Government of South Hesse, 65197 Wiesbaden, Germany; ulrich.bolm-audorff@rpda.hessen.de; 2 Associate Professor of Occupational Medicine, Justus-Liebig-University, 35392 Giessen, Germany; 3 Institute and Policlinic of Occupational and Social Medicine (IPAS), Faculty of Medicine, Technische Universität Dresden, 01307 Dresden, Germany; anna.pretzsch@mailbox.tu-dresden.de (A.P.); alice.freiberg@tu-dresden.de (A.F.); ArbSozPH@mailbox.tu-dresden.de (A.S.); 4 Institute of Sociology, Faculty of Behavioral and Social Sciences, Chemnitz University of Technology, Thüringer Weg 9, 09126 Chemnitz, Germany; 5 Competence Center for Epidemiology and Health Services Research for Healthcare Professionals (CVcare), Institute for Health Services Research in Dermatology and Nursing (IVDP), University Medical Centre Hamburg-Eppendorf, 20246 Hamburg, Germany; a.nienhaus@uke.de; 6 Department of Occupational Medicine, Hazardous Substances and Public Health, Institution for Statutory Accident Insurance and Prevention in the Health and Welfare Services (BGW), 22089 Hamburg, Germany

**Keywords:** noise, work, occupation, arterial hypertension, systematic review, meta-analysis, dose response relationship, blood pressure

## Abstract

A number of epidemiological studies report an association between occupational noise exposure and arterial hypertension. Existing systematic reviews report conflicting results, so we conducted an updated systematic review with meta-analysis. We registered the review protocol with PROSPERO (registration no.: CRD 42019147923) and searched for observational epidemiological studies in literature databases (Medline, Embase, Scopus, Web of Science). Two independent reviewers screened the titles/abstracts and full texts of the studies. Two reviewers also did the quality assessment and data extraction. Studies without adequate information on recruitment, response, or without a comparison group that was exposed to occupational noise under 80 dB(A) were excluded. The literature search yielded 4583 studies, and 58 studies were found through hand searching. Twenty-four studies were included in the review. The meta-analysis found a pooled effect size (ES) for hypertension (systolic/diastolic blood pressure ≥140/90 mmHg) due to noise exposures ≥80 dB(A) of 1.75 (95% CI 1.46–2.10). There is no substantial risk difference between men and women, but data concerning this question are limited. We found a positive dose-response-relationship: ES = 1.21 (95% CI 0.78–1.87) ≤ 80 dB(A), ES = 1.66 (95% CI 1.24–2.22) > 80–≤85 dB(A), and ES = 3.38 (95% CI 1.37–8.35) > 85–≤90 dB(A). We found high quality of evidence that occupational noise exposure increases the risk of hypertension.

## 1. Introduction

It has long been established that exposure to occupational noise increases the risk of developing sensorineural hearing loss. Noise-induced hearing loss is one of the most commonly recognized occupational diseases in most industrialized countries [1]. In occupational and environmental medicine, possible extra-aural noise effects on the cardiovascular system, including arterial hypertension, have been discussed for decades [2,3,4,5].

Various animal studies report correlations between acute [6,7,8,9] or chronic noise exposures [10,11,12,13,14,15,16,17,18,19,20] and an increase of blood pressure. On the other hand, four experimental studies found no association between noise and blood pressure increase [21,22,23,24]. One experimental study using two different strains of rats found a strong response to acute noise exposure could be demonstrated in one strain, but not in another rat strain [14]. To the best of our knowledge, a systematic review of animal studies on this topic does not exist. However, in our opinion, the results of these experimental studies support biological plausibility, since most of the studies report an association between noise exposure and blood pressure increase. There is also some experimental evidence that noise exposure and genetic factors might interact to increase blood pressure [6,14]. The mechanisms for this possible interaction have not yet been further investigated.

Concerning the mechanism for the association between noise and hypertension, two hypothalamus-mediated mechanisms that could explain this reaction are discussed in the literature [25]. On the one hand, the activation of the autonomic nervous system (sympathetic nervous system) causes an increased release of catecholamines, which e.g., increase the heart rate and lead to vascular constriction and, thus, to an increase in blood pressure. On the other hand, an increased release of corticotropin by the endocrine system leads to an increased cortisol level, which increases the effect of catecholamines and, likewise, increases blood pressure. Narrative reviews indicate increased secretion of blood pressure-increasing hormones, such as epinephrine, norepinephrine, and cortisol, in noise-exposed laboratory animals and workers [26,27].

Numerous epidemiological studies have considered the association between occupational noise exposure and arterial hypertension. In the last decade, four systematic reviews with varying results were published. Pooled risk estimates for the development of arterial hypertension of occupational noise exposed employees were reported to be 1.08 (95% CI 1.05–1.11) [28], 1.37 (95% CI 1.01–1.87) [29], 2.55 (95% CI 1.94–3.36) [30], and 2.56 (95% CI 2.01–3.27) [31]. None of the studies examined whether workplace noise exposure has a different effect on the risk of hypertension for women and men. Furthermore, the following methodological shortcomings may diminish the quality of this summarized evidence [31,32,33]: no publication of a study protocol prior to conducting the review [28,29,30,31]; no doubled screening of studies [28,29,30,31]; no doubled data extraction [29,30,31]; no assessment of study quality [29,30,31]; no consideration of study quality in the meta-analysis [28,29,30,31]; no consideration of the funding agencies [29,31]; no definition of hypertension [28]; no estimate of publication bias [29,31]; limited publication languages included [28,29,30]; limited to studies conducted in certain countries [28,30]; no inclusion of unpublished reports [29,31]; or, no information on conflicts of interest [33]. Therefore, we decided to conduct an updated systematic review on this topic, and to use methods currently recommended to ensure the quality of summarized evidence [34].

### Aims and Objectives

We conducted a systematic review in order to determine whether employees chronically exposed to noise at work had an elevated risk for developing arterial hypertension. We aimed to examine the body of evidence regarding occupational noise exposure, and sought all observational epidemiological studies considering the incidence or prevalence of hypertension among workers that were chronically exposed to noise when compared to workers with average daily noise exposure levels (LEX,8h) ≤ 80 A-weighted decibels (dB[A]). In addition to examining the strength of the published evidence, we set out to examine whether risks differ between men and women, and to determine the dose-response relationship between occupational noise and hypertension using meta-analyses.

## 2. Materials and Methods

We searched the entirety of the Medline (Ovid), Embase (Ovid), Scopus, and Web of Science literature databases on May 19, 2019, to find all observational epidemiological research on the effects of noise and risk of hypertension. The search strings comprised keywords for hypertension and occupational noise combined with Boolean operators, and they were adapted to each database accordingly. For example, the Web of Science search string was: (TS = (hypertension OR hypertens* OR “blood pressure” OR bloodpressure)) AND (TS = (noise)). The Medline search string is included in the online supplement (Table S1). A protocol of the systematic review was registered a priori with the PROSPERO database of systematic reviews (PROSPERO ID: CRD42019147923).

The search was conducted without any language restrictions, but we only considered studies with abstracts available in English, French, German, Italian, or Spanish. We supplemented the electronic search by searching the reference lists of included studies and the reference lists of key articles to identify additional relevant literature. We also used the Web of Sciences’s citation tracking tool to find additional research articles citing key articles. We did not explicitly search for grey literature, but studies published in non-peer reviewed journals or elsewhere and found through reference list searching would have been included.

### 2.1. Eligibility Criteria

The scope and objectives of the review were specified according to the population, exposure, comparison, and outcome of interest, as shown in Table 1.

Because of changes in definitions of arterial hypertension over time, we used a broad definition of hypertension as a systolic (SBP) and diastolic blood pressure (DBP) ≥ 130/80 mmHg to include studies applying different definitions of hypertension [35,36,37].

We included only observational epidemiological studies, such as cohort, case-control, case-cohort, nested-case-control, and cross-sectional studies, in order to ensure an adequate evidence-level. Due to the long-term nature of the occupational noise exposure, experimental studies cannot sufficiently assess the effects of noisy working environments on hypertension risk. We also excluded research using study designs that provide inadequate evidence of associations, such as ecological studies and case reports. Letters to the editor, congress abstracts, and reviews were also excluded.

### 2.2. Study Selection

We collected the search results in an Endnote reference library and removed duplicate listings prior to selecting studies. Two reviewers independently screened the titles and abstracts of the results, and conflicts were collectively resolved. We piloted the title/abstract screening using the first 200 citations. Afterwards, two reviewers independently assessed the full-texts of the publications for eligibility. Diverging assessments were resolved with a third reviewer, and all reasons for excluding each study recorded. When possible, multilingual colleagues (mentioned in the acknowledgements) helped to assess and translate the studies published in languages other than English, French, or German. In some cases (e.g., Korean), publications were professionally translated. In total, we screened the full texts of 180 articles (including 34 non-English publications: eight French, eight Russian, five Spanish, four Italian, three Chinese, two Croatian, and two Korean studies, as well as one Portuguese and one Polish studies).

In order to help ensure the internal validity of included studies, we included only studies reporting a response of at least 10% or studies based on compulsory occupational preventive examinations, such as for the early detection of deafness or aviation medical examinations of pilots exposed to noise, where we can assume that most employees were included.

### 2.3. Data Extraction and Risk of Bias

Two reviewers extracted data from each eligible study including the first author, year of publication, and information regarding the study design, location(s), years conducted, occupational groups considered, population characteristics (i.e., number of participants, sex, and age), noise assessment methods, noise exposure levels, duration of noise exposure measurement, any use of hearing protective devices reported, and hypertension definitions. The study results extracted included the prevalence or incidence of hypertension reported for each group and any measures of association with its corresponding 95% confidence interval (CI), such as relative risks (RR), hazard ratios (HR), prevalence ratios (PR), and odds ratios (OR). We also noted any adjustment factors used to obtain adjusted risk estimates. We attempted to contact authors for additional information if information was missing or unclear.

The risk of bias was assessed by two reviewers using a schema used previously for other occupational reviews [38,39] and adapted for the current research question (Supplementary Table S7). We considered study characteristics pertaining to study recruitment and follow-up, exposure measurements, outcome assessment, consideration of confounding and effect modification, analysis methods, and chronology to be domains of major importance regarding bias. If we judged at least one of these domains to have a high risk of bias, the study received an overall high risk of bias rating. We considered the blinding of assessors (or lack thereof), sources of funding, and any conflicts of interest to be “minor domains”. Differing risk of bias assessments were discussed until consensus was reached. We pilot-tested the data extraction and risk of bias forms on two of the included studies.

### 2.4. Data Synthesis

Meta-analysis of the study results was conducted in order to determine the overall noise-related risk of developing an arterial hypertension. When available, the fully-adjusted relative risk estimates of the individual studies were used for the meta-analysis, unless this risk estimate adjusted for a component of exposure, such as occupation or length of employment, which would result in over-adjustment. If the results were reported for more than one exposure category, we included the loudest exposure category in the meta-analysis unless the highest exposure category was too sparse (fewer than 10 cases). If results were reported separately for subgroups, such as men and women, we included all of the subgroups results in the meta-analysis when we were certain the populations did not overlap. If studies did not report any risk estimate or if risk was reported for a continuous exposure (per 1 dB increase in noise), we calculated the prevalence ratios from the published frequencies. When the frequencies were stratified for potential confounding factors, such as age-groups, we calculated an adjusted risk for these factors while using a Poisson regression model weighted by the number of workers in each category. If we needed to calculate prevalence ratios for multiple exposed subgroups using only one comparison group, we divided the people in the comparison group by the number of exposure groups to prevent artificially counting the people in the comparison group multiple times and thereby inadvertently increasing the power of the study.

Hypertension is common in the general population, so the rare disease assumption does not apply and ORs will overestimate the relative risks for hypertension. Therefore, we also converted the adjusted OR to RR/PR using the formula that was proposed by Zhang and Yu [40]. The corrected PRs were included in the meta-analysis.

The random-effects meta-analyses were conducted in Stata version 14 [41] using the *metan* package [42]. Subgroup-analyses examined results based on similar hypertension definitions and noise exposure levels. First, different definitions of hypertension (e.g., 140/90 mmHg, 160/95 mmHg, physician diagnosed hypertension) were considered separately. We also conducted subgroup analyses for noise exposures in the following ranges if enough studies were available: >80–≤85 dB(A), >85–≤90 dB(A), >90–≤95 dB(A), >95–≤100 dB(A), >100 dB(A), and >90 dB(A) versus <80 dB(A). A further subgroup analysis was done in order to consider noise levels under ≤80 dB(A) among studies reporting risk estimates for lower noise exposure levels. We also considered studies with high and low risk of bias separately if two or more studies had an overall low risk of bias in order to assess the impact of bias. Subgroup analyses were also conducted for sex and study design.

Heterogeneity was assessed with the I^2^ statistic and a Chi-squared (χ^2^) test for heterogeneity. We examined potential publication bias using funnel plots and the Egger’s test if five or more studies could be included in a meta-analysis.

### 2.5. Sensitivity Analysis

We conducted a “leave-one-out” analysis to determine the importance of individual studies on the overall meta-analysis results. In addition, we used the average noise exposure levels and information on years of exposure reported (if available) to determine an A-weighted noise exposure level normalized to a 40-year working life in order to estimate a cumulative effect of occupational noise exposure on the hypertension risk. If noise exposure was reported as a range, the mean of the range boundaries was used for the calculations. Noise exposure levels normalized to a 40-year working life (L_EX,40y_) were calculated by adapting the formula for calculating L_EX,8h_ described in ISO 1999 [43] to a reference time of 40 years instead of 8 h. We also assumed the average noise exposures reported by studies were constant and corresponded to the L_EX,8h_ experienced every workday for the reported duration of work (in years) during full-time work (40 h per week). The following formula was used to calculate L_EX,40y_:

(1) $$L_{\mathrm{EX},40y}{\;=\;L}_{\mathrm{pAeq},\mathrm{Te}}{\;+\;10\;\log\;(T}_{e}{/T}_{0})\;\mathrm{dB}(A),$$

where L_pAeq,Te_ is the average sound level in dB(A), $T_{e}$ is the exposure time in years, and $T_{0}$ is the reference working life ( $T_{0}=\;$ 40 years). Using this formula, L_EX,40y_ is equal to 90 dB(A) after 40 years of exposure to 90 dB(A) and approximately 87 dB(A) after 20 years of exposure to 90 dB(A), because 3 dB(A) represents a doubling in noise energy exposure.

We then used the difference in cumulative noise exposure between the exposed and comparison groups in order to convert the reported risk estimates to a relative risk per 10 dB(A) L_EX,40y_, as follows:

(2) $${\mathrm{RR}}_{\mathrm{per}\;10\mathrm{dB}\left(A\right)\;L_{\mathrm{EX},40y}\;}={\mathrm{RR}}^{(\frac{10\mathrm{dB}\left(A\right)}{{L_{\mathrm{EX},40y}}_{\mathrm{exposed}}{{-L}_{\mathrm{EX},40y}}_{\mathrm{comparison}}}).}$$

For example, Chang et al. [44] found an adjusted PR of 4.66 (converted from OR) for noise exposures exceeding 80 dB(A). The average noise exposure in the exposed group was 84.1 dB(A), and the exposed workers worked for an average of 7.4 years. Based on Formula (1), this corresponds to a L_EX,40y_ of 76.77 dB(A). The average noise exposure in the comparison group was 72.8 dB(A) for an average of 7.6 years, which yields an L_EX,40y_ of 65.60 dB(A). These values and the PR of 4.66 were converted to the increased risk per 10 dB(A) L_EX,40y_ using Formula (2). This resulted in an effect estimate of 3.96 per 10 dB(A) L_EX,40y_. This effect estimate was pooled in a further meta-analysis. The upper and lower limits of the confidence interval were converted using the same formula, which sometimes resulted in asymmetrical confidence intervals.

### 2.6. Assessment of Evidence

We also evaluated the entire body of evidence using the Grading of Recommendations, Assessment, Development and Evaluation (GRADE) approach [45] with *Navigation Guide* adaptations for observational epidemiological studies [46,47]. Accordingly, we used three levels of evidence certainty (low, moderate, and high), and initially rated the body evidence as having moderate quality if the evidence only came from observational studies and high if there was evidence from randomized studies. We downgraded the quality of evidence one or two levels according to the each of the following criteria:

1. study limitations (risk of bias);
2. indirectness of evidence;
3. inconsistency of evidence;
4. imprecision;
5. publication bias;

We upgraded the quality of evidence one or two levels according to the criteria:

6. large magnitude of effect;
7. dose-response gradient; and,
8. residual confounding increases confidence.

We summed the downgrading levels first, and then the upgrading levels to determine the final decision. Otherwise, we applied the rules for assessing evidence outlined in Appendix D of Teixeira et al. [46] for our decisions.

## 3. Results

**Figure 1:**
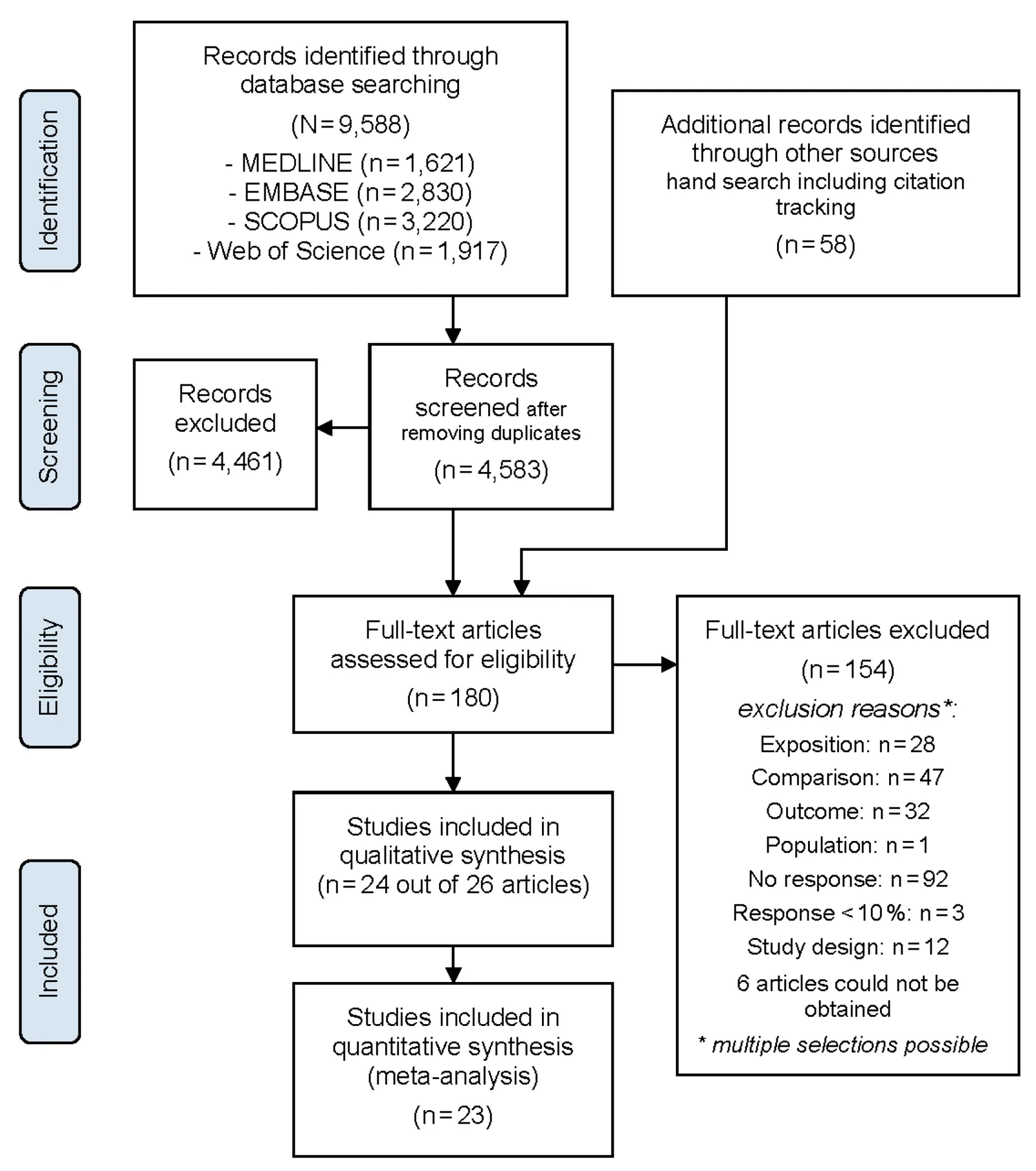
PRISMA Flowchart.

The database search found 4583 unique references (9588 with duplication). Title and abstract screening, citation tracking, and the reference list search identified 180 studies for the full text screening. We included 24 studies published (in 26 publications) between 1977 and 2019 [44,48,49,50,51,52,53,54,55,56,57,58,59,60,61,62,63,64,65,66,67,68,69,70,71,72]. Despite extensive searching, we were unable to obtain the complete text of six publications through our library and excluded 148 studies. The literature selection process is summarized in Figure 1.

For 49 of the excluded studies, we had multiple reasons for exclusion. Most frequently studies did not provide adequate information regarding study response (n = 92). Three additional studies had a response below 10%, indicating that substantial selection or volunteer bias compromised the representativeness of the population. Studies were also excluded because an acceptable comparison group was lacking (n = 47), hypertension was not considered (n = 32), noise exposure was not assessed (n = 27), and/or an inadequate study design was used (n = 12). A list of the excluded studies and the reasons for their exclusion is in the online supplementary (Table S2).

Of the 24 included studies, only one was a case-control study. Six studies were cohort studies and most used a cross-sectional study design (n = 17). Geographically, the study regions were widely dispersed, and the highest number of studies were conducted in Taiwan (n = 5), followed by Italy (n = 3). Two studies each were conducted in Brazil, Russia, and the People’s Republic of China. The following countries were represented by a single study: Denmark, France, India, Indonesia, Iran, Israel, Poland, Republic of Korea, Taiwan (ROC), Tunisia, and Yugoslavia (now Croatia). Fourteen studies were published in English. Ten of the 24 studies (42%) were published in languages other than English: three in Russian, two in French, and one each in Chinese, Italian, Korean, Portuguese, and Serbo-Croatian.

A majority of the studies considered workers in noise-exposed manufacturing or factories (n = 16). Two studies included workers in the oil or minerals mining industry, while air force pilots, workers in the food industry, gas industry workers, merchant marines, power plant workers, and shipyard workers were each the subject of a single study. The comparison groups comprised either groups of less-exposed workers or workers in administrative jobs.

Regarding the sex of the working populations considered, 13 of the studies recruited both male and female participants, although men comprised over 70% of the participants in seven of these studies. One study recruited only female participants [72], six recruited only male participants, and three studies [64,65,67] did not provide any information on the sex of the participants. However, due to the occupations that were considered by these studies and the sex distribution reported by other studies considering comparable occupations, we presume these study populations were predominately, if not entirely, male. Study details are included in the data extraction tables provided as an online supplement (Tables S3–S6).

### 3.1. Risk of Bias

We only judged three studies to have an overall low risk of bias [49,51,61] (Table 2). We could judge the study by Brahem and colleagues [49] to have a low risk of bias after additional information was provided by the first author of this study per e-mail. Most often (18 studies), the chronology was not established due to the cross-sectional study design [44,48,50,52,53,54,55,57,58,60,64,65,66,68,70,72] or due to unknown hypertension status of study participants at baseline in cohort studies [56,62]. The only cross-sectional study to receive a low risk of bias rating for chronology was the study by Brahem et al. [49], because workers who had hypertension when they began working at the electric power station were excluded from the analysis. We rated half of the studies (12 studies) as having a high risk of bias for the exposure definition and measurement [53,54,55,56,57,58,64,66,67,68,70,71]. These studies failed to report details regarding how noise was measured, such as the measurement instrument used, measurement period, and number of measurements used. Nearly as many studies failed to adequately account for confounding due to at least age and sex (nine studies) or used inadequate analysis methods, such as descriptive or bivariate analyses (11 studies).

### 3.2. Meta-Analysis

The meta-analysis combines the results of 23 studies. We excluded the results of the Fogari et al. 1994 [54] publication from the meta-analysis, because the same study population was also the subject of the study published by Fogari et al. [55] in 1995. Although we suspect that the population of aircraft manufacturing workers in Taiwan considered by Hwang et al. [59] might overlap some with the study population considered in the Chang et al. 2013 [51] study of aircraft manufacturing workers in Taiwan, the number of participants differed, so both results are included in the meta-analysis. Hwang et al. [59] stratified workers by considering non-overlapping subpopulations based on work experience, so we included the results of both work-experience subgroups in the meta-analysis.

**Figure 2:**
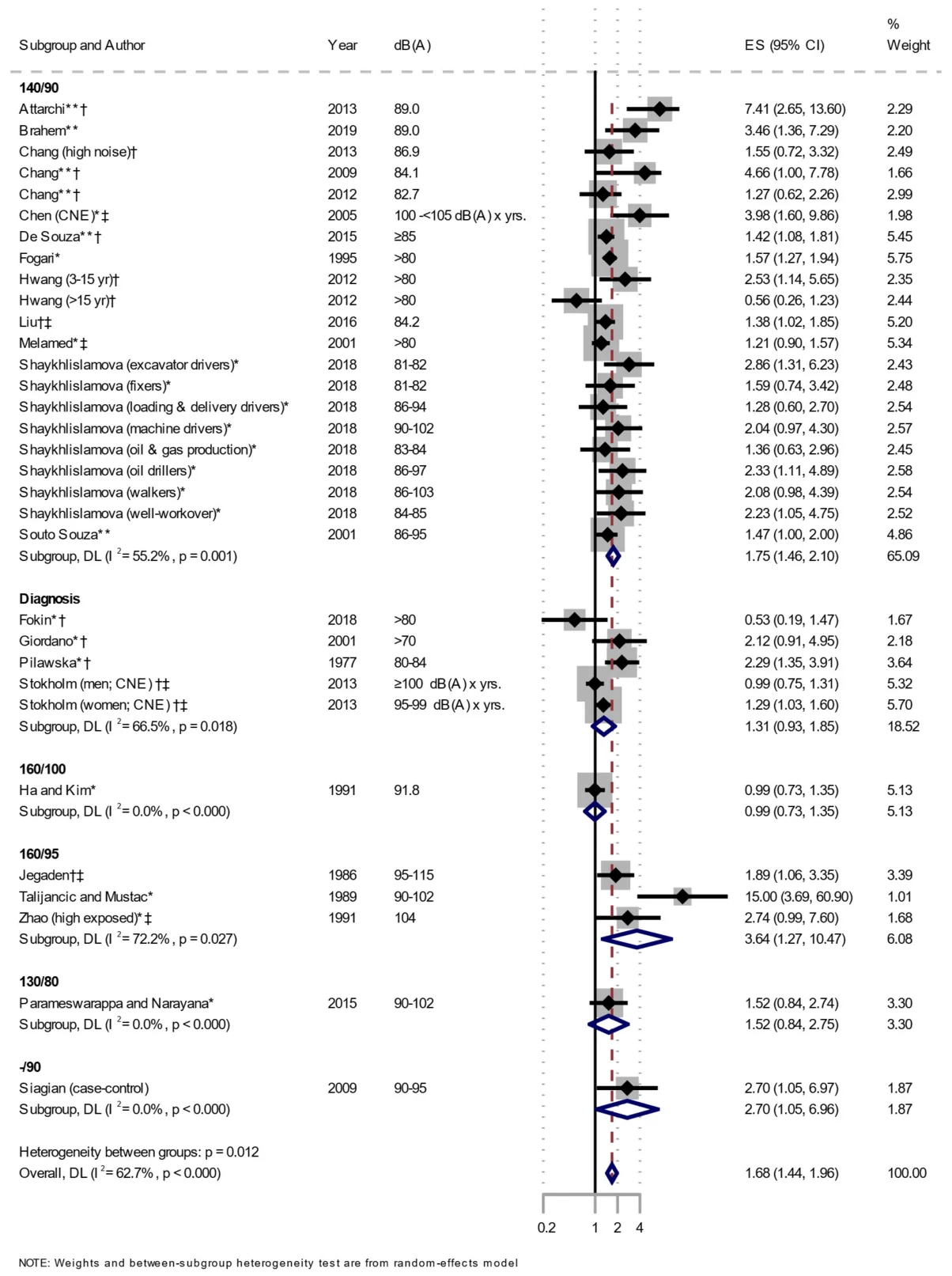
Forest plot for noise exposure >80 dB(A) versus ≤80 dB(A) grouped according to hypertension definitions. Studies marked with * indicate that we calculated the effect size (ES) from the reported prevalences (generally this ES was unadjusted except for job-complexity: Melamed 2001 [62]; age: Ha & Kim 1991 [58], Parameswarappa & Narayana 2015 [64], Giordano 2001 [57]). Studies marked with ** indicate that the odds ratio was corrected to represent the prevalence ratio. † indicates that a physician diagnosis of hypertension was included in hypertension definition, and ‡ indicates that anti-hypertensive use was included in the hypertension definition [44,48,49,50,51,52,53,55,56,57,58,59,60,61,62,64,65,66,67,68,69,70,72].

Chang et al. [51], Hwang et al. [59], Liu et al. [61], and Stokholm et al. [69] reported the RR as incidence rate ratios calculated by considering the incidence per person-years of the workers observed in the study (incidence density). Eleven studies reported the prevalence of hypertension in the exposed and comparison groups, which we used to calculate the prevalence ratios (PR) and 95% confidence intervals. Six studies assessed the noise-related hypertension risk using OR, which we converted to PR for inclusion in the meta-analysis. One study was a case-control study [67], and the OR could not be converted to a PR, because information on the prevalence of hypertension among the less-exposed group was not available. Figure 2 shows the meta-analysis of all studies grouped according to their hypertension definition.

The threshold of 140 mmHg systolic and/or 90 mmHg diastolic blood pressure was used most frequently (n = 13 studies) in order to define hypertension. The resulting pooled risk estimate for occupational noise exposure exceeding 80 dB(A) in this group was 1.75 (95% CI 1.46–2.10). Moderate heterogeneity (I^2^ = 55.2%) was observed, but the heterogeneity was lower in this subgroup as compared to other diagnosis subgroups with two or more studies. The test for heterogeneity was statistically significant (*p* = 0.001) for this diagnosis definition, indicating the presence of heterogeneity. However, this might be due in part to large number of effect estimates included in this group increasing the test power.

Studies identifying hypertension without directly measuring blood pressure did so by either asking about physician-diagnosed hypertension, consulting company health records, or by consulting national records on prescription antihypertensive drug use and hospital-diagnosed diseases [69]. The pooled effect estimate for physician diagnosed hypertension and/or medication use was not statistically significant (ES = 1.31; 95% CI 0.93–1.85). There was also substantial heterogeneity (I^2^ = 66.5%) in this subgroup.

Of the remaining hypertension definitions used, the only threshold that was used by more than two studies was that of 160 mmHg systolic and/or 95 mmHg diastolic blood pressure. This definition resulted in the highest pooled risk, but it should be noted that the effect sizes for this group also represented higher noise exposures above 90 dB(A) and there was considerable heterogeneity (I^2^ = 72.2%).

**Figure 3:**
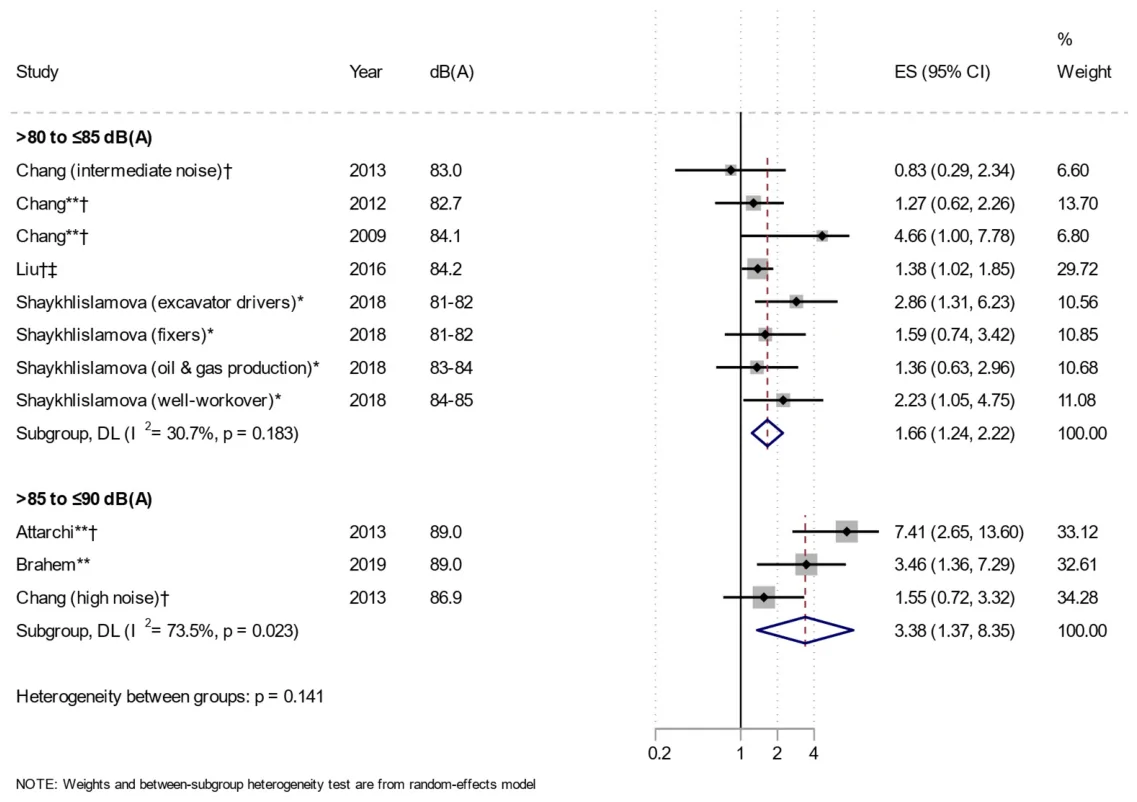
Forest plot for noise exposure in the range of >80 to ≤85 dB(A) and >85 to ≤90 dB(A) using the 140/90 hypertension definition. Studies marked with * indicate that we calculated the effect size (ES) from the reported prevalence. Studies marked with ** indicate that the odds ratio was corrected to represent the prevalence ratio. † indicates that a physician diagnosis of hypertension was included in hypertension definition, and ‡ indicates that anti-hypertensive use was included in the hypertension definition [44,48,49,50,51,61,66].

We also considered subgroups of effect estimates for comparable exposure ranges while using similar hypertension definitions (Figure 3). Only the 140/90 mmHg hypertension definition was used frequently enough to permit comparisons between noise in the range of over 80 to ≤85 dB(A) and noise exceeding 85 to ≤90 dB(A). We grouped the corresponding risk estimates according to the mean value of the reported upper and lower exposure limits if studies reported noise-ranges instead of average noise exposure. None of the studies reporting risk estimates for average noise exposures exceeding 90 dB(A) used the 140/90 mmHg hypertension definition, so noise exceeding 90 dB(A) was not considered in this subgroup analysis. Occupational noise exposure in the >80 to ≤85 dB(A) range corresponded with a pooled risk estimate of 1.66 (95% CI 1.24–2.22, I^2^ = 30.7%). Both the pooled risk estimate and the heterogeneity increased for exposures in the 85 to ≤90 dB(A) range, indicating a dose-response relationship. A descriptive depiction of the effect estimates reported by studies that looked at more than one exposure level also supports the possibility of a dose-response relationship (Supplementary Figure S1). Additionally, a sensitivity analysis of studies reporting hypertension risks for noise exposures < 80 dB(A) and using the 140/90 mmHg hypertension definition did not find a statistically significant increase in risk (ES = 1.21; 95% CI 0.78–1.87) for lower exposure levels (Supplementary Figure S2).

**Figure 4:**
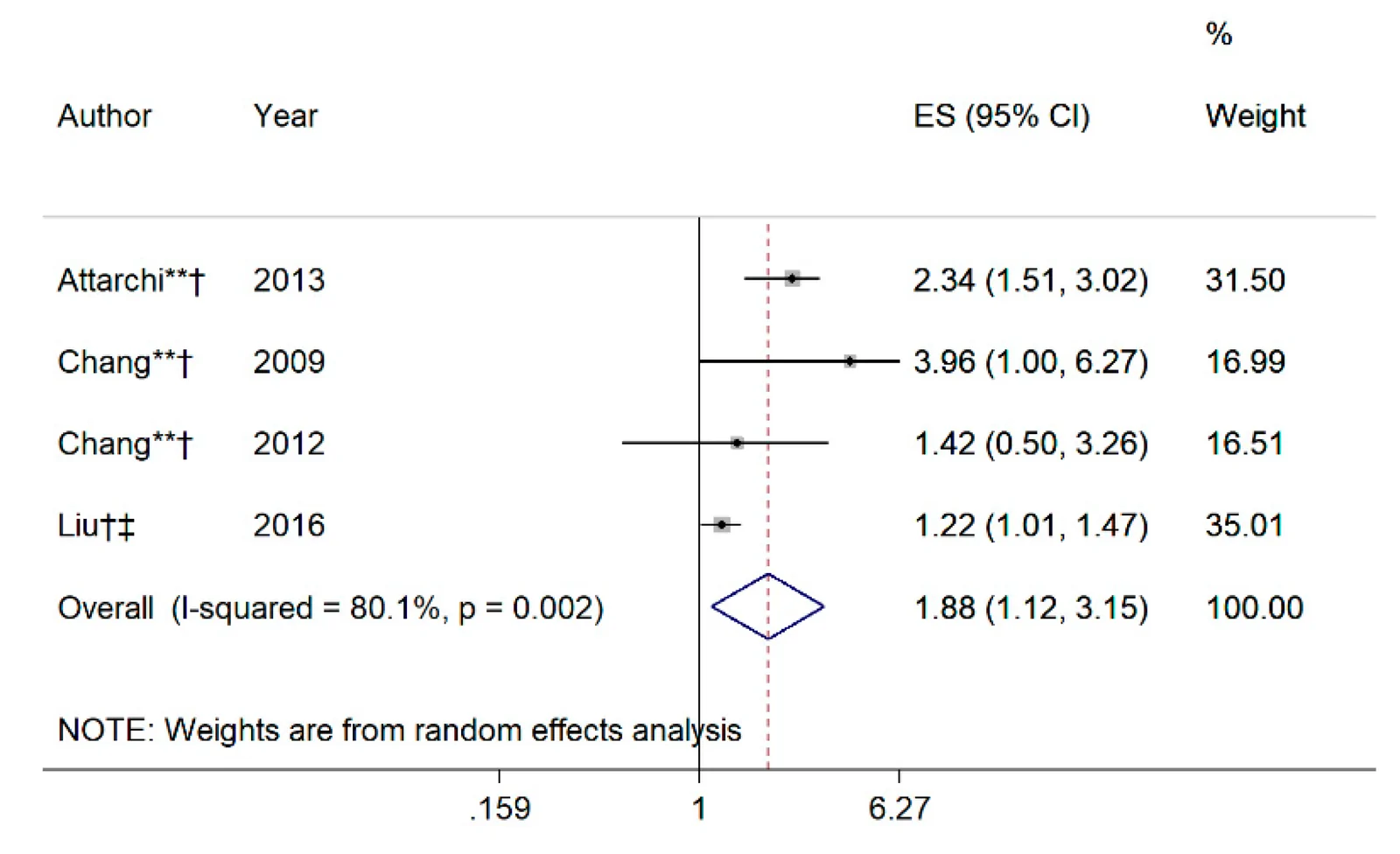
Forest plot of risk per 10 dB(A) L_EX,40y_ among studies where the average noise and duration of employment was reported for both the exposure and comparison groups, and where hypertension was defined as blood pressure exceeding 140/90 mmHg. The effect estimates of studies marked with * indicate that effect size (ES) were calculated from the reported prevalence, and studies marked with ** indicate that the odds ratio was corrected to represent the prevalence ratio [44,48,50,61].

The cumulative risk estimates that were based on a 10 dB(A) increase in noise over a 40-year working life (L_EX,40y_) were pooled for the four studies using the 140/90 mmHg definition of hypertension and reporting sufficient information regarding average noise exposure and duration of employment/exposure (Figure 4). The pooled estimate showed an increase risk of 88% per 10 dB(A) L_EX,40y_ and was statistically significant (ES = 1.88; 95% CI 1.12–3.15). We did observe considerable heterogeneity (I^2^ = 80.1%) for this analysis.

**Figure 5:**
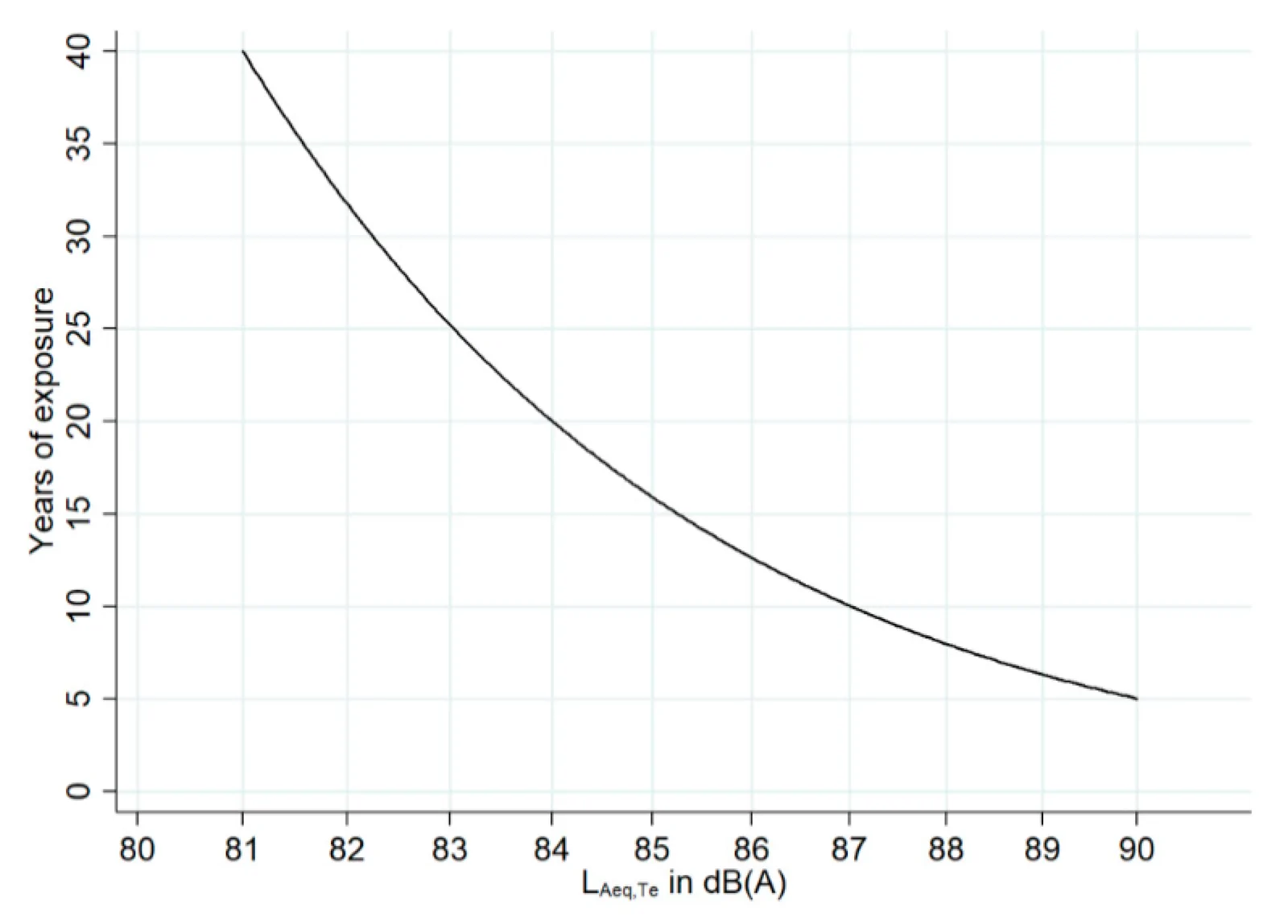
Noise exposure levels L_Aeq,Te_ in dB(A) and durations of exposure in years resulting in a doubling of risk.

Examples of L_EX,40y_ for various exposure levels and durations of exposure are shown in Table A1 of Appendix A in order to illustrate what an increase in noise normalized to a 40 year working life comprises in terms of intensity, duration of exposure and hypertension risk. The relative risks shown in Table A1 were calculated based on an increase in L_EX,40y_ as compared to a 40 year-exposure to 70 dB(A). A reference level of L_EX,40y_ = 70 dB(A) was chosen, because this was the average exposure of the comparison groups used in the studies depicted in Figure 4. Although workers in the comparison groups were exposed for an average of about seven years, if we presume there is no increase in noise-related hypertension risk at 70 dB(A) this should also be true for 40 years. Using this threshold, an exposure to 85 dB(A) doubles the risk of hypertension after 15.9 years (Figure 5).

**Figure 6:**
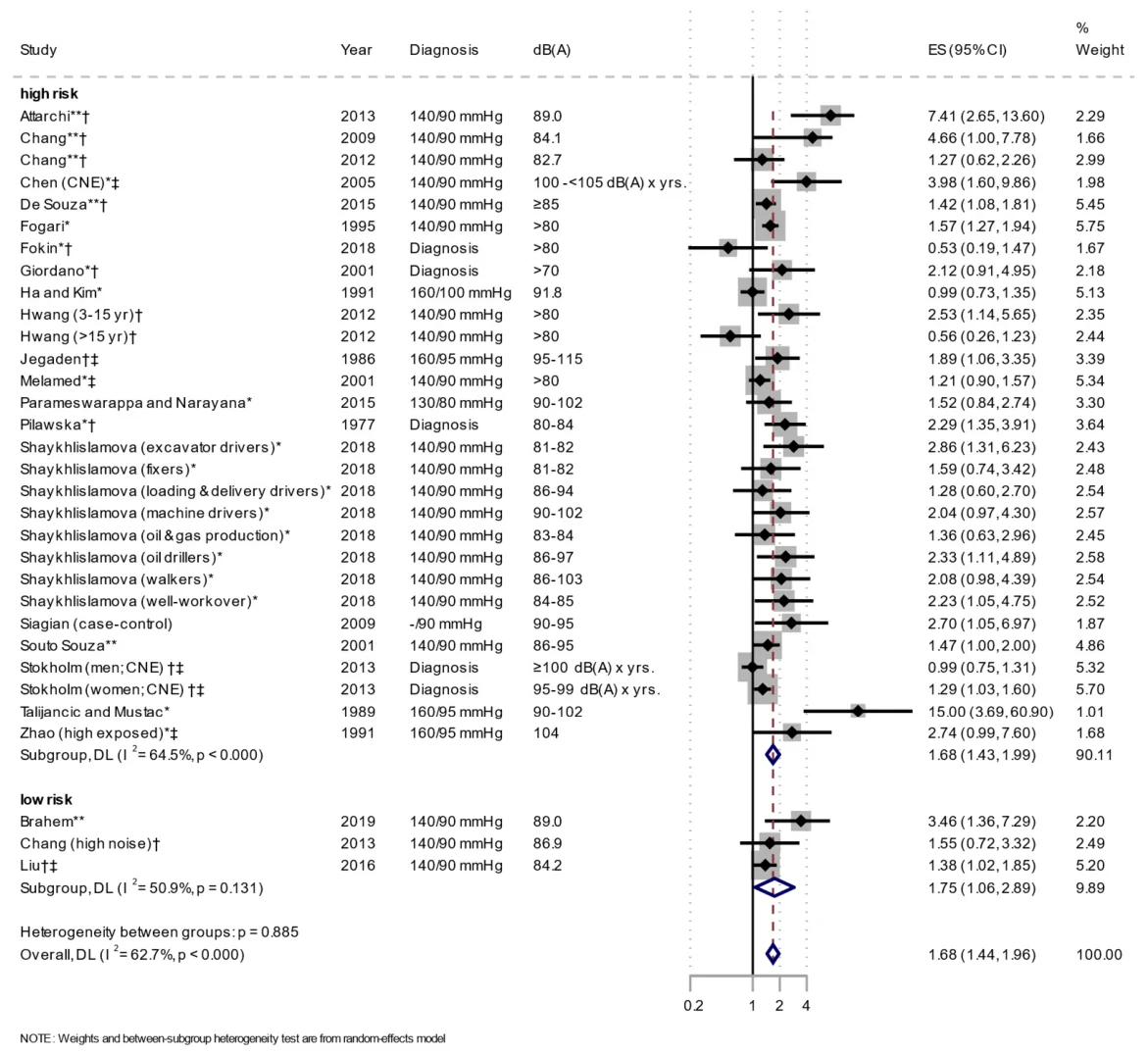
Forest plot of study results stratified by risk of bias. Studies marked with * indicate that we calculated the effect size (ES) from the reported prevalence. Studies marked with ** indicate that the odds ratio was corrected to represent the prevalence ratio. † indicates that a physician diagnosis of hypertension was included in hypertension definition, and ‡ indicates that anti-hypertensive use was included in the hypertension definition [44,48,49,50,51,52,53,55,56,57,58,59,60,61,62,64,65,66,67,68,69,70,72].

We also stratified the results according to risk of bias while disregarding differing definitions of hypertension (Figure 6). All three studies with an overall low risk of bias used the 140/90 mmHg definition of hypertension, and their pooled effect estimate was 1.75 (95% CI 1.06–2.89, I^2^ = 50.9%) for occupational noise exposures exceeding 80 dB(A). In comparison, the pooled estimate for studies with an overall high risk of bias was lower (1.68; 95% CI 1.43–1.99), heterogeneity greater (I^2^ = 64.5%), and these studies applied different definitions of hypertension.

**Figure 7:**
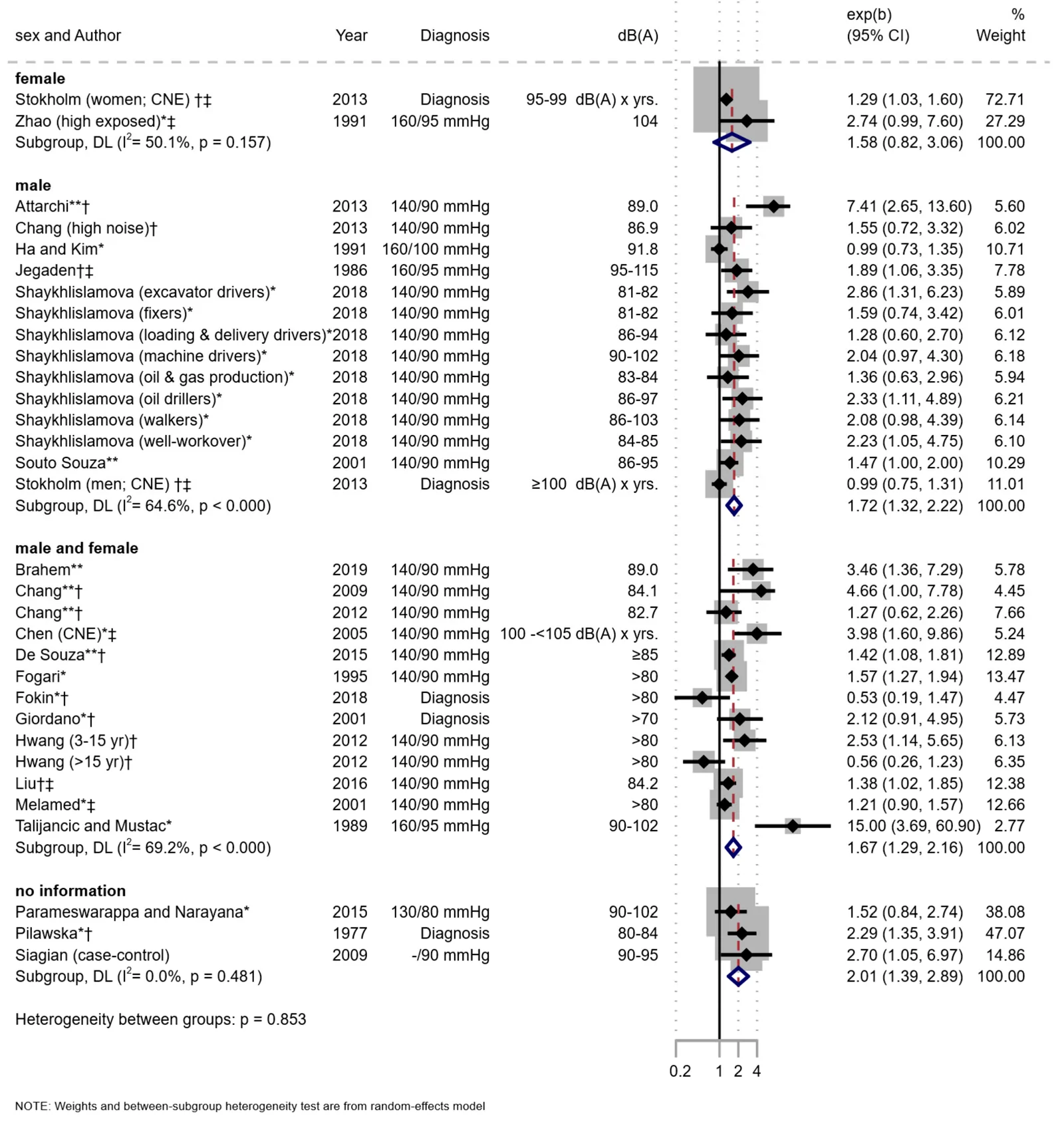
Forest plot depicting study results stratified by sex. Studies marked with * indicate that we calculated the effect size (ES) from the reported prevalence. Studies marked with ** indicate that the odds ratio was corrected to represent the prevalence ratio. † indicates that a physician diagnosis of hypertension was included in hypertension definition, and ‡ indicates that anti-hypertensive use was included in the hypertension definition [44,48,49,50,51,52,53,55,56,57,58,59,60,61,62,64,65,66,67,68,69,70,72].

The stratification according to sex of the study population is shown in Figure 7. This analysis showed comparable risk increases for worker populations including only men (ES = 1.72; 95% CI 1.32–2.22; I^2^ = 64.6%) and those including both men and women (ES = 1.67; 95% CI 1.29–2.16; I^2^ = 69.2%). Only two studies provided risk estimates for women. Only Zhao et al. [72] included only women and Stokholm et al. [69] reported results that were stratified by sex. Both of these studies indicate an increased risk of hypertension among women exposed to occupational noise, although the Zhao study just failed to reach statistical significance. We refrained from pooling the results for women due to the low number of studies and the use of different hypertension definitions by these two studies.

The leave-one-out sensitivity analysis resulted in pooled overall estimates (all studies shown in Figure 2) that decreased to 1.64 (95% CI 1.42–1.89) when Attarchi et al. [48] was excluded, and increased to 1.81 (95% CI 1.54–2.13) when the Stokholm et al. [69] study was excluded (Supplementary Table S7). Regardless of the studies excluded, the results remained statistically significant.

In a further sensitivity analysis, we looked at the effect of study design on studies using the 140/90 mmHg hypertension definition (Supplementary Figure S3). The combined effect increased when cross-sectional studies were considered separately (ES = 1.97; 95% CI 1.60–2.42, I^2^ = 50.7%). The pooled effect was attenuated for the four cohort studies (ES = 1.29; 95% CI 0.95–1.75, I^2^ = 48.6%).

### 3.3. Publication Bias

**Figure 8:**
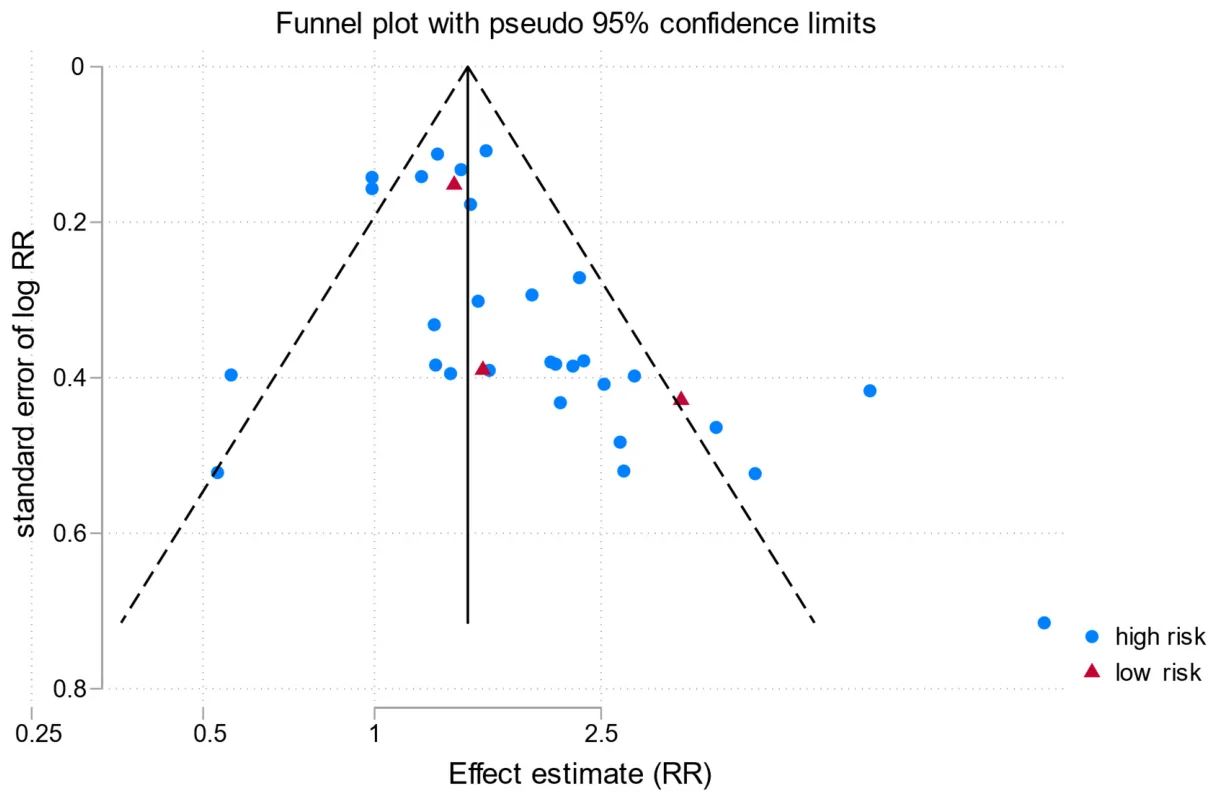
Funnel plot of effect estimates included in the main analysis (Figure 2).

A funnel plot of the effect estimates used in the main analysis (Figure 2) showed an asymmetrical distribution of the effect estimates (Figure 8). The statistically significant Egger’s Test (*p* = 0.001) also indicated small-study effects. We used the "trim and fill" method (using *metatrim*) to determine what the pooled effect would have been if the “missing” study results were available in order to gauge the potential impact of publication bias on the pooled results. According to this method, the pooled RR (random-effects) would still be significant if the funnel plot were symmetrical (corrected RR = 1.38; 95% CI 1.17–1.63).

### 3.4. Quality of Evidence Assessment

The assessment of evidence resulted in an overall high quality of evidence (Table 3). We downgraded one level for publication bias, due to the asymmetry of the funnel plot (Figure 8). We detected a positive dose-response gradient in the subgroup analysis of different noise exposure levels and in single studies considering several levels of noise (Supplementary Figure S1). Thus, we upgraded one level for dose-response gradient. We also upgraded for effect size, because the effect size was >2 in the subgroup of workers exposed to >85–≤90 dB(A) (ES = 3.38; 95% CI 1.37–8.35).

We did not downgrade for imprecision although the measured heterogeneity in the main analysis was substantial (I^2^ > 50 %) and the χ^2^-test indicated heterogeneity (*p* < 0.001). Statistical tests for heterogeneity tend to be overpowered when many larger studies are included in the meta-analysis [73]. Therefore, we based our decision on the fact that the confidence limits of the studies overlapped, and the heterogeneity of homogenous subgroups was generally lower and sometimes I^2^ < 50%. For instance, the lower heterogeneity in the subgroup analysis of noise exposures in the range of >80–≤85 dB (I^2^ = 30.7%) indicates that the noise exposure level explains part of the heterogeneity.

## 4. Discussions

The present systematic review identified 24 epidemiological studies and the meta-analysis of 23 of these studies indicates a significantly increased risk of hypertension by a factor of 1.68 (95% CI 1.44–1.96) (Figure 2).

### 4.1. Strengths and Limitations

One of the strengths of the present systematic review is that the title-abstract screening, data extraction, and quality assessment were carried out independently by two authors. There were no restrictions for the review regarding the language of the publication, and ten of the 24 epidemiological studies included were published in a language other than English. Even unpublished studies (grey literature) had the chance to be included in the review. The study design was published *a priori* in PROSPERO.

The conclusiveness of this systematic review is limited by the predominantly low methodological quality of the included epidemiological studies. Only three of the 24 included studies had an overall low risk of bias. However, even in these methodologically sound studies, the risk of hypertension was found to be significantly increased by a factor of 1.75 (95% CI 1.06–2.89) (Figure 6).

A further limitation of the present systematic review is the strongly divergent definition of hypertension applied by the included studies. The limit values used were 130/80 mmHg (n = 1), 140/90 mmHg (n = 13), a diastolic blood pressure of more than 90 mmHg (n = 1), 160/95 mmHg (n = 3), 160/100 mmHg (n = 1), or a diagnosis of hypertension with health insurance records or disease registers (n = 4). In the largest group of studies with a definition of 140/90 mmHg, the risk of hypertension was significantly increased by a factor of 1.75 (95% CI 1.46–2.10) in the meta-analysis (Figure 2).

One factor that could potentially confound the relationship between occupational noise exposure and hypertension is environmental noise exposure. However, none of the included studies provide any information on the environmental noise exposure of the investigated employees. Although confounding is theoretically possible, we are of the opinion that environmental noise exposures have no substantial effect on the results described here, because, according to the systematic review by van Kempen et al. [74], there is only very low to low quality evidence of an association between environmental noise and hypertension, and the observed risks are low. For example, van Kempen et al. [74] concluded that the relative risk per 10 dB in subjects exposed to road traffic noise was 1.05 (95% CI 1.02–1.08) in cross-sectional studies and 0.97 (95% CI 0.90–1.05) in a cohort study.

### 4.2. Dose-Response Relationship

Several results of our systematic review speak for a dose-response relationship between occupational noise exposure and the risk of hypertension. For one, employees with an occupational noise exposure of >80–≤85 dB(A) had a significantly increased risk of hypertension by a factor of 1.66 (95% CI 1.24–2.22) in the meta-analysis, whereas this risk was significantly increased by a factor of 3.38 (95% CI 1.37–8.35) for employees with a noise exposure of >85–≤90 dB(A) (Figure 3). This meta-analysis only included studies that considered hypertension at 140/90 mmHg. Three included studies looked at workers exposed to noise above 90 dB(A) defining hypertension at the level of 160/95 mmHg. These three studies found a significantly increased risk of hypertension by a factor of 3.64 (95 % CI 1.27–10.47) (Figure 2). The results in Table A1 also suggest that workers exposed to 90 dB(A) for five years, 85 dB(A) of noise for 15–20 years or 83 dB(A) for 20–30 years have approximately twice the risk of hypertension.

### 4.3. Discussion of the Stokholm et al. 2013 Study

In terms of sample size, Stokholm et al. [69] conducted the largest epidemiological study, and found a significant association between occupational noise exposure and hypertension only in women. The authors presented the results of a cohort study of 108,402 male and 36,788 female employees in Denmark, employed in 725 companies. 625 companies belonged to the 10 sectors with the highest risk of noise hearing loss in Denmark. The comparison group comprised 100 companies in the financial sector without any relevant occupational noise exposure. In 2001, noise exposure was measured in 80 randomly selected industrial companies with 649 employees during a working shift. In the years 2009–2010, this measurement was repeated for 589 employees in 132 companies. Cumulative occupational noise exposure [CNE, dB(A) × years] was calculated according to formula (3)

(3) $$\mathrm{CNE}=10\;\times \log_{10}\left(\sum 10^{\frac{L_{\mathrm{Aeq}}}{10}}\;\times t\right),$$

where L_Aeq_ is the average sound level in dB(A) and t is the exposure time in years. Treatments with antihypertensive drugs (i.e., alpha-2 agonists, diuretics, beta-blockers, calcium channel blockers, and ACE inhibitors) registered in the Danish Register of Prescribed Drugs and inpatient hypertension diagnoses recorded in the national hospital discharge register were considered. Based on these two registries, 7587 hypertension cases were recorded in the cohort. In the study, the relative risks of hypertension among industrial workers as compared to unexposed bank employees, as well as based on cumulative noise exposures, adjusted for age, socioeconomic status, and (possibly) exposure duration were assessed. The study found a positive dose-response relationship in men as a function of cumulative noise exposure with a relative risk of 4.66 (95% CI 3.63–5.97) at >100 [dB(A) × years], which disappeared after adjustment (relative risk 0.99 [95% CI 0.75–1.31)]. In women, a positive dose-response relationship was also found with a significantly increased risk of hypertension by a factor of 2.4 (95% CI 1.99–2.89) in the highest dose class of 95–99 [dB(A) × years]. After adjustment, the risk of hypertension was attenuated but still significantly increased by a factor of 1.29 (95% CI 1.03–1.60) in this dose class. It could not be determined whether fully adjusted models adjusted for exposure duration. If so, this may be an over-adjustment that is obscuring the positive dose-response relationship. Furthermore, individual noise exposure is known only among 649 of the 145,190 cohort members (4.5%), so there may be non-differential misclassification regarding occupational noise exposure. In addition, the study can only correctly estimate risk if the employees exposed to noise visit a doctor and are treated for hypertension with the same frequency as the comparison group of bank employees. This was not investigated in the study. Due to their higher social status, financial sector employees might visit a doctor and receive treatment for hypertension more frequently than noise-exposed industrial workers. A cross-sectional study by Fouriaud et al. [75] of French employees supports the link between social status and treatment for hypertension. At the time of the study, 35.6% of the male subjects with hypertension in the highest social class and 19.2% of the subjects in the lowest social class were treated with antihypertensive drugs (*p* < 0.05). Compliance with antihypertensive therapy was significantly higher in the highest social class (73%) than in the lowest social class (40%). Although Stokholm et al. [69] adjusted their analysis for social status in 2013, it is questionable whether this reliably eliminated the above-mentioned error source of a potentially higher treatment prevalence for hypertension among financial sector employees with higher social status as compared to noise-exposed workers.

### 4.4. Evidence of Causality

Overall, the present systematic review found clear evidence of a relationship between occupational noise exposure and the risk of hypertension. We assess the causality between exposure to occupational noise and the development of hypertension according to Hill [76], as follows:

(a) Mechanistic studies demonstrate an increased excretion of blood pressure-increasing hormones, such as adrenaline and noradrenaline in noise-exposed workers [26,27], and animal studies [6,7,8,9,10,11,12,13,14,15,16,17,18,19,20] support the biological plausibility of the link.
(b) Further evidence of a dose-response relationship between the level of occupational noise exposure and risk of hypertension supports the association.
(c) There is a strong association between the level of exposure to occupational noise and the risk of hypertension, which is more than three times higher for workers exposed to noise above 85 dB(A).
(d) The relationship between occupational noise exposure and increased risk is consistent and it has been observed in a large number of epidemiological studies.
(e) The temporal relationship between occupational noise exposure and hypertension has been established in several cohort studies.

Altogether, the available evidence suggests that there is a causal relationship between exposure to occupational noise of at least 80 dB(A), and we recommend hypertension in highly noise-exposed workers be considered an occupational disease.

## 5. Conclusions

In this systematic review, clear evidence of a dose-response relationship was found between occupational noise exposure and hypertension risk. Above 85 dB(A), the risk of developing hypertension was more than three times higher relative to the comparison group. There is no substantial risk difference between men and women, but data concerning this question are limited. There is high certainty of evidence that occupational noise exposure increases the risk of hypertension.

## Figures and Tables

**Table 1:** Eligibility criteria according to population, exposure, comparison, outcome of interest, and study design.

	Inclusion	Exclusion
**Population** **(P)**	General working population	Children and animals
**Exposure** **(E)**	Quantified occupational noise exposure in dB	No quantification of occupational noise exposure in dB
**Comparison** **(C)**	General working population or specific groups of workers with noise exposures L_EX,8h_ ≤80 dB	No adequate comparison group
**Outcome** **(O)**	Primary arterial hypertension (ICD10 = I10) defined using at least one of the following criteria: Arterial systolic blood pressure measured by the method of Riva Rocci ≥130 mmHg ^1^ Arterial systolic blood pressure determined as part of a 24-h blood pressure measurement ≥130 mmHg Diastolic blood pressure measured by the method of Riva Rocci ≥80 mmHg^1^ Diastolic blood pressure determined as part of a 24-h blood pressure measurement ≥80 mmHg Physician diagnosed arterial hypertension Drug treatment for arterial hypertension	No identification/diagnosis of hypertension using the definitions outlined in the inclusion criteria column
**Study Design** **(S)**	cohort, case-control, cross-sectional, case-cohort, nested-case-control studies with a response ≥10%	qualitative studies, case descriptions, ecological studies, experimental studies, letters to the editor, comments/editorials, congress abstracts/posters, reviews; epidemiological studies not reporting response or with response <10%

^1^ This includes hypertension definition using higher values, such as systolic blood pressure ≥140 mmHg.

**Table 2:** Risk of bias assessment.

Study	Study Type	Major Domains						Minor Domains			OVERALL
		Recruitment & Follow-up (Cohort)	Exposure Definition & Measurement	Outcome Assessment & Validation	Con-Foun-Ding & Effect Modification	Analysis Method	Chronology	Assessor Blinding	Funding	Conflict of Interest	
Attarchi 2013 [48]	CS	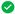	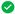	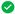	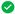	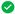	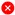	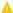	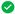	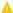	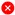
Brahem 2019 [49]	CS	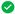	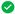	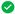	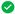	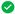	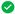	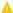	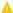	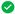	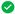
Chang 2009 [44]	CS	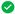	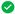	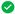	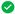	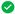	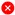	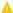	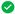	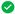	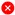
Chang 2012 [50]	CS	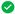	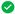	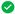	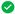	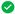	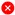	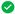	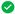	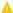	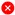
Chang 2013 [51]	Co	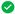	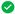	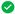	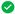	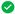	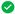	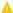	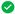	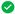	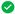
Chen 2005 and Pang 2005 [52,63]	CS	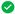	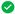	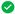	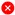	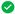	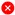	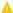	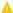	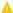	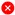
De Souza 2015 [53]	CS	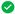	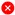	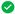	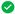	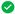	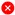	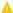	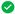	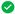	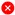
Fogari 1994 [54] ^§^	CS	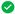	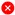	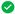	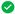	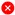	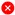	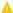	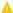	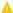	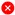
Fogari 1995 [55]	CS	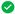	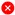	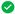	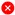	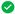	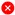	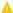	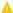	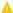	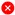
Fokin 2018 [56]	Co	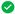	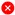	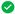	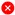	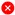	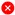	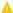	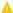	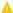	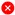
Giordano 2001 [57]	CS	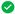	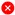	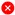	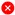	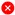	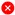	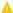	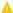	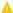	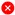
Ha & Kim 1991 [58]	CS	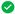	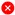	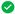	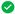	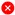	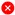	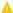	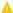	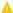	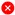
Hwang 2012 [59]	Co	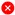	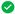	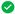	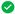	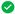	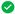	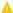	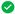	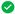	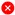
Jegaden 1986 [60]	CS	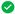	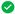	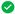	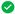	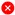	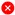	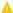	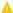	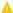	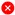
Liu 2016 [61]	Co	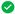	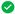	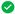	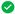	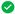	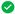	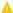	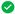	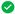	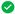
Melamed 2001 [62]	Co	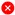	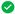	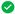	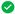	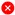	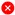	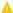	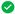	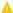	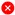
Parameswarappa 2015 [64]	CS	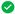	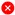	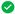	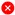	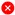	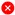	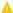	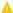	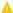	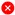
Pilawska 1977 [65]	CS	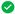	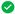	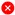	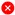	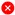	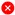	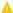	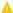	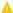	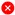
Shaykhlislamova 2018 [66]	CS	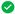	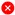	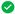	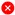	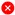	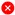	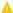	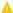	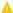	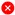
Siagian 2009 [67]	Nested CC	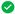	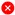	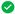	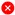	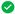	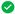	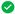	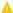	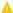	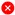
Souto Souza 2001 [68]	CS	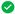	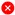	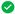	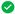	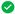	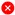	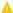	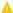	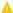	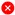
Stokholm 2013 [69]	Co	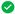	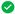	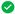	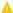	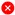	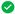	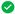	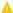	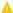	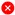
Talijancic 1989 [70]	CS	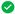	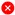	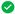	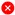	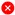	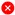	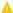	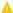	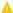	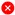
Zhao 1991 and 1993 [71,72]	CS	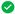	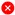	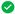	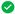	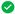	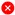	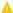	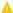	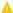	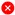

CS Cross-sectional; Co Cohort; CC Case-Control; 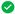 Low Risk; 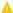 Unclear Risk; 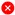 High Risk; § not in meta-analysis.

**Table 3:** Assessment of the Quality of Evidence

	**Question:** Are workers chronically exposed to noise at work with an exposure intensity of >80 dB(A) at an elevated risk for developing arterial hypertension?									
**№ of Studies (Participants)**	**1. Study limitations**	**2. Indirectness**	**3. Inconsistency**	**4. Imprecision**	**5. Publication Bias**	**6. Effect size**	**7. Dose-response Gradient**	**8. Residual Confounding**	**Effect Size**	**Certainty**
*23 observational studies* *(n = 171 985)* ^1^	*not serious* ^2^	*not serious*	*not serious* ^3^	*not serious*	*Detected*^4^ ↓	*Detected* ^5^ *↑*	*Detected* ^6^ *↑*	*no*	RR 1.72 (1.48 to 2.01)	⨁⨁⨁ HIGH

^1^ This is the total number of participants included in our main analysis, comprising of n = 111,796 noise exposed workers and 59,669 workers in comparison groups, as well as 40 cases and 480 control persons from the one case-control study; ^2^ While a majority of the studies had a high risk of bias, considering the pooled effect of low risk of bias studies increased the risk estimate (RR 1.75; 95% CI 1.06–2.89); ^3^ Although the measured heterogeneity in the main analysis was substantial (I^2^ > 50 %) and the χ^2^ test of indicated heterogeneity (*p* < 0.001), the confidence limits overlapped, and the heterogeneity of homogenous subgroups was generally lower and sometimes I^2^ < 50%; ^4^ The funnel plot indicated asymmetry; ^5^ The pooled effect of the main analysis (ES) did not exceed 2.0 (ES = 1.68; 95% CI 1.44–1.96; Figure 2), but ES in the subgroup of workers exposed to >85–≤90 dB(A) was 3.38 (95% CI 1.37–8.35); ^6^ The subgroup analysis of different noise exposure levels (Figure 3) and the descriptive depiction of studies considering several levels of noise indicate a dose-response gradient (Supplementary Figure S1).

## Supplementary Materials

The following are available online at https://www.mdpi.com/1660-4601/17/17/6281/s1, Table S1. Search string for Medline (via Ovid), Table S2. Excluded studies, Table S3. Characteristics of included case-control studies, Table S4. Results shown in included case-control studies; Table S5. Characteristics of included cohort and cross-sectional studies, Table S6. Results shown in included cohort and cross-sectional studies, Table S7: Risk of bias schema, Table S8: Leave-one-out analysis, Figure S1. Forest plot of study results from studies reporting risks for several exposure levels, Figure S2. Forest plot of lower occupational noise exposure levels, Figure S3. Forest plot of study results from cross-sectional studies versus cohort studies using the 140/90 mmHg hypertension definition.

## Appendix A

**Table A1:** Examples of increasing L_EX,40y_ and corresponding increasing risk of hypertension based on a reference of exposure to 70 dB(A) for 40 years.

Exposure in dB(A)	Years of Exposure	L_EX,40y_	RR L_Aeq(40y)_-70 dBA (RR per 10dBA = 1.88)
70	40	70.00	1.00 (Reference)
80	5	70.97	1.06
80	10	73.98	1.29
80	15	75.74	1.44
80	20	76.99	1.55
80	30	78.75	1.74
80	40	80.00	1.88
81	5	71.97	1.13
81	10	74.98	1.37
81	15	76.74	1.53
81	20	77.99	1.66
81	30	79.75	1.85
81	40	81.00	2.00
82	5	72.97	1.21
82	10	75.98	1.46
82	15	77.74	1.63
82	20	78.99	1.76
82	30	80.75	1.97
82	40	82.00	2.13
83	5	73.97	1.28
83	10	76.98	1.55
83	15	78.74	1.74
83	20	79.99	1.88
83	30	81.75	2.10
83	40	83.00	2.27
84	5	74.97	1.37
84	10	77.98	1.65
84	15	79.74	1.85
84	20	80.99	2.00
84	30	82.75	2.24
84	40	84.00	2.42
85	5	75.97	1.46
85	10	78.98	1.76
85	15	80.74	1.97
85	20	81.99	2.13
85	30	83.75	2.38
85	40	85.00	2.58
86	5	76.97	1.55
86	10	79.98	1.88
86	15	81.74	2.10
86	20	82.99	2.27
86	30	84.75	2.54
86	40	86.00	2.75
87	5	77.97	1.65
87	10	80.98	2.00
87	15	82.74	2.24
87	20	83.99	2.42
87	30	85.75	2.70
87	40	87.00	2.92
88	5	78.97	1.76
88	10	81.98	2.13
88	15	83.74	2.38
88	20	84.99	2.58
88	30	86.75	2.88
88	40	88.00	3.12
89	5	79.97	1.88
89	10	82.98	2.27
89	15	84.74	2.54
89	20	85.99	2.74
89	30	87.75	3.07
89	40	89.00	3.32
90	5	80.97	2.00
90	10	83.98	2.42
90	15	85.74	2.70
90	20	86.99	2.92
90	30	88.75	3.27
90	40	90.00	3.53
