# Correction: Bolm-Audorff et al. Occupational Noise and Hypertension Risk: A Systematic Review and Meta-Analysis. *Int. J. Environ. Res. Public Health* 2020, *17*, 6281

**DOI:** 10.3390/ijerph23070855

**Published:** 2026-06-30

**Authors:** Ulrich Bolm-Audorff, Janice Hegewald, Anna Pretzsch, Alice Freiberg, Albert Nienhaus, Andreas Seidler

**Affiliations:** 1 Division of Occupational Health, Department of Occupational Safety and Environment, Regional Government of South Hesse, 65197 Wiesbaden, Germany; ulrich.bolm-audorff@rpda.hessen.de; 2 Associate Professor of Occupational Medicine, Justus-Liebig-University, 35392 Giessen, Germany; 3 Institute and Policlinic of Occupational and Social Medicine (IPAS), Faculty of Medicine, Technische Universität Dresden, 01307 Dresden, Germany; anna.pretzsch@mailbox.tu-dresden.de (A.P.); alice.freiberg@tu-dresden.de (A.F.); ArbSozPH@mailbox.tu-dresden.de (A.S.); 4 Institute of Sociology, Faculty of Behavioral and Social Sciences, Chemnitz University of Technology, Thüringer Weg 9, 09126 Chemnitz, Germany; 5 Competence Center for Epidemiology and Health Services Research for Healthcare Professionals (CVcare), Institute for Health Services Research in Dermatology and Nursing (IVDP), University Medical Centre Hamburg-Eppendorf, 20246 Hamburg, Germany; a.nienhaus@uke.de; 6 Department of Occupational Medicine, Hazardous Substances and Public Health, Institution for Statutory Accident Insurance and Prevention in the Health and Welfare Services (BGW), 22089 Hamburg, Germany

In 2020, we published a systematic review in this journal in which we found a positive dose–response relationship between occupational noise exposure and arterial hypertension [1]. In our original meta-analysis in Figure 6, we included the risk estimates from the cohort study published by Chang et al. [2]. The results of the 2013 publication excluded 10 workers who self-reported a physician hypertension diagnosis or antihypertensive medication use prior to the baseline examination in 1998. However, 311 of the 790 subjects recruited in 1998 reported a previous medical diagnosis of hypertension or had a measured mean resting systolic blood pressure (SBP) ≥ 140 mm Hg or diastolic blood pressure (DBP) ≥ 90 mm Hg [3] and were not excluded from the cohort. Upon our request, Chang et al. reanalyzed the data without these workers (personal communication: 1 August 2024, Table 1). The results of the reanalysis are shown in Figure 1.

Also, despite the independent extraction of data, the prevalence of hypertension in one of the comparison groups examined by Shaykhlislamova et al. [2] was extracted as 10% (n = 24 of 242) instead of 12% (29 of 242). We corrected the prevalence and the corresponding prevalence ratios (PR) for the occupations shown in Table 2.

The corrected values are now included in the meta-analysis with subgroup analyses for high and low risk of bias shown in Figure 1. We found a statistically significantly increased risk of hypertension of 1.68 (95% CI 1.43–1.99) in high-risk studies and a statistically significantly increased risk of hypertension of 1.75 (95% CI 1.06–2.89) in low-risk studies (Figure 1 of this erratum). In Figure 6 of our publication, the comparable results were ES = 1.72 (95% CI 1.45–2.03) in high-risk studies and ES = 1.85 (95% 1.17–2.90) in low-risk studies.

Because of the changes regarding the study by Chang et al., 2013, we also revised Figures 2, 3, 7 and 8 in the publication of the systematic review with only minor deviations from the original publication.

In summary, the reanalysis was necessary due to new findings, which show slight risk reductions. However, the core results of the systematic review with the evidence of a dose–response relationship between occupational noise exposure and the risk of arterial hypertension remain unchanged.

**Figure 1:**
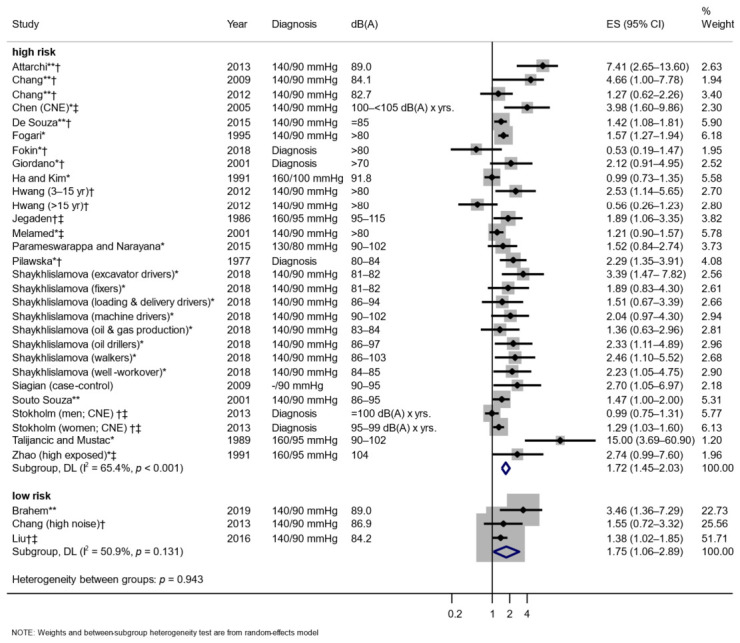
Forest plot of study results stratified by risk of bias with corrected values. Studies marked with * indicate that we calculated the effect size (ES) from the reported prevalence. Studies marked with ** indicate that the odds ratio was corrected to represent the prevalence ratio. † indicates that a physician diagnosis of hypertension was included in the hypertension definition, and ‡ indicates that anti-hypertensive use was included in the hypertension definition. The gray shaded areas around each effect estimate is a visual depiction of the study weight.

## Error in Figure

In the original publication, Figures 2, 3 and 6–8 needed to be corrected to account for a reanalysis of results from the study by Chang et al., 2013. The corrected Figure 2, Figure 3, Figure 6, Figure 7 and Figure 8 appear below. The corresponding citations were also added. 

## Error in Table

In the original publication, Table 2 needed to be corrected to correct the author name of the citation. The corrected Table 2 appears below.
**Study****Study Type****Major Domains****Minor Domains****OVERALL****Recruitment & Follow-up (Cohort)****Exposure Definition & Measurement****Outcome Assessment & Validation****Con-Foun-Ding & Effect Modification****Analysis Method****Chronology****Assessor Blinding****Funding****Conflict of Interest**Attarchi 2013 [48]CS
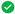

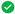

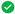

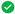

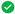

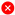

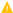

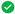

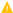

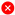
Brahem 2019 [49] CS
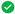

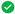

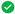

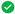

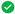

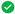

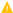

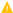

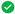

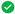
Chang 2009 [44]CS
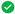

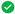

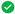

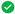

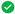

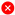

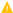

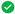

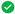

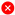
Chang 2012 [50]CS
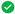

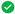

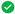

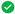

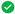

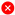

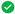

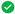

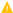

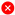
Chang 2013 [51]Co
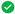

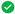

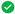

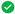

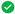

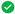

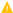

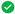

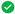

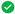
Chen 2005 and Pang 2005 [52,63]CS
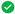

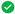

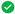

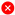

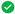

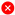

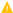

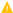

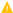

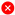
De Souza 2015 [53]CS
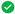

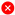

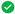

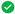

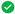

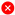

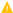

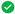

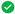

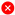
Fogari 1994 [54] §CS
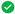

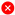

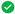

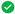

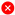

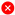

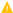

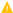

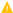

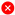
Fogari 1995 [55] CS
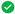

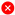

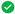

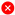

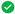

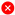

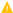

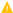

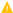

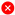
Fokin 2018 [56]Co
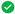

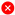

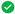

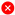

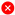

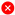

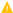

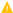

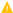

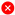
Giordano 2001 [57]CS
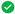

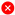

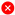

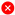

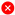

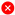

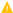

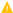

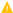

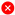
Ha & Kim 1991 [58]CS
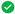

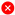

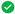

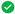

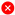

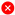

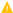

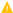

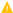

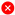
Hwang 2012 [59]Co
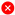

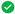

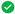

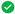

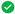

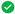

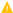

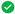

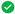

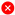
Jegaden 1986 [60]CS
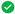

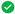

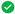

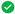

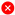

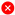

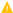

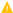

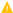

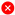
Liu 2016 [61]Co
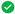

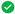

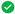

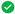

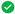

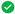

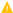

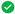

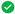

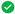
Melamed 2001 [62]Co
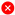

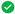

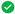

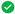

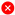

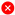

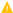

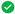

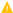

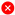
Parameswarappa 2015 [64]CS
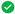

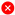

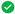

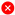

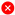

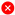

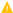

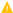

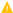

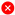
Pilawska 1977 [65]CS
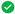

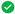

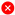

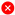

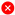

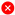

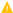

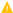

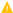

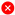
Shaykhlislamova 2018 [66]CS
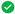

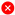

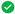

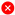

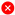

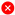

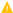

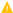

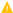

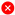
Siagian 2009 [67]Nested CC
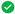

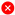

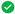

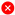

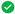

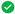

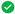

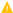

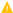

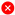
Souto Souza 2001 [68] CS
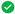

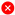

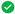

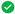

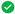

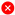

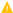

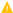

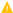

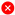
Stokholm 2013 [69]Co
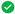

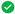

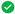

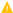

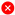

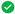

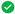

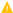

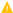

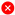
Talijancic 1989 [70]CS
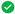

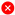

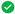

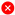

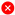

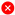

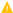

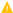

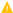

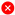
Zhao 1991 and 1993 [71,72]CS
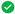

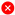

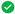

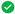

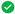

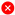

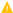

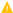

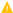

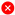
CS Cross-sectional; Co Cohort; CC Case-Control; 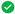 Low Risk; 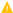 Unclear Risk; 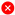 High Risk; § not in meta-analysis.

## Missing Citation

In the original publication, figures’ captions did not contain the reference citations. The citation has now been inserted in captions of Figures 2–4, 6 and 7 and should read:

**Figure 2.** Forest plot for noise exposure >80 dB(A) versus ≤80 dB(A) grouped according to hypertension definitions. Studies marked with * indicate that we calculated the effect size (ES) from the reported prevalences (generally this ES was unadjusted except for job-complexity: Melamed 2001 [62]; age: Ha & Kim 1991 [58], Parameswarappa & Narayana 2015 [64], Giordano 2001 [57]). Studies marked with ** indicate that the odds ratio was corrected to represent the prevalence ratio. † indicates that a physician diagnosis of hypertension was included in hypertension definition, and ‡ indicates that anti-hypertensive use was included in the hypertension definition [44,48–53,55–62,64–70,72].
**Figure 3.** Forest plot for noise exposure in the range of >80 to ≤85 dB(A) and >85 to ≤90 dB(A) using the 140/90 hypertension definition. Studies marked with * indicate that we calculated the effect size (ES) from the reported prevalence. Studies marked with ** indicate that the odds ratio was corrected to represent the prevalence ratio. † indicates that a physician diagnosis of hypertension was included in hypertension definition, and ‡ indicates that anti-hypertensive use was included in the hypertension definition [44,48–51,61,66].
**Figure 4.** Forest plot of risk per 10 dB(A) L_EX,40y_ among studies where the average noise and duration of employment was reported for both the exposure and comparison groups, and where hypertension was defined as blood pressure exceeding 140/90 mmHg. The effect estimates of studies marked with * indicate that effect size (ES) were calculated from the reported prevalence, and studies marked with ** indicate that the odds ratio was corrected to represent the prevalence ratio [44,48,50,61].
**Figure 6.** Forest plot of study results stratified by risk of bias. Studies marked with * indicate that we calculated the effect size (ES) from the reported prevalence. Studies marked with ** indicate that the odds ratio was corrected to represent the prevalence ratio. † indicates that a physician diagnosis of hypertension was included in hypertension definition, and ‡ indicates that anti-hypertensive use was included in the hypertension definition [44,48–53,55–62,64–70,72].
**Figure 7.** Forest plot depicting study results stratified by sex. Studies marked with * indicate that we calculated the effect size (ES) from the reported prevalence. Studies marked with ** indicate that the odds ratio was corrected to represent the prevalence ratio. † indicates that a physician diagnosis of hypertension was included in hypertension definition, and ‡ indicates that anti-hypertensive use was included in the hypertension definition [44,48–53,55–62,64–70,72].

## Text Correction

There was a typographical error in the original publication. This was an error due to rounding in the provided example calculation. A correction has been made to Section 2.5, paragraph 3:

This resulted in an effect estimate of 3.96 per 10 dB(A) L_EX,40y_. 

There was a typographical error in the original publication. A correction has been made to Caption of Figure 4:

**Figure 4.** Forest plot of risk per 10 dB(A) L_EX,40y_ among studies where the average noise and duration of employment was reported for both the exposure and comparison groups, and where hypertension was defined as blood pressure exceeding 140/90 mmHg. 

In the original publication, corrections have been made to Abstract, Section 3.2, paragraph 10, 11, 13, 14, Sections 3.3, 3.4, 4, 4.1 and 4.2 to reflect the reanalysis of results from the study by Chang et al., 2013. 

**Abstract:**

Twenty-four studies were included in the review. The meta-analysis found a pooled effect size (ES) for hypertension (systolic/diastolic blood pressure ≥140/90 mmHg) due to noise exposures ≥80 dB(A) of 1.75 (95% CI 1.46–2.10). 

We found a positive dose-response-relationship: ES = 1.21 (95% CI 0.78–1.87) ≤ 80 dB(A), ES = 1.66 (95% CI 1.24–2.22) > 80–≤85 dB(A), and ES = 3.38 (95% CI 1.37–8.35) > 85–≤90 dB(A). 

**Section 3.2, paragraph 3:**

The resulting pooled risk estimate for occupational noise exposure exceeding 80 dB(A) in this group was 1.75 (95% CI 1.46–2.10). Moderate heterogeneity (I^2^ = 55.2%) was observed, but the heterogeneity was lower in this subgroup as compared to other diagnosis subgroups with two or more studies. 

**Section 3.2, paragraph 6:**

Occupational noise exposure in the >80 to ≤85 dB(A) range corresponded with a pooled risk estimate of 1.66 (95% CI 1.24–2.22, I^2^ = 30.7%). 

**Section 3.2, paragraph 9:**

All three studies with an overall low risk of bias used the 140/90 mmHg definition of hypertension, and their pooled effect estimate was 1.75 (95% CI 1.06–2.89, I^2^ = 50.9%) for occupational noise exposures exceeding 80 dB(A). In comparison, the pooled estimate for studies with an overall high risk of bias was lower (1.68; 95% CI 1.43–1.99), heterogeneity greater (I^2^ = 64.5%), and these studies applied different definitions of hypertension.

**Section 3.2, paragraph 10:**

The stratification according to sex of the study population is shown in Figure 7. This analysis showed comparable risk increases for worker populations including only men (ES = 1.72; 95% CI 1.32–2.22; I^2^ = 64.6%) and those including both men and women (ES = 1.67; 95% CI 1.29–2.16; I^2^ = 69.2%).

**Section 3.2, paragraph 12:**

The combined effect increased when cross-sectional studies were considered separately (ES = 1.97; 95% CI 1.60–2.42, I^2^ = 50.7%). The pooled effect was attenuated for the four cohort studies (ES = 1.29; 95% CI 0.95–1.75, I^2^ = 48.6%).

**Section 3.3, paragraph 1:**

According to this method, the pooled RR (random-effects) would still be significant if the funnel plot were symmetrical (corrected RR = 1.38; 95% CI 1.17–1.63).

**Section 3.4, paragraph 1:**

Thus, we upgraded one level for dose-response gradient. We also upgraded for effect size, because the effect size was >2 in the subgroup of workers exposed to >85–≤90 dB(A) (ES = 3.38; 95% CI 1.37–8.35).

**Section 3.4, table footer of Table 3:**

^2^ While a majority of the studies had a high risk of bias, considering the pooled effect of low risk of bias studies increased the risk estimate (RR 1.75; 95% CI 1.06–2.89); 

^5^ The pooled effect of the main analysis (ES) did not exceed 2.0 (ES = 1.68; 95% CI 1.44–1.96; Figure 2), but ES in the subgroup of workers exposed to >85–≤90 dB(A) was 3.38 (95% CI 1.37–8.35); 

**Section 3.4, paragraph 2:**

For instance, the lower heterogeneity in the subgroup analysis of noise exposures in the range of >80–≤85 dB (I^2^ = 30.7%) indicates that the noise exposure level explains part of the heterogeneity.

**Section 4, paragraph 1:**

The present systematic review identified 24 epidemiological studies and the meta-analysis of 23 of these studies indicates a significantly increased risk of hypertension by a factor of 1.68 (95% CI 1.44–1.96) (Figure 2).

**Section 4.1, paragraph 2:**

However, even in these methodologically sound studies, the risk of hypertension was found to be significantly increased by a factor of 1.75 (95% CI 1.06–2.89) (Figure 6).

**Section 4.1, paragraph 3:**

In the largest group of studies with a definition of 140/90 mmHg, the risk of hypertension was significantly increased by a factor of 1.75 (95% CI 1.46–2.10) in the meta-analysis (Figure 2).

**Section 4.2, paragraph 1:**

For one, employees with an occupational noise exposure of >80–≤85 dB(A) had a significantly increased risk of hypertension by a factor of 1.66 (95% CI 1.24–2.22) in the meta-analysis, whereas this risk was significantly increased by a factor of 3.38 (95% CI 1.37–8.35) for employees with a noise exposure of >85–≤90 dB(A) (Figure 3). 

**Figure 2:**
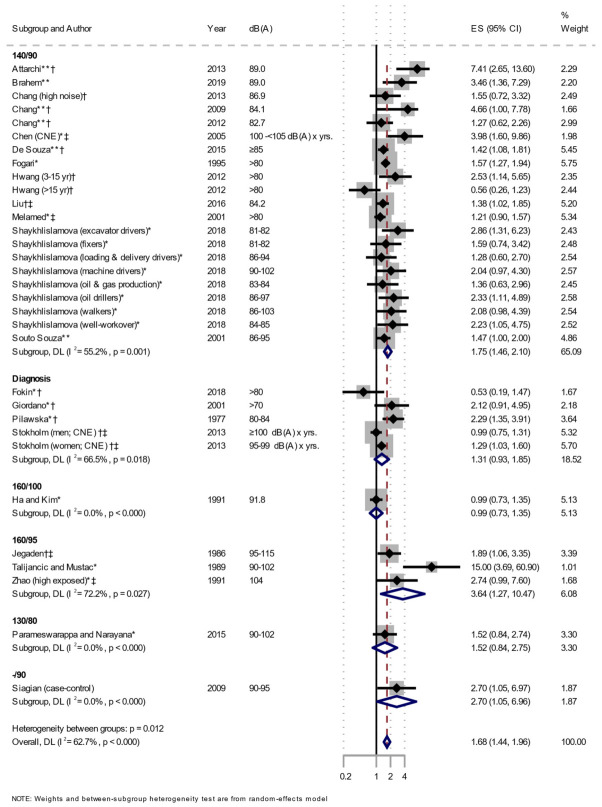
Forest plot for noise exposure >80 dB(A) versus ≤80 dB(A) grouped according to hypertension definitions. Studies marked with * indicate that we calculated the effect size (ES) from the reported prevalences (generally this ES was unadjusted except for job-complexity: Melamed 2001 [62]; age: Ha & Kim 1991 [58], Parameswarappa & Narayana 2015 [64], Giordano 2001 [57]). Studies marked with ** indicate that the odds ratio was corrected to represent the prevalence ratio. † indicates that a physician diagnosis of hypertension was included in hypertension definition, and ‡ indicates that anti-hypertensive use was included in the hypertension definition [44,48–53,55–62,64–70,72].

**Figure 3:**
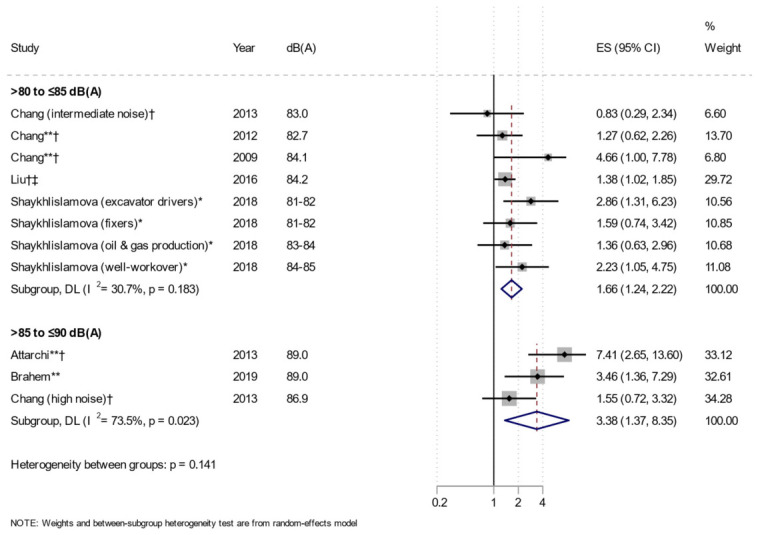
Forest plot for noise exposure in the range of >80 to ≤85 dB(A) and >85 to ≤90 dB(A) using the 140/90 hypertension definition. Studies marked with * indicate that we calculated the effect size (ES) from the reported prevalence. Studies marked with ** indicate that the odds ratio was corrected to represent the prevalence ratio. † indicates that a physician diagnosis of hypertension was included in hypertension definition, and ‡ indicates that anti-hypertensive use was included in the hypertension definition [44,48–51,61,66].

**Figure 6:**
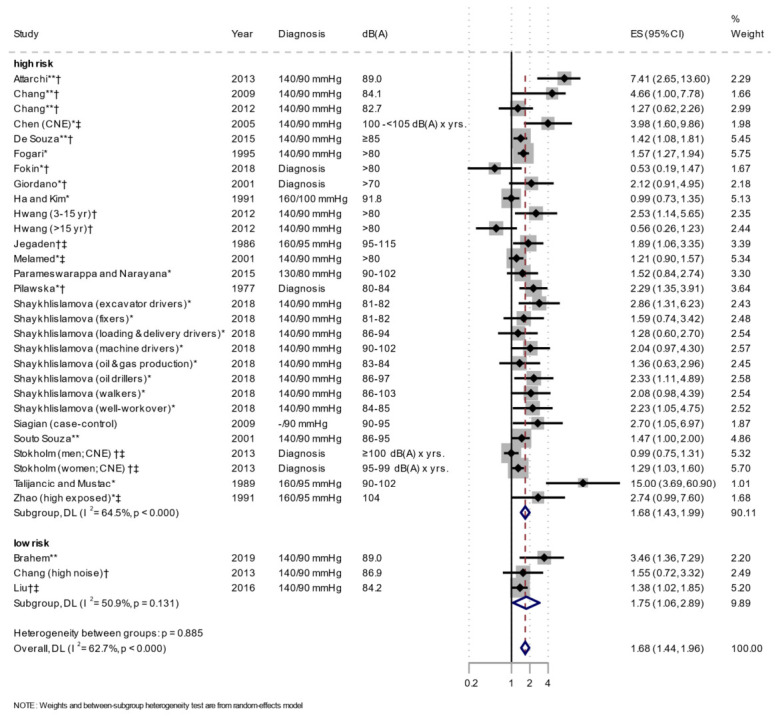
Forest plot of study results stratified by risk of bias. Studies marked with * indicate that we calculated the effect size (ES) from the reported prevalence. Studies marked with ** indicate that the odds ratio was corrected to represent the prevalence ratio. † indicates that a physician diagnosis of hypertension was included in hypertension definition, and ‡ indicates that anti-hypertensive use was included in the hypertension definition [44,48–53,55–62,64–70,72].

**Figure 7:**
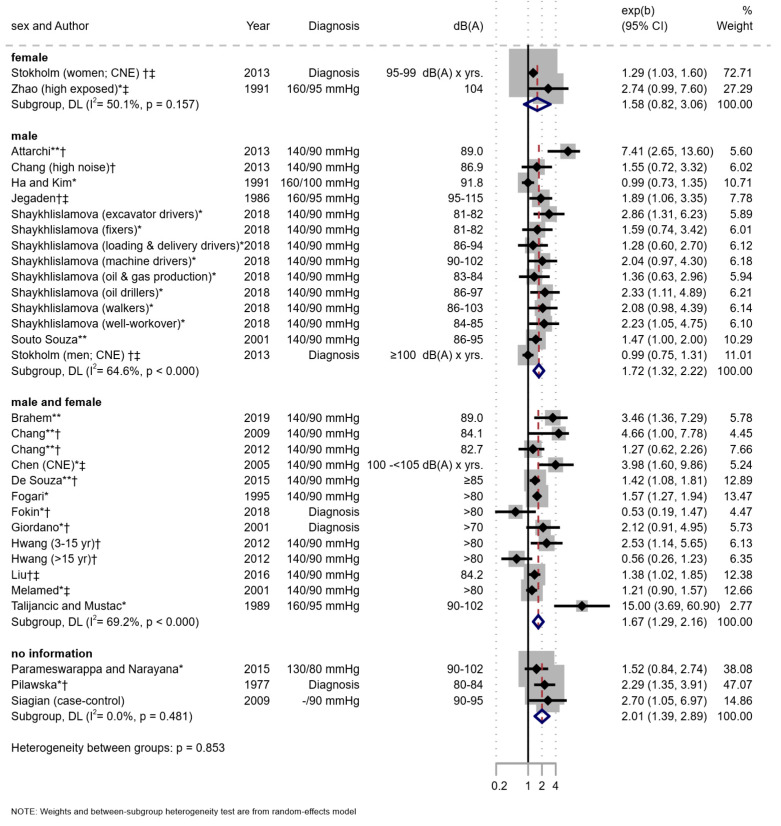
Forest plot depicting study results stratified by sex. Studies marked with * indicate that we calculated the effect size (ES) from the reported prevalence. Studies marked with ** indicate that the odds ratio was corrected to represent the prevalence ratio. † indicates that a physician diagnosis of hypertension was included in hypertension definition, and ‡ indicates that anti-hypertensive use was included in the hypertension definition [44,48–53,55–62,64–70,72].

**Figure 8:**
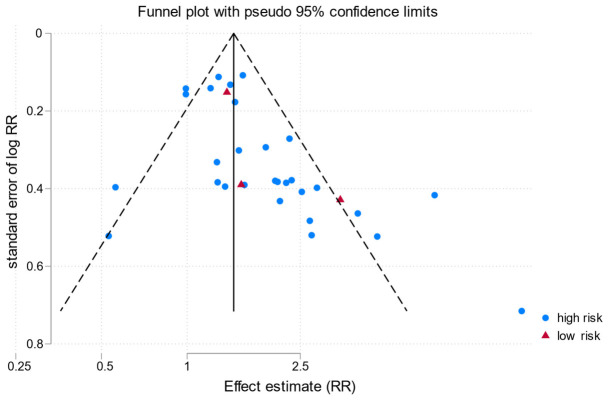
Funnel plot of effect estimates included in the main analysis (Figure 2).

The authors state that the scientific conclusions are unaffected. These corrections were approved by the Academic Editor. The original publication has also been updated.

## Figures and Tables

**Table 1:** The re-analyses for new cases of hypertension limited to the period between 1998 and 2008.

	No. of Cases	Person-Years	Model 2 ^a^		Model 3 ^b^	
			ARR	95% CI	ARR	95% CI
<80	24	2066	1	Reference	1	Reference
80–<85	6	1048	0.89	0.32–2.49	0.83	0.29–2.34
≥85 dBA	21	1815	1.54	0.75–3.18	1.55	0.72–3.32
P_trend_			0.978		0.867	

^a^ The Cox proportional hazards regression adjusted for age and significant factors in simple Cox regression models (such as body mass index and employment duration). ^b^ The Cox proportional hazards regression adjusted for all variables in model 2 and important risk factors reported in the previous literature (i.e., educational level, cigarette use, alcohol intake, and regular exercise) as the final model.

**Table 2:** Corrected self-calculated prevalence ratios (PR) for the ore-mineral mining workers described by Shaykhlislamova et al. [4].

	PR (95% CI)
fixers	1.59 (0.74–3.42)
walkers	2.08 (0.98–4.39)
loading and delivery drivers	1.28 (0.60–2.70)
excavator drivers	2.86 (1.31–6.23)
