# Supplementary material for: Occupational Noise and Hypertension Risk: A Systematic Review and Meta-Analysis
Source: Int J Environ Res Public Health. 2020 Aug 28;17(17):6281. doi: 10.3390/ijerph17176281 (PMC7504405; doi:10.3390/ijerph17176281)
Supplement: Supplementary file 1 [file ijerph-17-06281-s001.pdf]

Supplementary Material

Occupational noise and hypertension risk: a systematic review and meta-analysis

Ulrich Bolm-Audorff, Janice Hegewald, Anna Pretzsch, Alice Freiberg, Albert Nienhaus, Andreas Seidler

List of tables

Table S1. Search String for Medline (via OVID) 2
Table S2. Excluded studies 6
Table S3. Characteristics of included case-control studies 24
Table S4. Results shown in included case-control studies 25
Table S5. Characteristics of included cohort and cross-sectional studies 25
Table S6. Results shown in included cohort and cross-sectional studies 68
Table S7: Risk of bias schema 91
Table S8: Leave-one-out analysis 97

List of figures

Figure S1. Forest plot of study results from studies reporting risks for several exposure levels. 3
Figure S2. Forest plot of lower occupational noise exposure levels. 4
Figure S3. Forest plot of study results from cross-sectional studies versus cohort studies using the 140/90 mmHg hypertension definition. 5

Table S1. Search String for Medline (via OVID)

	**MEDLINE (via Ovid)**
**1.**	exp HYPERTENSION/
**2.**	(hypertension or hypertens$).mp.
**3.**	exp Blood Pressure/
**4.**	(blood pressure or bloodpressure).mp.
**5.**	or/1-4
**6.**	exp NOISE/
**7.**	exp NOISE, OCCUPATIONAL/
**8.**	noise.mp.
**9.**	or/6-8
**10.**	and/5,9

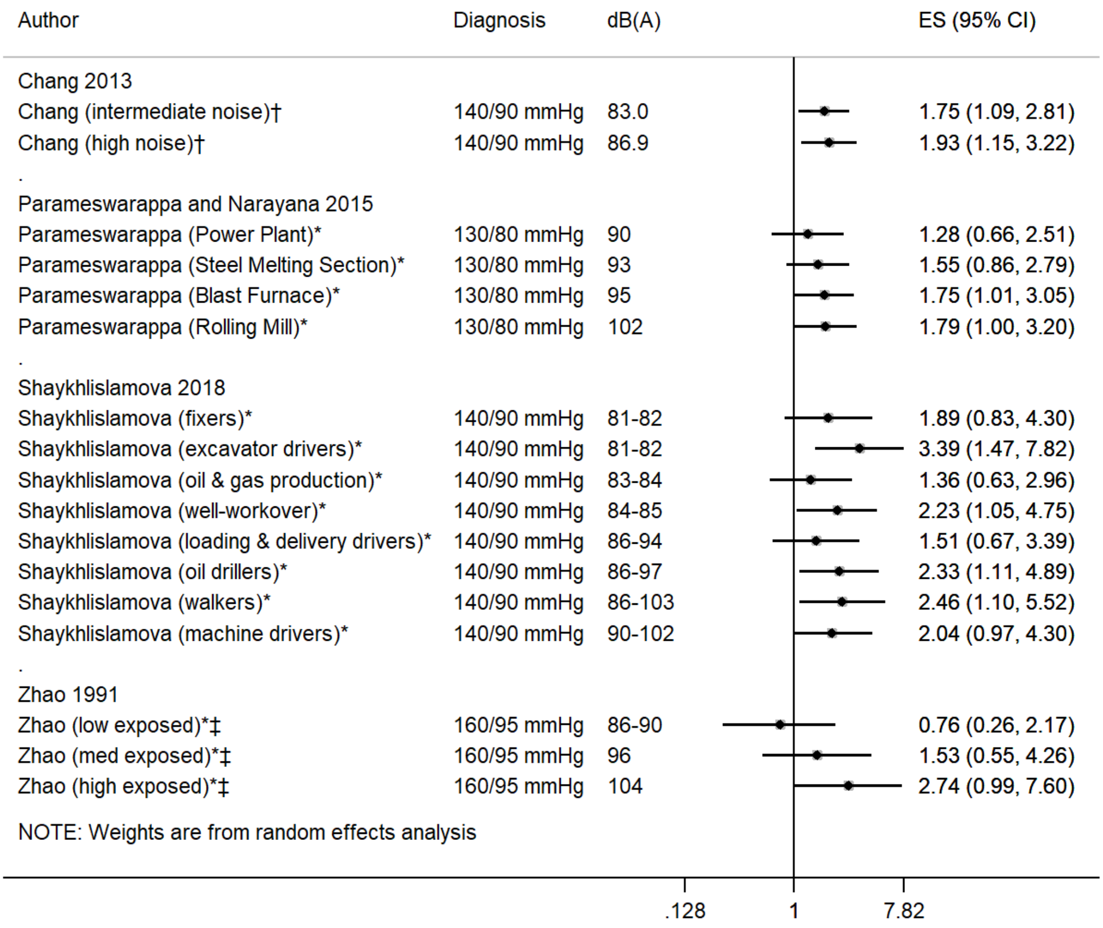

Figure S1. Forest plot of study results from studies reporting risks for several exposure levels.

*Studies marked with * indicate that we calculated the effect size (ES) from the reported prevalence. Studies marked with ** indicate that the odds ratio was corrected to represent the prevalence ratio. † indicates that a physician diagnosis of hypertension was included in hypertension definition, and ‡ indicates that anti-hypertensive use was included in the hypertension definition.*

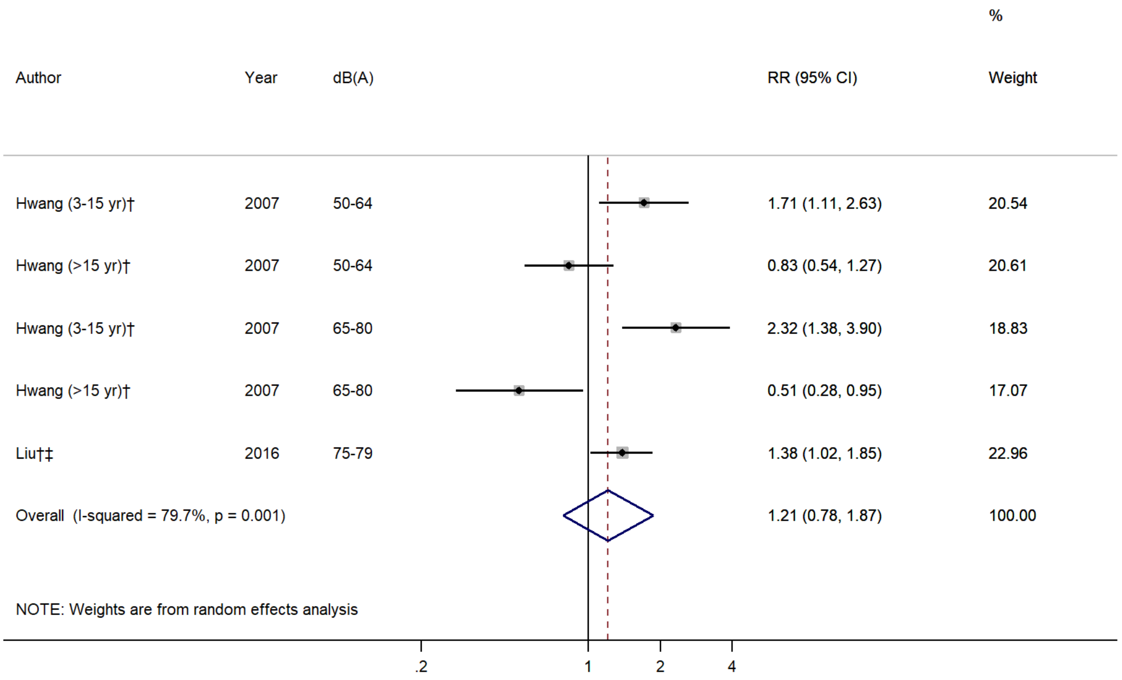

Figure S2. Forest plot of lower occupational noise exposure levels.

The studies marked with † included a self-reported physician-diagnosed hypertension in the outcome assessment. Studies marked with ‡ also considered the use of antihypertensive medication in the outcome assessment.

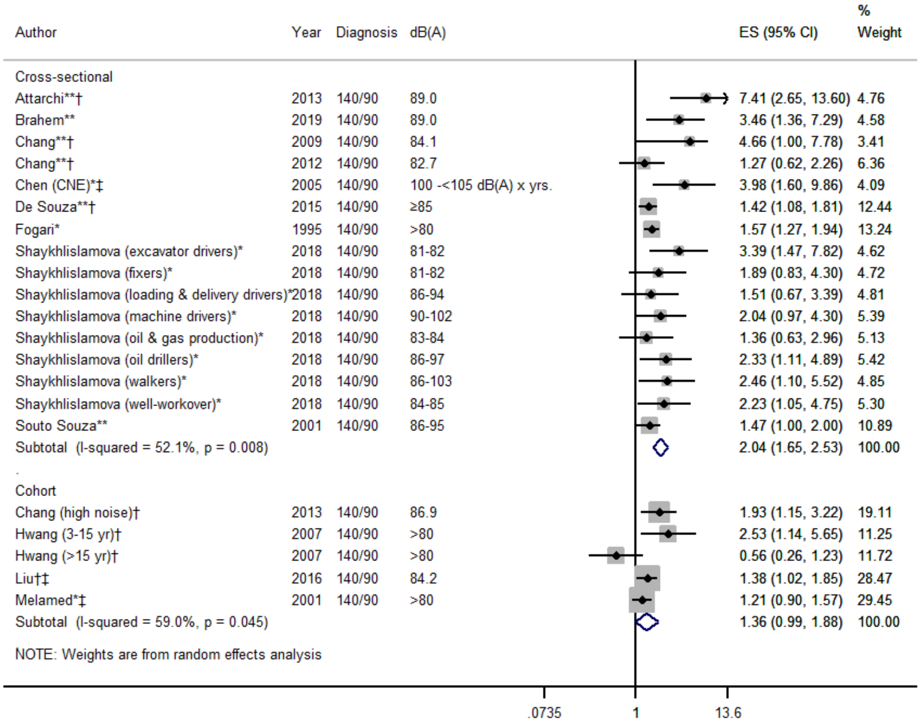

Figure S3. Forest plot of study results from cross-sectional studies versus cohort studies using the 140/90 mmHg hypertension definition.

The studies marked with † included a self-reported physician-diagnosed hypertension in the outcome assessment. Studies marked with ‡ also considered the use of antihypertensive medication in the outcome assessment.

Table S2. Excluded studies

**Reference** (e: exposition; e: comparison; o: outcome; p: population; n: no response given; r: response<10%; s: study design)	**Reason for exclusion**						
	**e**	**c**	**o**	**p**	**n**	**r**	**s**
**Abbate C, Giorgianni C, Munaò F, Costa C, Brecciaroli R, Barbaro M**. 2002. Effects of noise on functional cardiovascular parameters: a follow-up study. Giornale italiano di medicina del lavoro ed ergonomia 24(1):43-48.			❖		❖		
**Afanasova O, Poteriaeva E, Vereshchagina G**. 2010. [Influence of work conditions on the development of arterial hypertension in workers under conditions of high occupational risk]. Meditsina truda i promyshlennaia ekologiia(8):19-22.							
**Andriukin AA**. 1961. The level of arterial pressure and the frequency of hypertension in workers of noisy plants. Gigiena truda i professional'nye zabolevaniia 5:11-17.		❖			❖		
**Andrukovich AI**. 1965. The effect of industrial noise in winding and weaving factories on the arterial pressure of operators. Meditsina Truda I Promyshlennaya Ekologiya 9(12):39-42.					❖		
**Arnold LM, Cappelleri JC, Clair A, Masters ET**. 2013. Interpreting Effect Sizes and Clinical Relevance of Pharmacological Interventions for Fibromyalgia. Pain and Therapy 2(1):65-71.	❖			❖			❖
**Aro S**. 1984. Occupational stress, health-related behavior, and blood pressure: a 5-year follow-up. Prev Med 13(4):333-48.			❖		❖		
**Assunta C, Ilaria S, Simone DS, Gianfranco T, Teodorico C, Carmina S, Anastasia S, Roberto G, Francesco T, Valeria RM**. 2015. Noise and cardiovascular effects in workers of the sanitary fixtures industry. International Journal of Hygiene and Environmental Health 218(1):163-168.					❖		
**Attarchi M, Dehghan F, Safakhah F, Nojomi M, Mohammadi S.** 2012. Effect of exposure to occupational noise and shift working on blood pressure in rubber manufacturing company workers. Industrial Health 50(3):205-213.		❖					

Reference (e: exposition; e: comparison; o: outcome; p: population; n: no response given; r: response<10%; s: study design)	Reason for exclusion						
	e	c	o	p	n	r	s
**Bagheri Hosseinabadi M, Khanjani N, Münzel T, Daiber A, Yaghmorloo M**. 2019. Chronic occupational noise exposure: Effects on DNA damage, blood pressure, and serum biochemistry. Mutation Research - Genetic Toxicology and Environmental Mutagenesis 841:17-22.			❖				
**Balaji R, Rajasegaran R, John NA, Venkatappa US.** 2016. Hearing impairment and high blood pressure among bus drivers in puducherry. Journal of Clinical and Diagnostic Research 10(2):CC08-CC10.			❖		❖		
**Belli S, Sani L, Scarficcia G, Sorrentino R**. 1984. Arterial hypertension and noise: a cross-sectional study. American Journal of Industrial Medicine 6(1):59-65.		❖					
**Bornand E, Lob M**. 1975. Influence of occupational activities upon health: study of a group of roadmen working on highways and another group working on main and secondary roads. Archives des Maladies Professionnelles de Medecine du Travail et de Securite Sociale 36(7-8):385-395.					❖		
**Britanov NG**. 1979. Effect of Noise and Acetone on Female Workers of Acetate and Polyvinyl Chloride Fiber Factories. Meditsina Truda I Promyshlennaya Ekologiya(12):15-19.					❖		
**Brown JE, Thompson RN, Folk ED**. 1975. Certain non-auditory physiological responses to noises. Am Ind Hyg Assoc J 36(4):285-91.			❖				
**Capellini A, Maroni M**. 1974. Clinical survey on hypertension and coronary disease and their possible relations with the environment in workers of a chemical plant. [Italian]. Medicina del Lavoro 65(7-8):297-305.		❖					
**Cattin L, Da Col PG, Zotti E**. 1979. Survey of cardiovascular risk factors in a population of shipyard workers. [Italian]. Giornale della Arteriosclerosi 4(2):115-126.			❖[1]				

[1] no information about the relationship between noise exposure and hypertension

**Reference** (e: exposition; e: comparison; o: outcome; p: population; n: no response given; r: response<10%; s: study design)	**Reason for exclusion**						
	**e**	**c**	**o**	**p**	**n**	**r**	**s**
**Cayir A, Barrow TM, Wang H, Liu H, Li C, Ding N, Li Y, Kang CM, Guo L, Li PH, Byun HM**. 2018. Occupational noise exposure is associated with hypertension in China: Results from project ELEFANT. PLoS ONE 13(12).	❖				❖		
**Chang TY, Jain RM, Wang CS, Chan CC**. 2003. Effects of Occupational Noise Exposure on Blood Pressure. Journal of Occupational and Environmental Medicine 45(12):1289-1296.			❖		❖		
**Chang TY, Liu CS, Bao BY, Li SF, Chen TI, Lin YJ**. 2011. Characterization of road traffic noise exposure and prevalence of hypertension in central Taiwan. Science of the Total Environment 409(6):1053-1057.	❖						
**Chang TY, Liu CS, Huang KH, Chen RY, Lai JS, Bao BY**. 2011. High-frequency hearing loss, occupational noise exposure and hypertension: A cross-sectional study in male workers. Environmental Health: A Global Access Science Source 10(1).	❖						
**Chang TY, Liu CS, Hwang BF, Hsieh HH, Bao BY, Chen CJ, Wang VS, Lai JS**. 2015. Acute effects of noise exposure on 24-h ambulatory blood pressure in hypertensive adults. Journal of Hypertension 33(3):507-514.						❖	
**Chang TY, Su TC, Lin SY, Jain RM, Chan CC**. 2007. Effects of occupational noise exposure on 24-hour ambulatory vascular properties in male workers. Environ Health Perspect 115(11):1660-4.			❖				
**Chang TY, Wang H, Liu CS, Sieh H, Bao BY, Lai JS**. 2013. Acute effects of occupational noise exposure on 24-hour ambulatory blood pressure in workers with hypertension. Occupational and Environmental Medicine. Conference: 23rd Conference on Epidemiology in Occupational Health, EPICOH 70(SUPPL. 1).							❖
Chang TY, Wang VS, Lin SY, Yen HY, Lai JS, Liu CS. 2010. Co-exposure to noise, N,N-dimethylformamide, and toluene on 24-hour ambulatory blood pressure in synthetic leather workers. Journal of Occupational and Environmental Hygiene 7(1):14-22.			❖				
**Chen SC, Ni YQ, Zhang L, Kong LY, Lu LY, Yang ZP, Yang LX, Zhang XH, Zhu YM**. 2017. Noise exposure in occupational setting associated with elevated blood pressure in China. Bmc Public Health 17:7.					❖		

Reference (e: exposition; e: comparison; o: outcome; p: population; n: no response given; r: response<10%; s: study design)	Reason for exclusion						
	e	c	o	p	n	r	s
**Chen Z**. 2007. Analysis of Noise Influence in Hearing and Cardiovascular System of Industrial Worker. Practical Preventive Medicine 14(3):766-767.					❖		
**Chen Z, Nong W, Liu D, Ling W**. 2003. [Analysis of the correlation between cumulative noise exposure and cardiovascular diseases]. Chin Occup Med. 30(6):51.					❖		
**Delin CO**. 1984. Noisy work and hypertension. Lancet 2(8408):931.					❖		❖
**Deyanov C, Mincheva L, Hadjiolova I, Ivanovich E**. 1995. Study on the level of blood pressure and prevalence of arterial hypertension depending on the duration of occupational exposure to industrial noise. C E J Occup Environ Med 1:109-116.	❖				❖		
**Doyon B, Debru JL, Perdrix A**. 1978. Effects of noise on blood pressure. Coeur et Medecine Interne 17(suppl.):61-65.			❖		❖		
**Duclos J, Chaurand A**. 1987. Du traumatisme sonore chronique et de l'hypertension arterielle. Archives des maladies professionnelles et de medecine du travail; Organe officiel des Societes de Medecine du Travail de Paris, Lilie et Lyon 48:151-154.		❖			❖		
**Falian AB, Kusnoputranto H**. 2018. Relationship analysis of noise to hypertension on workers at pharmaceutical products factory X in 2018, Depok City, West Java Province. Indian Journal of Public Health Research and Development 9(11):358-364.		❖			❖		
**Fernández-D'Pool J, Butrón J, Colina-Chourio J**. 2010. Effect of noise on blood pressure in workers of a Venezuelan oil company. Investigacion Clinica 51(3):301-314.		❖					
**Fogari R, Zoppi A, Corradi L, Marasi G, Vanasia A, Zanchetti A**. 2001. Transient but not sustained blood pressure increments by occupational noise. An ambulatory blood pressure measurement study. Journal of Hypertension 19(6):1021-1027.			❖				

Reference	(e: exposition; e: comparison; o: outcome; p: population; n: no response given; r: response<10%; s: study design)	Reason for exclusion						
		e	c	o	p	n	r	s
**Fogari R, Zoppi A, Lusardi P, Malamani G, Marasi G, Villa G, Vanasia A**. 1995. Noise-induced hearing loss and blood pressure in a worker population: A cross-sectional study. High Blood Press. 4:182-185.			❖			❖		
**Fu X, Yang E, Duan Z, Zhang C, Yu H, Zheng G**. 2011. Study on the effect of occupational noise exposure and color doppler sonography indexes to cardiovascular system. Chin Occup Med. 38(5).						❖		
**Gan WQ, Davies HW, Demers PA**. 2011. Exposure to occupational noise and cardiovascular disease in the United States: The National Health and Nutrition Examination Survey 1999-2004. Occupational and Environmental Medicine 68(3):183-190.		❖						
**Gan WQ, Moline J, Kim H, Mannino DM**. 2016. Exposure to loud noise, bilateral high-frequency hearing loss and coronary heart disease. Occupational and Environmental Medicine 73(1):34-41.		❖		❖				
**Garcia AM, Garcia A**. 1993. Occupational noise as a cardiovascular risk factor. Schriftenreihe des Vereins fur Wasser-, Boden- und Lufthygiene 88:212-222.[2]		❖				❖		
**García AM, García A**. 1992. Relationship between arterial pressure and exposure to noise at work. Medicina clínica 98(1):5-8.[2]		❖				❖		
**Geller LI, Sakaeva SZ, Musina SS, Kogan ID, Belomyttseva LA, Ostrovskaia RS, Volokhov IP, Lukianova ES, Popova RM, Moskatelnikova EV**. 1963. [on the Effect of Noise on Arterial Pressure (on the Problem of the Etiology of Hypertension)]. Ter Arkh 35:83-6.						❖		
**Graff C, Bockmüphl F, Tietze V**. 1968. Lärmbelastung und arterielle (essentielle) Hypertoniekrankheit beim Menschen. In: Nitschkoff S, Kriwizkaja G, editors. Lärmbelastung, akustischer Reiz und neurovegetative Störungen. Georg-Thieme Verlag. S. 112-126.						❖		

[2] Both publications describe the same study.

**Reference**	(e: exposition; e: comparison; o: outcome; p: population; n: no response given; r: response<10%; s: study design)	**Reason for exclusion**						
		**e**	**c**	**o**	**p**	**n**	**r**	**s**
**Green MS, Schwartz K, Harari G, Najenson T**. 1991. Industrial noise exposure and ambulatory blood pressure and heart rate. Journal of Occupational Medicine 33(8):879-883.			❖	❖				
**Gupta S, Malhotra V, Tripathi Y, Dev P**. 2017. Blood pressure variations in textile mill middle-aged male workers exposed to noise. National Journal of Physiology, Pharmacy and Pharmacology 7(5):491-496.							❖	
**Hammoudi N, Aoudi S, Tizi M, Larbi K, Bougherbal R**. 2013. Relationship between noise and blood pressure in an airport environment. [French]. Annales de Cardiologie et d'Angeiologie 62(3):166-171.			❖			❖		
**Hedstrand H, Drettner B, Klockhoff I, Svedberg A**. 1977. Noise and blood-pressure. Lancet 2(8051):1291.		❖				❖		❖
**Hessel PA, Sluiscremer GK**. 1994. Occupational noise exposure and blodd pressure - longitudinal and cross-sectional observations in a groupof underground miners. Archives of Environmental Health 49(2):128-134.				❖				
**Hirai A, Takata M, Mikawa M, Yasumoto K, Iida H, Sasayama S, Kagamimori S**. 1991. Prolonged exposure to industrial noise causes hearing loss but not high blood pressure: A study of 2124 factory laborers in Japan. Journal of Hypertension 9(11):1069-1073.						❖		
**Huo Yung Kai S, Ruidavets JB, Carles C, Marquie JC, Bongard V, Leger D, Ferrieres J, Esquirol Y**. 2018. Impact of occupational environmental stressors on blood pressure changes and on incident cases of hypertension: a 5-year follow-up from the VISAT study. Environmental health : a global access science source 17(1):79.		❖						
**Hwang WJ, Hong O**. 2014. Impact of Noise Exposure on Hypertension. Global Heart 1):e133.								❖
**Idzior-Waluś B**. 1987. Coronary risk factors in men occupationally exposed to vibration and noise. European Heart Journal 8(7):1040-1046.			❖			❖		

Reference	(e: exposition; e: comparison; o: outcome; p: population; n: no response given; r: response<10%; s: study design) Reason for exclusion						
	e	c	o	p	n	r	s
**Inoue M, Laskar MS, Harada N**. 2005. Cross-sectional study on occupational noise and hypertension in the workplace. Archives of Environmental and Occupational Health 60(2):106-110.					❖		
**Ising H, Günther T, Handrock M**. 1981. Noise effects and mineral metabolism. Zeitschrift fur Larmbekampfung 28(6):176-185.							❖
**Ising H, Günther T, Melchert HU**. 1980. [Demonstration and mechanism of blood pressure elevation due to occupational noise]. Zentralbl Arbeitsmed Arbeitsschutz Prophyl Ergonomie 30(6):194-203.		❖			❖		
**Johsson A, Hansson L**. 1977. Prolonged exposure to a stressful stimulus (noise) as a cause of raised blood-pressure in man. Lancet 1(8002):86-7.	❖						
**Jovanovic J, Jovanovic M**. 2004. [Occupational stress and arterial hypertension]. Medicinski Pregled 57(3-4):153-8.	❖				❖		
**Kalicinski A, Straczkowski W, Nowak W, Proniewska W, Rozanska L**. 1975. [Cardiovascular changes in workers exposed to noise]. Wiadomosci Lekarskie 28(1):1-4.		❖			❖		
**Kanevskaia Zh S, Maksimova LI, Kublanova PS, Shevyreva NA, Sineva EL**. 1977. [Effect of pulsating and stable noise on the central nervous systems of workers]. Gig Tr Prof Zabol(1):22-5.		❖	❖		❖		
**Kavoussi N**. 1973. The relationship between the length of exposure to noise and the incidence of hypertension at a silo in Teheran. Medicina del Lavoro 64(7-8):292-295.		❖			❖		
**Kerns E, Masterson EA, Themann CL, Calvert GM**. 2018. Cardiovascular conditions, hearing difficulty, and occupational noise exposure within US industries and occupations. American Journal of Industrial Medicine 61(6):477-491.	❖	❖					
**Kim C-B, Kim J-Y, Cha B-S, Choi H-R, Lee J-T, Nam CM, Lee SY, Wang S-J, Park K, Kim D-Y, Koh S-B**. 2000. A Meta-analysis on the Association between Chronic Noise Exposure and Blood Pressure. Korean J Prev Med 33(3):343-348.							❖

Reference (e: exposition; e: comparison; o: outcome; p: population; n: no response given; r: response<10%; s: study design)	Reason for exclusion						
	**e**	**c**	**o**	**p**	**n**	**r**	**s**
**Kochanova EM, Vermel AE, Papoian SS, Shkarinov LN, Shirokov AI**. 1985. Effect of industrial noise on the prevalence of arterial hypertension. Terapevticheskii Arkhiv 57(4):125-128.					❖		
**Kontosic I, Vukelic M, Grubisic-Greblo H**. 1990. Noise as a risk factor of arterial hypertension in seamen. [Serbian]. Arhiv za Higijenu Rada i Toksikologiju 41(2):187-199.					❖[3]		
**Kornhuber HH, Lisson G**. 1981. Hypertension: Are industrial stress, noise or piece work important factors?. [German]. Deutsche Medizinische Wochenschrift 106(51-52):1733-1736.		❖[4]			❖		
**Korotkov J, Varenikov I, Volkov A, Zaborski L, Szczepański C**. 1985. The noise and functional disturbances of the cardiovascular system in seamen. Bulletin of the Institute of Maritime and Tropical Medicine in Gdynia 36(1-4):29-35.					❖		
**Kotseva K**. 1997. Prevalence of Arterial Hypertension in Electric Motor Production Workers. Central European Journal of Occupational and Environmental Medicine 3(3):224-230.					❖		
**Kristal-Boneh E, Melamed S, Harari G, Green MS**. 1995. Acute and chronic effects of noise exposure on blood pressure and heart rate among industrial employees: The cordis study. Archives of Environmental Health 50(4):298-304.			❖				
**Kwitko A, Pezzi RG, Da Silveira MS**. 1996. Exposure to occupational noise and blood pressure. Revista Brasileira de Otorrinolaringologia 62(2):89-98.		❖			❖		
**Lahoz Zamarro MT, Abenia Ingalaturre JM, Vallés Varela H, Rubio Calvo E**. 1993. Interaction of arterial blood pressure and industrial noise on human hearing. Acta otorrinolaringológica española 44(1):11-16.		❖			❖		
**Lang T, Fouriaud C, Degoulet P**. 1986. Occupational exposure to noise, hearing loss and arterial hypertension. [French]. Revue d'epidemiologie et de sante publique 34(4-5):318-323.		❖					

[3] The response rate is only given for the exposed, not for the control group.

[4] control group exposed to <80dB, but no information concerning the prevalence of hypertension in the control given

Reference	(e: exposition; e: comparison; o: outcome; p: population; n: no response given; r: response<10%; s: study design) Reason for exclusion						
	e	c	o	p	n	r	s
**Lang T, Fouriaud C, Jacquinet-Salord MC**. 1992. Length of occupational noise exposure and blood pressure. International Archives of Occupational and Environmental Health 63(6):369-372.		❖					
**Lee JH, Kang W, Yaang SR, Choy N, Lee CR**. 2009. Cohort Study for the Effect of Chronic Noise Exposure on Blood Pressure Among Male Workers in Busan, Korea. American Journal of Industrial Medicine 52(6):509-517.			❖				
**Lees RE, Roberts JH**. 1979. Noise-induced hearing loss and blood pressure. Can Med Assoc J 120(9):1082-4.		❖			❖		
**Lees RE, Romeril CS, Wetherall LD**. 1980. A study of stress indicators in workers exposed to industrial noise. Can J Public Health 71(4):261-5.		❖					
**Li X, Dong Q, Wang B, Song H, Wang S, Zhu B**. 2019. The Influence of Occupational Noise Exposure on Cardiovascular and Hearing Conditions among Industrial Workers. Sci Rep 9(1):11524.		❖					
**Li Y, Chen G, Yu S**. 2015. Prevalence and influence factors of hypertension among the workers exposed to noise in steel making and steel rolling workshop of an iron and steel plant. Zhonghua yu fang yi xue za zhi [Chinese journal of preventive medicine] 49(5):405-410.		❖					
**Liu J, Xu M, Ding L, Zhang H, Pan L, Liu Q, Ding E, Zhao Q, Wang B, Han L, Yang D, Zhu B**. 2016. Prevalence of hypertension and noise-induced hearing loss in Chinese coal miners. Journal of Thoracic Disease 8(3):422-429.		❖			❖		
**Lusk SL, Hagerty BM, Gillespie B, Caruso CC**. 2002. Chronic Effects of Workplace Noise on Blood Pressure and Heart Rate. Archives of Environmental Health 57(4):273-281.		❖	❖[5]		❖		
**Malchaire JB, Mullier M**. 1979. Occupational exposure to noise and hypertension: a retrospective study. Ann Occup Hyg 22(1):63-6.					❖		

[5] information concerning noise exposure is given, but no information on the correlation between noise exposure and hypertension

**Reference** (e: exposition; e: comparison; o: outcome; p: population; n: no response given; r: response<10%; s: study design)	**Reason for exclusion**						
	**e**	**c**	**o**	**p**	**n**	**r**	**s**
**Manninen O, Aro S**. 1979. Noise-induced hearing loss and blood pressure. Int Arch Occup Environ Health 42(3-4):251-6.	❖		❖			❖	
**Marcellini L, Rosati MV, Ciarrocca M, Ursini A, Tomao E, Tomei F**. 2003. Cardiovascular effects in farmers exposed to noise. [Italian]. Giornale Italiano di Medicina del Lavoro ed Ergonomia 25(SUPPL. 3):229-230.			❖				
**Meinhart P, Renker U**. 1970. [Studies on the morbidity in heart and circulatory diseases with lasting noise exposition]. Z Gesamte Hyg 16(11):853-7.	❖				❖		
**Milković-Kraus S**. 1990. Noise-induced hearing loss and blood pressure. International Archives of Occupational and Environmental Health 62(3):259-260.			❖		❖		
**Narlawar UW, Surjuse BG, Thakre SS**. 2006. Hypertension and hearing impairment in workers of iron and steel industry. Indian Journal of Physiology and Pharmacology 50(1):60-66.			❖				
**Nawaz SK, Hasnain S**. 2010. Noise induced hypertension and prehypertension in Pakistan. Bosnian Journal of Basic Medical Sciences 10(3):239-244.					❖		
**Nawaz SK, Hasnain S**. 2011. Association of ACE ID and ACE G2350A polymorphism with increased blood pressure in persons exposed to different sound levels in Pakistan. International Archives of Occupational and Environmental Health 84(4):355-360.			❖		❖		
**Nawaz SK, Hasnain S**. 2011. Effect of ACE polymorphisms on the association between noise and hypertension in a Pakistani population. JRAAS - Journal of the Renin-Angiotensin-Aldosterone System 12(4):516-520.					❖		
**Ngombe LK, Cowgill K, Monga BB, Ilunga BK, Stanis WO, Numbi OL**. 2015. [Prevalence of hypertension in the population of the millers of the city of Lubumbashi, Democratic Republic of Congo]. Pan Afr Med J 22:152.	❖				❖		

Reference	(e: exposition; e: comparison; o: outcome; p: population; n: no response given; r: response<10%; s: study design)	Reason for exclusion						
		e	c	o	p	n	r	s
**Ni CH, Chen ZY, Zhou Y, Zhou JW, Pan JJ, Liu N, Wang J, Liang CK, Zhang ZZ, Zhang YJ**. 2007. Associations of blood pressure and arterial compliance with occupational noise exposure in female workers of textile mill. Chin Med J (Engl) 120(15):1309-13.			❖			❖		
**Nicolle-Mir L**. 2013. Co-exposure to noise and organic solvents: Effect on arterial pressure. Environnement, Risques et Sante 12(6):470-471.								❖
**Nosov AE, Baydina AS, Ivashova YA, Vlasova EM, Alekseev VB**. 2017. Features of hypertension in workers of titanium and magnesium production. Gigiena i Sanitariya 96(1):62-65.		❖				❖		
**Nserat S, Al-Musa A, Khader YS, Abu Slaih A, Iblan I**. 2017. Blood pressure of jordanian workers chronically exposed to noise in industrial plants. International Journal of Occupational and Environmental Medicine 8(4):217-223.			❖			❖		
**Parvizpoor D**. 1976. Noise exposure and prevalence of high blood-pressure among weavers in Iran. Journal of Occupational and Environmental Medicine 18(11):730-731.			❖			❖		
**Powazka E, Pawlas K, Zahorska-Markiewicz B, Zejda JE**. 2002. A cross-sectional study of occupational noise exposure and blood pressure in steelworkers. Noise and Health 5(17):15-22.			❖			❖		
**Raffi GB, Cavalleri A, Marinelli M**. 1980. Epidemiologic study on correlation between industrial noise and hypertension. Giornale Italiano di Medicina del Lavoro 2(1):7-10.								
**Rapisarda V, Ledda C, Ferrante M, Fiore M, Cocuzza S, Bracci M, Fenga C**. 2016. Blood pressure and occupational exposure to noise and lead (Pb): A cross-sectional study. Toxicol Ind Health 32(10):1729-36.			❖	❖		❖		
**Rizi HAY, Dehghan H**. 2012. Effects of occupational noise exposure on changes in blood pressure of workers. Arya Atherosclerosis 8:S183-S186.			❖	❖		❖		

Reference (e: exposition; e: comparison; o: outcome; p: population; n: no response given; r: response<10%; s: study design)	Reason for exclusion						
	e	c	o	p	n	r	s
**Saad MM, Hussein MS, Hammam HM**. 1994. Study of noise, hearing impairment and hypertension in Egypt. Annals of Saudi Medicine 14(4):307-311.					❖		
**Saha S, Gandhi A, Das S, Kaur P, Singh SH**. 1996. Effect of noise stress on some cardiovascular parameters and audiovisual reaction time. Indian Journal of Physiology and Pharmacology 40(1):35-40.					❖		
**Sancini A, Caciari T, Rosati MV, Samperi I, Iannattone G, Massimi R, Loreti B, Scala B, Sacco C, Tomei F, Tomei G**. 2014. Can noise cause high blood pressure? Occupational risk in paper industry. Clinica Terapeutica 165(4):e304-e311.					❖		
**Sancini A, Tomei G, Vitarelli A, Caciari T, Samperi I, Pacchiarotti A, Scala B, Schifano MP, Scimitto L, Fiaschetti M, Cetica C, Tomei F, Ciarrocca M**. 2012. Cardiovascular risk in rotogravure industry. Journal of Occupational and Environmental Medicine 54(5):551-557.					❖		
**Santana VS, Barberino JL**. 1995. Occupational noise exposure and hypertension. Revista de saúde pública 29(6):478-487.	❖						
**Sbihi H, Davies HW, Demers PA**. Hypertensive disease in sawmill workers chronically exposed to high noise levels; 2006. p 4128-4133.		❖					
**Sbihi H, Davies HW, Demers PA**. 2008. Hypertension in noise-exposed sawmill workers: A cohort study. Occupational and Environmental Medicine 65(9):643-646.		❖					
**Shatalov NN, Murov MA**. 1970. Effect of intensive noise and neuro-psychic tension on arterial blood pressure levels and frequency of hypertensive disease. Klinicheskaya Meditsina 48(3):70-73.					❖		
**Shepelin OP**. 1959. [On the problem of the effect of impulse noise on workers in industrial conditions]. Gig Sanit 24:26-32.			❖		❖		
**Siagian M**. 2012. Hypertension in Indonesian air force pilots. Medical Journal of Indonesia 21(1):38-43.	❖						

Reference	(e: exposition; e: comparison; o: outcome; p: population; n: no response given; r: response<10%; s: study design)	Reason for exclusion						
		e	c	o	p	n	r	s
**Singh AP, Rai RM, Bhatia MR, Nayar HS**. 1982. Effect of chronic and acute exposure to noise on physiological functions in man. International Archives of Occupational and Environmental Health 50(2):169-174.		❖		❖		❖		
**Singhal S, Yadav B, Hashmi SF, Muzammil M**. 2009. Effects of workplace noise on blood pressure and heart rate. Biomedical Research 20(2):122-126.				❖		❖		
**Starzyński Z, Wilczyńska U, Kubasiewicz M, Szymczak W**. 1985. Incidence of arterial hypertension in the population of workers in the textile industry in Lódź. Medycyna pracy 36(2):131-138.			❖					
**Stokholm ZA, Christensen KL, Frederiksen TW, Vestergaard JM, Hansen AM, Bonde JP, Kristiansen J, Luund SP, Buttenschon H, Kolstad HA**. 2013. Does full-shift occupational noise exposure have acute or sustained effects on blood pressure? A 24-hour observational study of industrial workers. European Journal of Epidemiology 1):S58.								❖
**Suvorov GA, Denisov EI, Ovakimov VG, Tavtin YK**. 1979. Correlations Between Hearing Losses and Neurovascular Impairments in Workers in Relation to the Level of Noise. Meditsina Truda I Promyshlennaya Ekologiya(7):18-22.						❖		
**Takala J, Varke S, Vaheri E, Sievers K**. 1977. Noise and blood-pressure. Lancet 2(8045):974-5.		❖						
**Talbott E, Helmkamp J, Matthews K**. 1985. Occupational noise exposure, noise-induced hearing loss, and the epidemiology of high blood pressure. American Journal of Epidemiology 121(4):501-514.			❖					
**Talbott EO, Findlay RC, Kuller LH, Lenkner LA, Matthews KA, Day RD, Ishii EK**. 1990. Noise-induced hearing loss: a possible marker for high blood pressure in older noise-exposed populations. J Occup Med 32(8):690-7.			❖					
**Talbott EO, Gibson LB, Burks A, Engberg R, McHugh KP**. 1999. Evidence for a dose-response relationship between occupational noise and blood pressure. Arch Environ Health 54(2):71-8.			❖					

Reference (e: exposition; e: comparison; o: outcome; p: population; n: no response given; r: response<10%; s: study design)	Reason for exclusion						
	**e**	**c**	**o**	**p**	**n**	**r**	**s**
**Taleb A, Mohammed Brahim B, Benrezkallah L, Mahi Benkalfat FZ**. 2003. Noise exposure, psychosocial environment and arterial hypertension in industrial companies. Archives des Maladies Professionnelles et de Medecine du Travail 64(4):246-252.	❖				❖		
**Tarter SK, Robins TG**. 1990. Chronic noise exposure, high-frequency hearing loss, and hypertension among automotive assembly workers. J Occup Med 32(8):685-9.		❖					
**Tessier-Sherman B, Galusha D, Cantley LF, Cullen MR, Rabinowitz PM, Neitzel RL**. 2017. Occupational noise exposure and risk of hypertension in an industrial workforce. American Journal of Industrial Medicine 60(12):1031-1038.		❖					
**Tiwai RR, Pathak MC, Zodpey SP, Babar VY**. 2003. Hypertension among cotton textile workers. Indian journal of public health 47(1):34-36.	❖						
**Tomei F, De Sio S, Tomao E, Anzelmo V, Baccolo TP, Ciarrocca M, Cherubini E, Valentini V, Capozzella A, Rosati MV**. 2005. Occupational exposure to noise and hypertension in pilots. International Journal of Environmental Health Research 15(2):99-106.					❖		
**Tomei F, Fantini S, Tomao E, Baccolo TP, Rosati MV**. 2000. Hypertension and chronic exposure to noise. Archives of Environmental Health 55(5):319-325.					❖		
**Tomei F, Papaleo B, Baccolo TP, Tomao E, Alfi P, Fantini S**. 1996. Chronic exposure to noise and effects on the cardiovascular apparatus in airplane pilots. [Italian]. Medicina del Lavoro 87(5):394-410.					❖		
**Tomei F, Tomao E, Baccolo TP, Papaleo B, Alfi P**. 1992. Vascular Effects of Noise. Angiology 43(11):904-912.					❖		
**Tomei F, Tomao E, Papaleo B, Baccolo TP, Alfi P**. 1991. Study of some cardiovascular parameters after chronic exposure to noise. Int J Cardiol 33(3):393-9.					❖		

Reference (e: exposition; e: comparison; o: outcome; p: population; n: no response given; r: response<10%; s: study design)	Reason for exclusion						
	e	c	o	p	n	r	s
**Tomei F, Tomao E, Papaleo B, Baccolo TP, Cirio AM, Alfi P**. 1995. Epidemiological and clinical study of subjects occupationally exposed to noise. International Journal of Angiology 4(2):117-121.					❖		
**Tomei G, Sancini A, Tomei F, Vitarelli A, Andreozzi G, Rinaldi G, Di Giorgio V, Samperi I, Fiaschetti M, Tasciotti Z, Cetica C, Capozzella A, Ciarrocca M, Caciari T**. 2013. Prevalence of Systemic Arterial Hypertension, Electrocardiogram Abnormalities, and Noise-Induced Hearing Loss in Agricultural Workers. Archives of Environmental & Occupational Health 68(4):196-203.					❖		
**Tong JW, Wang Y, Yuan JX, Yang JB, Wang ZY, Zheng Y, Chai F, Li XW**. 2017. Effect of Interaction Between Noise and A1166C Site of AT1R Gene Polymorphism on Essential Hypertension in an Iron and Steel Enterprise Workers. Journal of Occupational and Environmental Medicine 59(4):412-416.		❖			❖		
**Utari S, Sutisna E, Rinawati S, Astrika YF, Hardiningsih, Eka NYA**. 2018. The Impact of Industrial Noise Exposure on Hearing Loss and Hypertension Decline of Labour in Central Java. Atlantis Press, Paris.	❖				❖		
**Van Dijk FJH, Ettema JH, Zielhuis RL**. 1986. Non-auditory effects of noise in industry - I. Introduction and study objectives. International Archives of Occupational and Environmental Health 58(4):321-323.							❖
**Vangelova KK, Deyanov CE**. 2007. Blood pressure and serum lipids in industrial workers under intense noise and a hot environment. Reviews on Environmental Health 22(4):303-311.					❖		
**Verbeek JHAM, van Dijk FJH, de Vries FF**. 1987. Non-auditory effects of noise in industry - IV. A field study on industrial noise and blood pressure. International Archives of Occupational and Environmental Health 59(1):51-54.		❖	❖				
**Vermel AE, Zinenko GM, Kochanova EM, Suares LT, Bogatov KM**. 1988. Intensity of industrial noise and the incidence of arterial hypertension (according to data from a prospective epidemiological study of organized female populations in Moscow. Terapevticheskii arkhiv 60(9):88-91.					❖		

**Reference**	(e: exposition; e: comparison; o: outcome; p: population; n: no response given; r: response<10%; s: study design)	**Reason for exclusion**						
		**e**	**c**	**o**	**p**	**n**	**r**	**s**
**Virkkunen H, Harma M, Kauppinen T, Tenkanen L**. 2007. Shift work, occupational noise and physical workload with ensuing development of blood pressure and their joint effect on the risk of coronary heart disease. Scandinavian Journal of Work Environment & Health 33(6):425-434.								❖
**Vlasova EM, Shliapnikov DM, Lebedeva TM**. 2015. Analysis of changes in characteristics of arterial hypertension occupational risk in workers of nonferrous metallurgy. Meditsina truda i promyshlennaia ekologiia(8):10-13.		❖[6]				❖		
**Wang DM, Zhou M, Li WZ, Kong WJ, Wang ZC, Guo YJ, Zhang XM, He MA, Guo H, Chen WH**. 2018. Occupational noise exposure and hypertension: the Dongfeng-Tongji Cohort Study. Journal of the American Society of Hypertension 12(2):71-79.						❖		
**Wang H**. 2004. The relationship between incidence of cardiovascular disease and density of noise. Occup Health & Emerg Rescue 22(4).						❖		
**Wanis Osiris G, Abd-Elfattah Mohamed A, Shawky Hany A**. The association between noise exposure and blood pressure and ECG of workers in egyptian factories; 2014. International Institute of Acoustics and Vibrations. p 64-75.			❖			❖		
**Wojtczak-Jaroszowa J, Jarosz D**. 1986. Health complaints, sicknesses and accidents of workers employed in high environmental temperatures. Canadian Journal of Public Health 77(SUPPL. 1):132-135.						❖		
**Wu TN, Ko Y-C, Chang P-Y**. 1987. Study of noise exposure and high blood pressure in shipyard workers. American Journal of Industrial Medicine 12(4):431-438.						❖		
**Wu TN, Shen CY, Ko KN, Guu CF, Gau HJ, Lai JS, Chen CJ, Chang PY**. 1996. Occupational lead exposure and blood pressure. Int J Epidemiol 25(4):791-6.				❖		❖		

[6] no definition of noise exposure in the control group (control group only described as working in engineering)

**Reference** (e: exposition; e: comparison; o: outcome; p: population; n: no response given; r: response<10%; s: study design)	**Reason for exclusion**						
	**e**	**c**	**o**	**p**	**n**	**r**	**s**
**Wu X, Yang D, Fan W, Fan C, Wu G**. 2017. Cardiovascular risk factors in noise-exposed workers in china: Small area study. Noise and Health 19(91):245-253.		❖			❖		
Xiao Q, Xiao L, Du W. 2008. Effect of Occupational Noise Exposure on the Cardiovascular System. Occup Health 24(1):11-12.					❖		
**Yaghoubi K, Alimohammadi I, Abolghasemi J, Shirin Shandiz M, Aboutaleb N, Ashtarinezhad A**. 2018. The effect of occupational noise exposure on systolic blood pressure, diastolic blood pressure and salivary cortisol level among automotive assembly workers. International Journal of Occupational Safety and Ergonomics.			❖		❖		
**Zaborski L, Szczepanski C, Waskiewicz J**. 1981. The effect of vibratory and acoustic conditions on ships upon the circulatory system during sea voyages. Bulletin of the Institute of Maritime and Tropical Medicine in Gdynia 32:73-85.			❖				
**Zamanian Z, Rostami R, Hasanzadeh J, Hashemi H**. 2013. Investigation of the effect of occupational noise exposure on blood pressure and heart rate of steel industry workers. J Environ Public Health 2013:256060.							❖[7]
**Zhao Y, Wang L, Pan D, Ji Y, Pan Q, Wang H**. 1998. A dose response relationship for noise induced hypertension in chemical fertilizer factories. Noise effects '98. 1(Proceedings on the 7th International Congress on Noise asa Public Health Problem, 22–26 November 1998 (Carter N,Job RFS, eds)):259-263.					❖		
**Zhou F, Shrestha A, Mai S, Tao Z, Li J, Wang Z, Meng X**. 2019. Relationship between occupational noise exposure and hypertension: A cross-sectional study in steel factories. Am J Ind Med 62(11):961-968.	❖	❖					
**Total**	28	47	32	1	92	3	12

7 experimental study

The following studies could not be obtained:

- **Polizzi S, Discalzi G, Cappellaro F, Mocellini A, Cifaldi M, Catale M, Scanzetti G**. 1995. Effetti extrauditivi dell'esposizione a rumore: ipertensione arteriosa. 58° Congresso Nazionale S.I.M.L.I.I., 1: Relazioni:367-71.
- **Singh LP**. 2013. An Investigation into Blood Pressure of Blue Collar Workers of Casting and Forging SMEs: A Study in India.
- **Tomei F, Corrao C, Romana N, Tomao A, Villarini S, Baccolo T, Tomei F, Nanni Nanni G, Rosati MV**. 1998. Effetti cardiovascolari ed esposizione a rumore in lavoratori agricoli. Folia Medica 69:709-714.
- **Van Dijk F**. 1984. Closing industrial investigation. In: Effects of Noise on Health and Well Being in Industry [PhD Thesis]. University of Amsterdam, Amsterdam. S. 131-196.
- **Van Dijk F**. 1984. Industrial noise and blood pressure, s'Hertogenbosch. In: Effects of Noise on Health and Well Being in Industry [PhD Thesis]. University of Amsterdam, Amsterdam. S. 84-90.
- **Van Dijk F**. 1984. Research at a shipyard. In: Effects of Noise on Health and Well Being in Industry [PhD Thesis]. University of Amsterdam, Amsterdam. S. 101-130.

Table S3. Characteristics of included case-control studies

Study	Study region	Time of recruit-ment	Cases	Control Group	Exposure
**Siagian et al. 2009**<br><br>*Nested case-control study of Indonesian Air Force pilots (N=567) attending annual medical examination*	Indonesia	2003-2008	No. of incident cases with DBP≥90 mmHg: n=40<br><br>Age at time of diagnosis: n (%)<br>23-29 yrs. 9 (22.5)<br>30-39 yrs. 22 (55.0)<br>40-48 yrs. 9 (22.5)<br><br>Sex:<br>*not reported (presumably 100% male)*<br><br>Response:<br>100 % (obligatory screening)	No. of controls with DBP< 89 mmHg: n=480<br><br>Age at time of diagnosis: n (%)<br>23-29 yrs. 168 (35.0)<br>30-39 yrs. 253 (52.7)<br>40-48 yrs. 59 (12.3)<br><br>Sex:<br>*not reported (presumably 100% male)*<br><br>Response:<br>100 % (obligatory screening)	Interior aircraft noise<br><br>No information on how this was estimated. Methods of noise measurements, number of measurements, and duration of measurements were not given.<br><br>HPD use not reported.

Abbreviations: yrs., year(s); DBP, diastolic blood pressure

Table S4. Results shown in included case-control studies

Study	Noise exposure dB(A)	Exposed cases N (%)	Exposed controls N (%)	Effect estimate	Effect value (95% CI)	Adjusted for	Remarks
**Siagian et al. 2009**	70-80 (Ref.)<br>90-95	24 (60.0)<br>16 (40.0)	363 (75.6)<br>117 (24.4)	OR	1.00 (Reference)<br>**2.70 (1.05-6.97)**	Resting pulse rate and total flight hours.<br><br>*Unclear if adjusted for other unnamed risk factors.*	Unadjusted OR 2.07 (1.06-4.03)

Abbreviations: CI, confidence interval; h, hour(s); n, sample size; OR, odds ratio; Ref., Reference

Table S5. Characteristics of included cohort and cross-sectional studies

Study	Study design	Study region	Time of recruitment/ follow-up	Population & Study recruitment (working environment, industries, job duties etc.)		Outcome
				**Exposure group**	**Control group**	
**Attarchi et al. 2013**	Cross-sectional	Iran	2010-2011	Workers in the car manufacturing industry (paint and assembly location)<br><br>No. of exposed:<br>Group 2 (solvents group) n=101<br>Group 3 (noise group): n=139<br>Group 4 (co-exposure group):	Office workers in the car manufacturing industry<br><br>No. of unexposed:<br>Group 1: n=124<br><br>Age (mean, SD not given): 32.5 yr.	<u>Hypertension</u>: SBP≥140 mmHg and/or DBP≥90 mmHg and/or physician-diagnosed hypertension<br><br>*People with history of*

				n=107<br><br>Age (mean, SD not given):<br>Group 2: 33.1 yr.<br>Group 3: 33.3 yr.<br>Group 4: 32.5 yr.<br><br>Sex: 100 % male<br><br>Response: 100 %<br>*("all workers who had experienced the working conditions for more than six months were included"; "all workers participated voluntarily in this study" p. 245)*<br><br>Noise and solvent assessment<br>**Noise:** stationary measurements at 47 work places for 8 h, twice during one week and 8 h equivalent continuous noise level were calculated. Each worker was assigned a particular noise level for his working station.<br><br>**Solvents** (acetone, benzene, tetrachloroethylene, toluene and	Sex: 100 % male<br><br>Response: see left<br><br>Noise and solvent assessment<br>see left<br><br>Noise and solvent exposure (mean ± SD):<br>low exposure to noise (65.5 ± 3.6 dB(A)) and no exposure to solvents<br>Years of exposure (mean, SD not given): 7.7 years	*hypertension during pre-employment medical examination were excluded (p.244, r. column, last sentence). A blood pressure measurement was not done during the pre-employment medical examination, so unknown cases of hypertension at entry cannot be ruled out. The detected hypertension cases may in part be prevalent cases and not incident.*

				xylene): stationary measurements during all working hours of one day at 21 work places using a pump with charcoal tube, separated by gas chromatography. The method of detection was not reported.<br><br>Noise and solvent exposure (mean ± SD):<br>**Group 2 (solvents group)**:<br>Paint location 1 (n=101): noise below the TLV of 85 dB(A) (81.7 ± 1.7 dB(A); high exposure to solvents (mean equivalent exposure[1] 1.78, SD not given).<br><br>**Group 3 (noise group)**:<br>Assembly line (n=139): noise above the TLV of 85 dB(A) (89.0 ± 2.6 dB(A); no solvent exposure.<br><br>**Group 4 (co-exposure group):**<br>Paint location 2 (n=107): Exposure to noise above the TLV of 85 dB(A) (91.0 ± 3.5 dB(A) and to solvents (mean equivalent exposure[1] 2.53, SD not given).		

				Years of exposure (in current job):<br>Group 2: 8.7 yr.<br>Group 3: 7.9 yr.<br>Group 4: 7.6 yr.<br><br>HPD:<br>Group 2: NA<br>Group 3: 17.9 %<br>Group 4: 18.6 %		
**Brahem et al. 2018**	Cross-sectional	Tunisia	2017	Workers at an electric power station<br><br>No. of exposed: 120<br><br>Age (mean ± SD): 41.9 ± 10 yr.<br><br>Sex:<br>male to female ratio approximately 7:1 (about 87.5% males, 12.5% females)<br><br>*"The exposed and non-exposed group were selected with regard to age and sex." Age and sex distribution for the different exposure groups were not reported. Exposed and non-exposed subjects were matched according to age*	Type of industry: see left<br><br>No. of unexposed:<br>< 80 dB: n=120<br><br>No information to the number and duration of noise measurements<br>Years of exposure: no information<br><br>Age: see left<br><br>Sex: see left<br><br>Response rate: 100 %<br><br>Noise exposure assessment<br>see left	Hypertension:<br>SBP≥140 mmHg and/or DBP≥90 mmHg,<br><br>*Workers with self-reported (questionnaire) hypertension prior to working at the power station were excluded (personal communication, A. Brahem).*

				and sex (personal communication; A. Brahem).<br><br>Response: 100%<br><br><u>Noise exposure assessment</u><br>Noise exposure was measured using a class 1 "technocome" SP140 portable precision sound level meter after establishing a noise cartography of the company and locating the sources of noise and noise barriers. Each measurement was done close to the particular machine at ear level and took about one minute *(personal communication; A. Brahem)*.<br><br><u>Noise exposure</u><br>The subjects were exposed to noise levels between 75 and 103 dB (mean: 89 dB).<br><br>Years of exposure:<br>Not reported. *Only people who had worked at the station for at least one year were included in the study.*	<u>Noise exposure</u><br>Non-exposed subjects were exposed to less than 80 dB *(personal communication; A. Brahem)*	

				HPD: not reported		
**Chang et al. 2009**	Cross-sectional	Taiwan (ROC)	2005-2006	Workers in the synthetic leather manufacturing industry (product sampling test, non-woven fabric manufacturing, wet and dry processes, printing, pressing, surface arrangement and inspection)<br><br>No. of exposed:<br>Group 1 (co-exposure group): n=18<br>Group 2 (noise): n=9<br>Group 3 (lower noise, solvents): n=15<br><br>Age (mean ± SD):<br>Group 1: 40.3 ± 5.8 yr.<br>Group 2: 34.6 ± 7.7 yr.<br>Group 3: 41.6 ± 9.1 yr.<br><br>Sex:<br>Group 1: 94.1 % male; 5.6 % female<br>Group 2: 77.8 % male; 22.2 % female<br>Group 3: 86.7 % male; 13.3 % female<br><br>Response: 90.8 % in the total group,	Office workers in a synthetic leather manufacturing plant<br><br>No. of unexposed: n=17<br><br>Age (mean ± SD): 37.6 ± 7.5 yr.<br><br>Sex:<br>58.8 % male; 41.2 % female<br><br>Response: see left<br><br>Noise and solvent assessment<br>see left<br><br>Noise and solvent exposure (mean ± SD):<br>low exposure to noise (72.81 ± 2.16 dB(A) and low exposure to solvents (N,N-dimethylformamide and toluene, hazard index 0.04 ± 0.01)<br><br>Years of exposure: mean ± SD:<br>7.6 ± 1.5 yr.	Hypertension:<br>SBP≥140 mmHg and/or DBP≥90 mmHg and/or physician-diagnosed hypertension

				no information according response in the different exposure groups<br><br><u>Noise and solvent assessment</u><br>Each subject carried a logging noise dosimeter and average noise exposure was calculated from 5-min. readings during the working period of 10h.<br><br>Each subject carried a personal pump with a charcoal tube to measure solvents (N,N-dimethylformamide and toluene) during one day.<br><br><u>Noise and solvent exposure (mean ± SD)</u>:<br><br>**Group 1** (n=18)**:** combined exposure to noise (82.22 ± 2.70 dB(A) and solvents (hazard index[1] 0.53 ± 0.20)<br><br>**Group 2** (n=9)**:** exposure to noise (84.13 ± 2.30 dB(A) but lower exposure to solvents (hazard index 0.03 ± 0.02)		

				**Group 3** (n=15)**:** lower exposure to noise (75.20 ± 1.84 dB(A) but exposure to solvents (hazard index 0.32 ± 0.18)<br><br>Years of exposure (mean ± SD):<br>Group 1: 7.9 ± 0.5 yr.<br>Group 2: 7.4 ± 1.3 yr.<br>Group 3: 7.5 ± 1.9 yr.<br><br>HPD:<br>Group 1: 5.6 % (n=1)<br>Group 2: 55.6 % (n=5)<br>Group 3: 0 % (n=0)		
**Chang et al. 2012**	Cross-sectional	Taiwan (ROC)	2009	Workers in the screw-manufacturing industry (metal cutting, pressing, grinding, sand blasting, polishing and gear washing) with high noise exposure > 80 dB(A) according the personal noise measurement.<br><br>No. of exposed: n=68<br><br>Age (mean ± SD):<br>32.4 ± 6.4 yr.	Reference group 1: Workers in the screw-manufacturing industry (metal cutting, pressing, grinding, sand blasting, polishing and gear washing) with low noise exposure < 80 dB(A) according the personal noise measurement<br><br>Reference group 2: office workers in the plant.<br><br>No. of unexposed:	<u>Hypertension</u>: SBP≥140 mmHg and/or DBP≥90 mmHg and/or physician-diagnosed hypertension.<br><br>*Workers reporting a diagnosis of hypertension prior to beginning work in the factory (n=9) were*

				Sex:<br>97.1 % male, 2.9 % female<br><br>Response: 71.9 %<br>*The authors report no significant difference between responders and non-responders in terms of educational level, cigarette smoking, alcohol drinking, tea or coffee consumption, regular exercise, working activity or family history of hypertension. No information on the response according to exposure groups given.*<br><br>Noise assessment<br>2 different methods used to measure noise:<br>1. Each subject carried a logging noise dosimeter, 5 minute readings over a period of 8 h and TWA noise exposure was calculated.<br><br>2. Stationary measurements at 14 locations using an octave-band analyzer. The TWA during 8 h was assessed at the frequencies of 31.5,	Group 1: n=68<br>Group 2: n=52<br><br>Age (mean ± SD):<br>Group 1: 31.9 ± 5.5 yr.<br>Group 2: 33.4 ± 6.7 yr.<br><br>Sex:<br>Group 1: 92.7 % male; 7.3 % female<br>Group 2: 50.0 % male; 50.0 % female<br><br>Response: see left<br><br>Noise assessment<br>see left<br><br>Noise exposure (mean ± SD):<br>Personal noise level:<br>Group 1: 75.8 ± 3.2 dB(A),<br>Group 2: 61.5 ± 0.5 dB(A)<br><br>Stationary noise level (dB(A)):<br>Group 1:<br>all frequencies: 79.2 ± 4.7,<br>31.5 Hz: 32.9 ± 3.4,<br>63 Hz: 45.2 ± 4.8,<br>125 Hz: 52.9 ± 6.5,	*excluded.*<br><br>*A blood pressure measurement was not done at entry into employment at the factory, so an unknown number of prevalent hypertension cases at entry cannot be ruled out. The detected hypertension cases in the study are therefore at least in part prevalent cases and not incident.*

				63, 125, 250, 500, 1,000, 2,000, 4,000 and 8,000 Hz in dB(A) (mean ± SD):<br><br><u>Noise exposure (mean ± SD):</u><br>Personal noise level: 82,7 ± 6.7<br><br>Stationary noise level:<br>all frequencies: 82.3 ± 4.4,<br>31.5 Hz: 32.6 ± 2.6,<br>63 Hz: 47.1 ± 6.1,<br>125 Hz: 54.8 ± 4.9,<br>250 Hz: 61.5 ± 4.8,<br>500 Hz: 67.8 ± 5.4,<br>1,000 Hz: 71.4 ± 6.6,<br>2,000 Hz: 70.3 ± 6.4,<br>4,000 Hz: 72,.3 ± 5.9,<br>8,000 Hz: 72.2 ± 5.7 dB(A)<br><br>Years of exposure (mean ± SD): 3.8 ± 2.7<br><br>HPD: 0 % (n=0)<br>*5 workers using HPD were excluded from the study*	250 Hz: 59.7 ± 7.8,<br>500 Hz: 64.3 ± 7.5,<br>1,000 Hz: 63.0 ± 5.6,<br>2,000 Hz: 63.7 ± 5.1,<br>4,000 Hz: 66.4 ± 5.5,<br>8,000 Hz: 65.0 ± 6.4 dB(A)<br><br>Group 2 (mean; SD not reported presumably because only one measurement was made):<br>all frequencies: 62.4,<br>32.5 Hz: 33.3,<br>63 Hz: 44.6,<br>125 Hz: 50.8,<br>250 Hz: 53.9,<br>500 Hz: 50.0,<br>1,000 Hz: 61.2,<br>2,000 Hz: 57.8,<br>4,000 Hz: 53.6,<br>8,000 Hz: 50.5<br><br>Years of exposure (mean ± SD):<br>Group 1: 3.8 ± 3.3,<br>Group 2: 4.2 ± 3.3	
**Chang et al. 2013**	Retrospective cohort	Taiwan (ROC)	1998-2008	Workers in an aircraft manufacturing plant, recruited during annual occupational health	Type of industry: see left<br><br>No. of unexposed: n=205	<u>Hypertension</u>: SBP≥140 mmHg and/or diastolic

	(follow-up study to a cross-sectional survey)			examination<br><br>No. of exposed:<br>High-exposure group: n=152<br>Intermediate-exposure group: n=221<br><br>Age at entry (mean ± SD):<br>All subjects: 27.7 ± 5.3 yr.<br>High-exposure group: 27.6 ± 4.6 yr.<br>Intermediate-exposure group: 27.5 ± 5.4 yr.<br><br>Sex: 100 % Male<br><br>Response: 74.1 %<br>*not differentiated between exposed and non-exposed workers*<br><br>Lost to follow-up: 25.9 %<br>*retired or no follow-up-result in 2008*<br><br>Noise assessment<br>A 15 minute-TWA was measured at 337 locations ("possibly the loudest workplaces", p.819) using a sound analyser. An additional 8h-TWA was measured at the 121 workplaces	 Age at entry (mean ± SD):<br>28.0 yr. (±5.6)<br><br>Response: see left<br><br>Follow-up: see left<br><br>Noise assessment<br>see left<br><br>Noise exposure (mean*± SD):<br>Low-exposure group (n=205):<br><80 dB(A)<br>71.9±9.0 dB(A)<br><br>HPD: 41.0% (n=84)	blood pressure ≥90 mmHg and/or physician-diagnose d<br>10-year-risk of hypertension (total hypertension with additional RR-calculations for the subgroups diagnosed versus measured hypertension)

				with a 15 minute-TWA of ≥65 dB(A) on the basis of which each worker was assigned to a certain value of noise exposure. Workers were divided into exposure groups based on their tasks and working processes.<br><br>*Noise exposure was adjusted for the use of HPD, assuming the following noise reductions: 29dB for earplugs, 25dB for earmuffs.*<br><br><u>Noise exposure (mean* ± SD):</u><br>**after adjustment for HPD*<br>High-exposure group (n=152):<br>≥85 dB(A)<br>86.9 ± 2.2 dB(A)<br><br>Intermediate-exposure group (n=221):<br>80-<85 dB(A)<br>83.0 ± 1.3 dB(A)<br><br>HPD: 69.6% (total population)<br>High-exposure group: 74.3% (n=113)<br>Intermediate-exposure group: 92.8%		

				(n=205)		
**Chen et al. 2005**	Cross-sectional	Peoples Republic of China	Not given	Two metal processing plants with the following work places: riveting, welding, cast cleaning, and electrical installation.<br><br>Number of exposed:<br>Riveters: n=419<br>Welders: n=525<br>Cast cleaners: n=159<br>(other workers)<br>Electrical installers: **Number of electrical installers not given. They were grouped together with electricians as other workers (n=102)<br><br>Age:<br>median: 35-40 years<br>18-29 yr. 221<br>30-34 yr. 327<br>35-39 yr. 294<br>40-44 yr. 168<br>45-49 yr. 106<br>50-58 yr. 89	Type of industry: See left<br><br>Number of unexposed:<br>Workers with low exposure 73-80 dB(A)<br>Electricians: **see left<br><br>Noise assessment<br>see left<br><br>Noise exposure<br>Cumulative exposure (CNE):<br>70-<85 dB(A) x years: n=100	Hypertension:<br>SBP≥140 mmHg and/or diastolic blood pressure ≥90 mmHg and/or intake of antihypertensive drugs

				Sex:<br>72.8% male; 27.2% female<br><br>Response: 89.59%<br>*not differentiated between the different exposure groups*<br><br><u>Noise assessment</u><br>Measured for 8 h for one worker at each working place. The equivalent TWA for 8h and the cumulative noise exposure<br>CNE=<br>$(10\times\log \ (\sum 10^{0.1\times Log(A)} \times$exposure time)<br>was calculated were A is the equivalent TWA for 8h (Leq) in dB(A) and the exposure time is the noise exposure in years. The unit of CNE is dB(A) x years.<br><br><u>Noise exposure</u><br>Number of exposed workers:<br>Riveters: n=419, Leq: 85-87 dB(A)<br>Welders: n=525, Leq: 70-84 dB(A)<br>Cast cleaners: n=159, Leq: 91-94 dB(A)<br>Electro installers**: 81-83 dB(A)		

				Cumulative noise exposure (CNE):<br>85-<90 dB(A) x years: n=47<br>90-<95 dB(A) x years: n=286<br>95-<100 dB(A) x years: n=592<br>100-<105 dB(A) x years: n=161<br>≥105 dB(A) x years: n=19<br><br>Exposure time per day: 8 h<br><br>Years of exposure: Not reported.<br>*Only people who had worked for at least six months were included in the study.*<br><br>HPD: 23.49% (total population, not differentiated between exposure groups)		
**De Souza et al. 2015**	Cross-sectional (secondary data)	Brazil	2007	Sub-contractors working at least 6 months in the petrochemical and gas refinery industry (maintenance, construction and expansion) recruited during the mandatory annual physical exam.<br><br>No. of exposed:<br>low exposed. n=871	Type of industry: See left<br><br>No. of unexposed: 388<br><br>Age (n (%)):<br><30 83 (21.39)<br>30-34 80 (20.62)<br>35-39 56 (14.43)	Hypertension:<br>SBP≥140 mmHg and/or DBP≥90 mmHg and/or doctors diagnosed hypertension

				high exposed: n=470<br><br><br>Age (n (%)):<br>75-80 dB(A)/ ≥ 85 dB(A)<br><30 yrs. 186 (21.35) / 89 (18.94)<br>30-34 yrs. 145 (16.65) / 81 (17.23)<br>35-39 yrs. 58 (6.67) / 78 (16.60)<br>40-44 yrs. 147 (23.54) / 84 (17.87)<br>45-49 yrs. 107 (12.28) / 62 (13.19)<br>≥50 yrs. 128 (14.70) / 76 (16.17)<br><br>Sex:<br>low exposed:<br>93.1 % male; 6.9 % female<br>high exposed:<br>99.6 % male; 0.4 % female<br><br>Response: 100 %<br><br>Noise assessment<br>Noise was measured with a logging	40-44 42 (10.82)<br>45-49 47 (12.11)<br>≥50 80 (20.62)<br><br>Sex:<br>78.9 % male; 21.1 % female<br><br>Response: 100 %<br><br>Noise assessment<br>see left<br><br>Noise exposure<br>≤ 75 dB(A)<br><br>Years of exposure: not reported	

				noise dosimeter affixed next to the ears of a randomly selected worker in a homogeneous exposure group. The measurement was done at a single moment in cases of continuous exposure to noise and during at least 75 % of a workday of 8h. In case of intermittent exposure noise measured according the governmental regulation. The number of noise measurements was not given.<br><br><u>Noise exposure</u><br>Noise exposure was categorized as low (75-85 dB(A)) and high exposed (≥ 85 dB(A)).<br><br>Years of exposure: min. 0.5 yr.<br>*no further information*<br><br>❖ HPD: not reported		
**Fogari et al. 1994**	Cross-sectional	Italy	Not mentioned	Metallurgical factory without information according the type of production and the job duties. The mentioned company is known to be	Type of industry: See left<br><br>No. of unexposed:<br>No. with low exposure ≤ 80 dB: total	<u>Hypertension:</u><br>DBP≥95 mmHg

				a producer of helicopters and motorcycles.<br><br>No. of exposed:<br>Total group (> 80 dB): n=733<br>Matched group (> 85 dB): n=242<br><br>Age (mean ± SD):<br>*No information on the age of all participants in this publication but according of Fogari et al. (1995) the age of all screened persons with high and low exposure (n=8,811) was 39.1 ± 7.4 yrs. The age of the high and low exposed group is not given.*<br>Matched group: 38.3 ± 6.2 yrs.<br><br>Sex:<br>*No information in this publication but according to Fogari et al. (1995) 9.4 % of all screened persons with high and low exposure (n=8,811) were female and 90.6 % male. The sex distribution of the high and low exposed group is not given.*<br>Matched group: 100 % male<br><br>Response:	group 8078, matched group: 242<br><br>Age (mean ± SD):<br>total group: see left,<br>matched group: 38.2 ± 6.1 yrs.<br><br>Sex: see left<br><br>Response: see left<br><br><u>Noise assessment</u><br>see left<br><br><u>Noise exposure</u><br>low exposure ≤ 80 dB<br><br>Years of exposure (mean ± SD): no information in the total group and 11.1 ± 2.0 years in the matched group	

				No information in this publication but according to Fogari et al. 1995 the response of all screened persons with high and low exposure (n=8,811) was 94 %. The response of the high and low exposed groups were not reported. No information to the response of the matched group.<br><br><u>Noise assessment</u><br>Measured with a sound meter. The numbers and length of noise measurements were not given. No information according the type of measurement (stationary or personally). The mentioned sound meter is known to be a stationary instrument.<br><br><u>Noise exposure</u><br>Total group (n=733): > 80 dB<br>Matched group (n=242): > 85 dB<br><br>Years of exposure (mean ± SD):<br>Total group: no information<br>Matched group: 11.3 ± 2.5 yrs.		

				HPD: not reported		
**Fogari et al. 1995**	Cross-sectional	Italy	Not mentioned	Study recruitment (working environment, industries, job duties etc.) see Fogari et al. (1994)<br><br>No. of exposed<br>> 80 dB: n=733<br><br>Years of exposure: No information<br><br>Age (mean± SD):<br>in the total group of 8,811 workers: 39.1 ± 7.4 years, no information according the age distribution in the groups with different noise exposure<br><br>Sex:<br>in the total group of 8,811 workers: 90.6 % male and 9.4 % female, no information according the sex distribution in the groups with different noise exposure<br><br>Response: 94% (in the total group of 8,811 workers)	Type of industry: See left<br><br>No. of unexposed:<br>≤ 55 dB: n=3648[1] (41,4 %)<br>55-80 dB: n=4405[1] (50,0 %)<br><br>Age: see left<br><br>Sex: see left<br><br>Response: see left<br><br><u>Noise assessment</u><br>see Fogari et al. (1994)<br><br><u>Noise exposure</u><br>≤ 55 dB<br>55-80 dB	<u>Hypertension</u>:<br>> 140/90 mmHg or intake of antihypertensive drugs

				Response in the high and low exposed groups not given.<br><br><u>Noise assessment</u><br>noise measurement see Fogari et al. (1994)<br><br><u>Noise exposure</u><br>> 80 dB<br><br>Years of exposure: not reported<br><br>HPD: not reported		
**Fokin et al. 2018**	Retrospective cohort	Russia	Not mentioned	Food industry (transport workers, packaging workers, forklift drivers, food controllers)<br><br>No. of exposed<br>> 80 dB(A): n=21<br><br>Age (mean ± SD):<br>48 ± 3.9 years (range: 21-64yr.)<br><br>Sex:<br>19 % male; *81 % female (*self-calculated)	Food industry (Laundry workers, mixing workers, car drivers, electric cart drivers, mechanics, electricians, instrument and equipment fitters and transport workers)<br><br>No. of unexposed:<br>< 80 dB(A): n=28<br><br>Age (mean ± SD):<br>48.8 ±3.6 years (range: 29-66yr.)<br><br>Sex:	<u>Hypertensive diseases</u> Incidence of hypertension (ICD 10: I10-I15) according the data of the compulsory health insurance.<br><br>*The authors didn't report that cases with hypertension which were diagnosed before the onset of exposure*

				Response: 100 %<br>*Secondary data analysis of compulsory health insurance data*<br><br>Noise assessment<br>Measurement methods not reported<br><br>Noise exposure<br>> 80 dB(A)<br><br>Years of exposure: 17.1 ±3.4 yrs.<br>*Only workers working at least five years were included.*<br><br>HPD use: not reported	53.6% male, 46.4% female<br><br>Response: 100 %<br>*Secondary data analysis of compulsory health insurance data*<br><br>Noise assessment<br>see left<br><br>Noise exposure<br>< 80 dB(A)<br><br>Years of exposure: 15 ± 3.3 yrs.<br>*Only workers working at least five years were included.*	*were excluded. Therefore it is doubtful that the observed cases of hypertension are really incident cases.*
**Giordano et al. 2001**	Cross-sectional	Italy	Not mentioned / unclear	Factory workers in a metallurgical and mechanical company (Job duties not given)<br><br>No. of exposed:<br>> 70 dB(A): n=100<br><br>Age:<br>mean 43 yrs.; range 20-60 yrs.	Office workers in a metallurgical and mechanical company (Job duties not given)<br><br>No. of unexposed:<br>< 70 dB(A): n=100<br><br>Age:<br>mean 45 yrs.; range 24 - 58 yrs.	Hypertension:<br>Definition not given. Self-reported anamnestic information on arterial hypertension.

				Sex: male: 64 %; female: 36 %,<br><br>Response: 100 %<br><br><u>Noise assessment</u><br>Measurement methods not reported<br><br><u>Noise exposure</u><br>> 70 dB(A)<br><br>Years of exposure:<br>2 - 35 yrs. (mean not given)<br><br>HPD: not reported	Sex:<br>male: 54 %; female: 46 %,<br><br>Response: 100 %<br><br><u>Noise assessment</u><br>see left<br><br><u>Noise exposure</u><br>< 70 dB(A)<br><br>Years of exposure:<br>2 - 37 yrs. (mean not given)	
**Ha and Kim, 1991**	Cross-sectional	Republic of Korea	1990	Steel mill workers working in production, administration, and general management *(women and administrative personnel whose age, smoking, educational, and employment length characteristics greatly differed from production workers were excluded)*<br><br>No. of exposed: n=1034<br><br>Age (mean ± SD):	Type of industry: see left<br><br>No. of unexposed: n=390<br><br>Age (mean ± SD):<br>35.7 ± 6.3 yrs.<br><br>Sex: 100% male<br><br>Response: see left	<u>Hypertension</u>:<br>SBP≥160 mmHg or DBP≥100 mmHg<br><br><u>Borderline Hypertension</u>:<br>SBP≥150 to <160 mmHg or DBP≥95 to <100 mmHg

				37.0 ± 7.4 yrs.<br><br>Sex:<br>100% male<br><br>Response: 86.6 %<br>*no information according the response rate in relation to noise exposure*<br><br>Noise assessment<br>Measurement methods not reported<br><br>Noise exposure (mean ± SD):<br>91.8 ± 5.2 dB(A)<br>min: 81.5 dB(A), max: 103.5 dB(A)<br><br>Years of exposure (mean ± SD):<br>Years of employment: 9.5 ± 5.1 yrs.<br>Years of noise exp.: 9.5 ± 5.7 yrs.<br><br>HPD: 73.5% (since 3 ± 2.6 yrs.)	Noise assessment<br>see left<br><br>Noise exposure (mean ± SD):<br>75.2 ± 4.6 dB(A)<br>min: 65.1 dB(A), max: 78.6 dB(A)<br><br>Years of exposure:<br>Years of employment: 9.2 ± 4.9 yrs.<br>Years of noise exp.: 5.1 ± 5.8 yrs.	
**Hwang et al. 2012**<br><br>*Cohort may overlap some with the*	Prospective cohort	Taiwan (ROC)	1988-2008 (20 yrs.)	Workers in the aircraft manufacturing industry<br><br>No. of exposed:<br>Low (50-64 dB(A)): n=324<br>Medium (65-80 dB(A)): n=178	Type of industry: see left<br><br>No. of unexposed:<br>40-49 dB(A): n=211<br><br>Age: see left	Hypertension:<br>SBP≥140 mmHg or DBP≥90 mmHg or physician-diagnose d hypertension

Chang et al. 2013 study population.				High (81-102 dB(A)): n=81<br><br>Age:<br><40 yr.: n=109<br>40-44 yr.: n=288<br>45-49 yr.: n=332<br>50-54 yr.: n=138<br>≥55 yr.: n=45<br>*no information on age distribution in relation to noise exposure*<br><br>Sex:<br>n=706 male; n=206 female<br>*Sex distribution of the total population (including workers not included in the analysis). No information according the sex distribution in relation to noise exposure.*<br><br>Response: 100 % at baseline,<br><br>Loss to follow up:<br>1988-2008: n=308 (23.7 %)<br><br>Noise assessment<br>Workplace noise assessment with sound analyzer at 332 locations (ca.	Sex: see left<br><br>Response: see left<br><br>Loss to follow-up: see left<br><br>Noise assessment<br>see left<br><br>Noise exposure<br>40-49 dB(A)<br><br>Years of exposure:<br>*no information on years of employment for the unexposed group*	*52 workers with hypertension identified at study begin were excluded*

				9 m²) conducted by industrial hygienist for 15 min. 121 locations with 15 min. TWA ≥65 dB(A), 8h were “further considered” (see Chang et al. 2013 for further details)<br><br>Workers were assigned to noise exposure categories based on similarity and frequency of tasks.<br><br><u>Noise exposure by years of exposure:</u><br>Low (50-64 dB(A)):<br>3-15 yrs. 162<br>>15 yrs. 162<br><br>Medium (65-80 dB(A)):<br>3-15 yrs. 90<br>>15 yrs. 88<br><br>High (81-102 dB(A)):<br>3-15 yrs. 27<br>>15 yrs. 54<br><br>HPD: not reported		

**Jegaden et al. 1986**	Cross-sectional	France	1984	Machine operators working in merchant marine, recruited during annual occupational health examination<br><br>No. of exposed: n=164<br><br>Age (mean ± SD):<br>46.8 ± 3.83 yrs.; range 40-55 yrs.<br><br>Sex: 100% male<br><br>Response: not reported (nearly all persons were included)<br><br>Noise assessment<br>Measurement methods not reported.<br><br>Noise exposure<br>95-115 dB(A) *for more than 5 to 6h per day*<br><br>Years of exposure:<br>Average exposure period was 25 yrs.<br><br>HPD: not reported	Merchant marines working on deck or as part of the service staff<br><br>No. of unexposed: n=291<br><br>Age (mean ± SD):<br>46.75 ± 3.94 yrs.; range 40-55 yrs.<br><br>Sex: 100% male<br><br>Response: see left<br><br>Noise assessment<br>see left<br><br>Noise exposure<br>50-75 dB(A)	Hypertension:<br>SBP ≥160 mmHg and/or DBP ≥95 mmHg (during occupational examination and confirmed by general practitioner or cardiologist) or the use of antihypertensive medication

**Liu et al. 2016**	Retrospective cohort	Taiwan (ROC)	1973-2012, median of follow-up 4.3 yr.	Workers in 4 machinery and equipment manufacturing companies exposed to noise from metal-cutting, pressing, grinding, sandblasting, polishing, and gear washing.<br><br>No. of exposed:<br>High exposure group: n=312<br><br>Age (mean ± SD):<br>37.4 ± 9.2 yrs.<br><br>Sex:<br>86.9 % male, 13.1 % female<br><br>Response: 55.8 %<br>*no information on the response*	Type of industry: see left<br><br>No. of unexposed:<br>Medium exposure group: n=203<br>Low exposure group: n=487<br><br>Age (mean ± SD):<br>Medium exposure group: 33.2 ± 6.9 yrs.<br>Low exposure group: 37.5 ± 7.9 yrs.<br><br>Sex:<br>Medium exposure group:<br>90.6 % male; 9.4 % female<br>Low exposure group:<br>73.1 % male; 26.9 % female<br><br>Response: see left	<u>Hypertension</u>:<br>SBP≥140 mmHg or DBP≥90 mmHg or physician-diagnosed hypertension or use of antihypertensive medication

				according to exposure groups given<br><br><u>Noise assessment</u><br>Noise was measured with 2 different methods:<br>1. Personal noise exposure was assessed by personal noise dosimeter to record 108 values of 5-minute continuous sound levels.<br><br>2. Stationary noise was measured using an octave band analyser at the frequencies of 31.5, 63, 125, 250, 500, 1,000, 2,000, 4,000 and 8,000 Hz in dB(A). 8-hour TWA equivalent sound levels were collected.<br><br><u>Noise exposure (mean ± SD)</u>:<br>**Personal noise level**<br>High exposure group: 84.2 ± 3.6 dB(A)<br><br>**Stationary noise level**<br>all frequencies: 79.4 ± 4.2 dB(A)<br>31.5 Hz: 35.3 ± 3.9 dB(A)<br>63 Hz: 47.2 ± 4.2 dB(A)<br>125 Hz: 55.7 ± 4.4 dB(A)	<u>Noise assessment</u><br>see left<br><br><u>Noise exposure (mean ± SD)</u><br>**Personal noise level**<br>Medium exposure group: 77.5 ± 1.7 dB(A)<br>Low exposure group: 67.3 ± 4.3 dB(A)<br><br>**Stationary noise level**<br>Medium exposure group:<br>all frequencies: 73.3 ± 6.1 dB(A)<br>31.5 Hz: 29.9 ± 4.1 dB(A)<br>63 Hz: 42.2 ± 5.2 dB(A)<br>125 Hz: 51.3 ± 4.6 dB(A)<br>250 Hz: 58.2 ± 5.4 dB(A)<br>500 Hz: 64.3 ± 4.8 dB(A)<br>1,000 Hz: 66.3 ± 4.1 dB(A)<br>2,000 Hz: 65.6 ± 3.6 dB(A)<br>4,000 Hz: 66.2 ± 4.5 dB(A)<br>8,000 Hz: 62.7 ± 5.0 dB(A)<br><br>Low exposure group<br>all frequencies: 58.0 ± 4.9 dB(A)<br>31.5 Hz: 25.9 ± 4.3 dB(A)	

				250 Hz: 62.8 ± 4.3 dB(A)<br>500 Hz: 68.5 ± 4.4 dB(A)<br>1,000 Hz: 71.4 ± 4.7 dB(A)<br>2,000 Hz: 72.2 ± 4.7 dB(A)<br>4,000 Hz: 73.0 ± 4.9 dB(A)<br>8,000 Hz: 70.4 ± 5.0 dB(A)<br><br>Years of exposure (mean ± SD):<br>8.0 ± 7.3 yrs.<br><br>HPD: 4.9% (total population);<br>high exposure group: 11.2% (n=35)<br>medium exposure group: 3.5% (n=7)<br>low exposure group: 1.4% (n=7)	63 Hz: 33.3 ± 4.8 dB(A)<br>125 Hz: 39.2 ± 5.1 dB(A)<br>250 Hz: 45.8 ± 4.0 dB(A)<br>500 Hz: 52.1 ± 4.0 dB(A)<br>1,000 Hz: 53.0 ± 3.8 dB(A)<br>2,000 Hz: 55.3 ± 2.3 dB(A)<br>4,000 Hz: 54.1 ± 3.3 dB(A)<br>8,000 Hz: 52.3 ± 3.6 dB(A)<br><br>Years of exposure (mean ± SD):<br>Medium exposure group: 6.8 ± 6.9 yrs.<br>Low exposure group: 9.9 ± 7.9 yrs.	
**Melamed et al. 2001**	Cohort	Israel	Cardiovascu-l ar ccupational risk factors determination in Israel follow-up study (median of follow-up 2.6 yr.)	The study was conducted in 21 manufacturing plants, 6 textile, 7 metal works, 3 wood industry, 2 electronic, 2 food production, and 1 printing industry; *information from Melamed et al. (1992)*<br><br>No. of exposed:<br>> 80 dB(A): n=205<br><br>Age (mean ± SD):<br>44.0 ± 10.4 yrs.; range 22 to 62 yrs.	Type of industry: see left<br><br>No. of unexposed:<br>Low exposure to noise < 80 dB(A): n=583<br><br>Age: see left<br><br>Sex: see left<br><br>Response: see left	Hypertension:<br>SBP≥140 mmHg and/or DBP≥90 mmHg and/or the use of antihypertensive medication

				No information according the age distribution in relation to noise exposure<br><br>Sex:<br>451 male; 356 female<br>*No information on sex distribution in relation to noise exposure*<br><br>Response:<br>Over 60 % (Melamed et al. 1992)<br><br>Loss to follow up:<br>86.6 % (Green & Harari 1995, Melamed et al. 2001)<br><br><u>Noise assessment</u><br>Area sampling of noise at each work station measured with sound level meter 150 cm above floor, twice a day in winter and summer. 5 to 10 readings taken during sampling period of 0.5 h. Geometric mean exposure of four samplings.<br><br><u>Noise exposure</u><br>High exposure to noise (n=205): > 80 dB(A)	<u>Noise assessment</u><br>see left<br><br><u>Noise exposure</u><br>< 80 dB(A)	

				Years of exposure:<br>Mean employment: 9.97 yrs.; range 0-36 yrs.<br><br>HPD: not reported (although HPD use was mentioned as possible confounding factor)		
**Parames-warappa and Narayana 2015**	Cross-sectional	India	12/2013-04/2014	Steel plant (Blast furnace, steel melting, rolling mill, sinter plant, machine shop, power plant)<br><br>No. of exposed: 307<br><br>Age:<br>18-20 2 (0.55%)<br>21-30 105 (29.0%)<br>31-40 140 (36.67%)<br>41-50 82 (22.65%)<br>51-60 33 (9.11%)<br>*no information on the age distribution of exposed and unexposed workers*<br><br>Sex:<br>no information	Steel plant (administration division)<br><br>No. of unexposed: 55<br><br>Age: see left<br><br>Sex: see left<br><br>Response: see left<br><br>Noise assessment<br>see left<br><br>Noise exposure (mean (range)):<br>administration: 49 dB(A) (46-52 dB(A))<br>*SD not reported*	Hypertension:<br>SBP>130 mmHg and DBP>80 mmHg

				Response:<br>78.01 %<br>*response in exposed and unexposed workers not reported*<br><br>Noise assessment<br>Stationary measurement of noise for a period of 3 minutes. Minimum, maximum and average reading was recorded. No information according the number of noise measurements.<br><br>Noise exposure (mean (range)):<br>Rolling mill section 102 dB(A) (84-120 dB(A))<br>Steel melting section 91 dB(A) (20-102 dB(A))<br>Blast furnaces 95 dB(A) (82-108 dB(A))<br>Sinter plants 99 dB(A) (84-115 dB(A))<br>Power plant 90 dB(A) (77-103 dB(A))<br>Machine shop 91 dB(A) (86-97 dB(A))<br>*SD not reported*	Years of exposure: no information	

				Years of exposure:<br>no information<br><br>HPD: not reported		
**Pilawska et al. 1977**	Cross-sectional	Poland	1975	Shipyard workers (Hull department with slipway, prefabrication, paint shop, machinery and equipment departments, in which mainly pneumatic devices such as pneumatic hammers and friction saws, welding equipment, sand and shot blasting machines, conveyor systems for compressed air, oxygen, gases, ship engines)<br><br>No. of exposed:<br>high noise exposure: n=1826<br><br>Age:<br>*Age distribution is reported as being similar in workers with high and low noise exposure*<br><br>Sex:<br>*Not reported, presumably predominantly male.*	Shipyard workers working far from loud areas<br><br>No. of unexposed:<br>low noise exposure: n=5825<br><br>Age: see left<br><br>Sex: see left<br><br>Response: see left<br><br><u>Noise assessment:</u><br>see left<br><br><u>Noise exposure</u><br>Daytime: Lm 61-65 dB(A)<br>Nighttime: Lm 48-57 dB(A)<br><br>Years of exposure: see left	<u>Hypertension:</u><br>Physician-diagnosed arterial hypertension during the medical screening. Hypertension was not defined by the authors.

				Response:<br>100 %<br><br><u>Noise assessment</u><br>Noise measured with stationary measurements at 200 workplaces for at least 10 minutes during the day and night shift.<br><br><u>Noise exposure</u>:<br>Daytime: Lm 80-84 dB(A)<br>Nighttime: Lm 71-83 dB(A)<br><br>Years of exposure:<br>*The duration of exposure reported to be similar in workers with high and low noise exposure*<br><br>HPD: Not reported		

**Shaykhlisla-mova et al. 2018**	Cross-sectional	Russia	No information	Extraction of minerals (crude oil and ore minerals), production drilling workers, well-workover operators, oil and gas production operators, equipment maintenance unit operators involved in oil production, sinkers, attachment workers, excavator operators, loading and delivery machine operators.<br><br>No. of exposed:<br>n=801 in crude oil extraction<br>n=680 in ore minerals extraction<br><br>Age: 45.4 ± 0.8 yrs.<br><br>Sex: 100% male<br><br>Response: 100 %<br>*periodic medical examination*<br><br><u>Noise assessment</u><br>No information<br><br><u>Noise exposure</u><br>in oil production workers:<br>Oil drillers: 86-97 dB(A),	Extraction of minerals (crude oil and ore minerals), employees engaged in professional activity without the impact of intensive industrial noise. Information according the job duties of the controls were not given.<br><br>No. of unexposed: n=375,<br>n=133 in crude oil extraction and<br>n=242 in ore minerals extraction<br><br>Age:<br>Comparable to noise exposed workers.<br><br>Sex: 100% male<br><br>Response: 100 %<br>*periodic medical examination*<br><br><u>Noise assessment</u><br>No information<br><br><u>Noise exposure</u><br>< 80dB(A)<br>*(personal communication E.*	<u>Hypertension</u>:<br>SBP/DBP > 140/90 mmHg

				Well-workover operators: 84-85 dB(A)<br>Oil and gas production operators: 83-84 dB(A), Machine drivers: 90-102 dB(A), Noise exposure in ore production workers: walkers: 86-103 dB(A), Fixers: 81-82 dB(A), Loading and delivery machine drivers: 86-94 dB(A), Excavator driver: 81-82 dB(A).<br><br>Years of exposure: 18.9 ± 0.8 yrs., *at least 10 yrs.*<br>*(Basis of the values in Table 1 is 80dB, personal communication E. Shaykhlislamova 2020 May 20)*<br><br>HPD: No information	*Shaykhlislamova, 2020 May 20)*<br><br>Years of exposure: Comparable to noise exposed workers	
**Souto Souza et al. 2001**	Cross-sectional	Brazil	1994	Oil industry (drilling, maintenance [mechanical, electrical, instrumental and welding activities])<br><br>No. of exposed ≥ 85 dB(A): n=472,<br><br>Age:<br>Median 38 yrs., range 27-62 yrs.<br>*no information according the age*	Oil industry, administrative sector<br><br>No. of unexposed: n=303<br><br>Age: see left<br><br>Sex: 100% male	<u>Hypertension</u>: SBP ≥ 140 mm Hg and/or DBP ≥ 90 mmHg, intake of antihypertensive drugs was not evaluated.<br>*Antihypertensive use was not evaluated*

				distribution in exposed and not exposed workers.<br><br>Sex: 100% male<br><br>Response: 68.3%<br>*no information according the response in exposed and non-exposed workers*<br><br><u>Noise assessment</u><br>Noise exposure measurement: No information<br><br><u>Noise exposure</u><br>range of exposure: 86-95 dB(A)<br><br>Years of exposure: > 10 yrs.<br><br>HPD: No information	Response: see left<br><br><u>Noise assessment</u><br>For workers of the administrative sector, who were not exposed to high levels of sound pressure, no dosimetry measures were performed.<br><br><u>Noise exposure</u><br>Not reported, presumably <80 dB (administrative sector)<br><br>Years of exposure: No information	*because the information was not systematically available in the medical records.*
**Stokholm et al. 2013**	Cohort	Denmark	2001-2007, follow-up 2001-2007 ("population was followed from first year of	Workers in 625 companies in 10 trades with the highest levels of compensation claims for occupational hearing loss (manufacture of food, wood products, non-metallic mineral products, basic metals, fabricated	Employees of 100 companies in the financial services<br><br>No. of unexposed: 41,503<br><br>Age:<br>males:	<u>Hypertension</u>: Prescription of antihypertensive medication according the Danish National Prescription

			employ-ment or 1 January 2001 until becoming cases, being censored […] or end of follow-up at 31 December 2007)	metal, machinery, motor vehicles and furniture and construction industry)<br><br>No. of exposed: 103,687<br><br>Age:<br>males:<br><25 yrs.: 13 %<br>25-34 yrs.: 29 %<br>35-44 yrs.: 27 %<br>45-54 yrs.: 18 %<br>55-64 yrs.: 11 %<br>≥65 yrs.: 2 %<br><br>females<br><25 yrs.: 17 %<br>25-34 yrs.: 29 %<br>35-44 yrs.: 28 %<br>45-54 yrs.: 17 %<br>55-64 yrs.: 8 %<br>≥65 yrs.: 1 %<br><br>Sex: 84.8 % male; 15.2% female<br>Response: 100 %<br>*due to record linkage*	<25 yrs.: 8 %<br>25-34 yrs.: 27 %<br>35-44 yrs.: 28 %<br>45-54 yrs.: 22 %<br>55-64 yrs.: 14 %<br>≥65 yrs.: 1 %<br><br>females:<br><25 yrs.: 9 %<br>25-34 yrs.: 26 %<br>35-44 yrs.: 27 %<br>45-54 yrs.: 24 %<br>55-64 yrs.: 13 %<br>≥65 yrs.: 1 %<br><br>Sex:<br>49.3 % male; 50.7 % female<br><br>Response: 100 %<br>*because of record linkage*<br><br><u>Noise assessment</u><br>Full shift noise exposure was measured in 61 employees.<br><br><u>Noise exposure</u><br>< 70 dB(A)	Registry or hospital diagnosis of hypertension according the Danish National Patient Registry. 88 % of hypertension cases were identified by the prescription registry and 12 % by the patient registry.

				Noise assessment<br>Full shift noise exposure was measured in 2001 for 649 workers of 80 randomly selected companies representing all above mentioned trades. In 2009-2010 the noise measurements were repeated for 589 workers in 132 companies. Analysis of time trend showed a 0.1 dB(A) decline annually during the 8-year period from 2001-2002 to 2009-2010.<br><br>Noise exposure<br>>80 dB(A)<br><br>Years of exposure: no information<br><br>HPD: not reported	Years of exposure: no information	
**Talijancic and Mustac 1989**	Cross-sectional	Yugoslavia	No information	Jute weaving mill and a fish processing plant<br><br>No. of exposed:<br>Jute weaving mill: n=90<br>Fish processing plant: n= 90<br><br>Age (range):	Electronic industry<br><br>No. of unexposed: 90<br><br>Age (range):<br>20-55 yrs.<br><br>Sex:	Hypertension:<br>SBP≥160 mmHg and/or DBP≥95 mmHg

				Jute weaving mill: 20-55 yrs.<br>Fish processing plant: 20-55 yrs.<br><br>Sex:<br>Jute weaving mill: 18.9 % male; 81.1 % female<br>Fish processing plant: 100% female<br><br>Response:<br>Jute weaving mill: 100 %<br>Fish processing plant: 100%<br><br><u>Noise assessment</u><br>Measurement methods not reported<br><br><u>Noise exposure</u><br>Jute weaving mill: 90-102 dB*<br>Fish processing plant: 60-90 dB<br>**value reported in abstract, contradicts the text (90-120dB)*<br><br>Years of exposure: at least 5 yrs.<br><br>HPD: Not reported	52.2 % male; 47.8 % female<br><br>Response: 100 %<br><br><u>Noise assessment</u><br>See left<br><br><u>Noise exposure</u><br>≤ 50 dB<br><br>HPD: No information	
**Zhao et al. 1991**	Cross-sectional	Peoples republic of China	1985	Workers in a textile mill<br><br>No. of exposed:	Type of industry: see left<br><br>No. of unexposed:	<u>Hypertension</u>:<br>SBP≥160 mmHg and/or DBP≥95

				104 dB(A): n=164<br>96 dB(A): n=294<br>86-90 dB(A): n=428<br><br>Age (mean ± SD):<br>104 dB(A): 38.51 ± 8.07 yrs.<br>96 dB(A): 37.2 ± 8.64 yrs.<br>86-90 dB(A): 33.93 ± 7.99<br><br>Sex: 100 % female<br><br>Response: mean 75 %<br><br><u>Noise exposure:</u><br>80 noise measurements were taken in different places within the six workshops. According to the factory safety officer`s noise surveys (made every other year), the sound pressure levels were essentially stable since the start of production in 1954. In the groups for which only one SPL is given, the noise levels at different locations did not vary from this value more than 2dB(A) and the TWA exposure of all workers was very close to the value given..	75-80 dB(A): n=215<br><br>Age (mean ± SD):<br>33.90 ± 8.20 yrs.<br><br>Sex: 100 % female<br><br>Response: 89%<br><br><u>Noise exposure</u>:<br>Measurement information see left<br><br>Years of exposure (mean ± SD):<br>14.59 ± 9.34 yrs.	mmHg and/or the use of antihypertensive medication

				Years of exposure (mean ± SD):<br>104 dB(A): 19.76 ± 9.59 yrs.<br>96 dB(A): 18.23 ± 9.87 yrs.<br>86-90 dB(A): 14.18 ± 9.34 yrs.<br><br>HPD: not reported		

Abbreviations: h = hour(s), HPD = Hearing Protection Devices, Hz = Hertz, n = sample size, NA = not applicable, SD = standard deviation, TLV = Threshold Limit Value, , TWA = Time Weighted Average, yrs. = year(s), No. = number, Lm = average noise level

[1]Equivalent exposure (E_m) and hazard index (HI) are synonyms for the evaluation of solvent mixtures by calculating the equation E_m/ HI=$C_1/L_1 + C_2/L_2 \ldots C_n/L_n$, where C is the measured solvent concentration and L is the TLV. Values of E_m/HI above 1 can be interpreted as a solvent mixture above the TLV and values of E_m/HI under 1 as a solvent mixture under the TLV

Table S6. Results shown in included cohort and cross-sectional studies

Study	Prevalence or incidence of arterial hypertension (n/N (%))			Risk estimate			Remarks
	Effect estimate	Exposure group	Control group	Effect estimate	Effect value (95% CI)	Adjusted for	
**Attarchi et al. 2013**	Prevalence	Noise exposure > TLV, no solvents (Group 3): 27/139 (19.4)<br><br>Noise exposure < TLV, solvent exposure > TLV (Group 2): 11/101 (10.9)<br><br>Combined noise exposure > TLV and solvent exposure > TLV (Group 4): 27/107 25.2)	4/124 (3.2)	OR	Control group<br>1.0 (Ref.)<br><br>**Noise exposure > TLV, no solvents (Group 3):**<br>unadjusted:<br>7,23 (2,45-21,32)<br>adjusted:9.43 (2.81-23.46)<br>**corrected adjusted PR 7.41 (2.65-13.60)**<br><br>Noise exposure < TLV, solvent exposure > TLV (Group 2):<br>4.38 (1.27-10.53)<br><br>Combined noise exposure > TLV and solvent exposure > TLV (Group 4):<br>14.22 (3.21-40.84)	Age, work duration, BMI, smoking, dietary salt, regular exercise, shift work, nature of job and family history of hypertension	

**Brahem et al. 2018**	Prevalence	26*/120 (21.7)	7*/120 (5.8)	OR	Crude:<br>4.038 (1.372-11.887)<br><br>**Adjusted:**<br>**4.075 (1.389-11.953)**<br><br>**<u>corrected adjusted PR 3.46 (1.36-7.29)</u>**	Age, BMI, diabetes, family history of hypertension, socio-economic status, smoking, sporting activity and salt intake	*(n/N self-calculated using the percentages given)
**Chang et al. 2009**	Prevalence	Noise exposure only (Group 2) : 4/9 (44.4)<br><br>Solvent exposure and lower noise exposure (Group 3) : 7/15 (46.7)<br><br>Combined noise and solvent exposure (Group 1): 10/18 (55.6)	2/17 (11.8)	OR	**Noise exposure only (Group 2):**<br>**9.1 (1.0-81.1)**<br><br>Solvent exposure and lower noise (Group 3): 7.9 (0.9-66.3)<br><br>Combined noise and solvent exposure (Group 1):	Age, sex, BMI, smo-king, alcohol, exercise and family history of hypertension	

					13.5 (1.5-117.8)<br><br>**corrected adjusted PR 4.66 (1.00-7.78)**		
**Chang et al. 2012**	Prevalence	17/68 (25.0)	Reference Group 1: 14/68 (20.6)<br><br>Reference Group 2 (office workers): 7/52 (13.5)	OR	**Noise exposed vs. reference Group 1:**<br>Crude<br>1.29 (0.58-2.87)<br><br>**Adjusted**<br>**1.37 (0.56-3.36)**<br><br>**corrected adjusted PR 1.27 (0.62-2.26)**<br><br>Noise exposed vs. reference Group 2 :<br>Crude<br>2.14 (0.82-5.64)<br><br>Adjusted<br>1.11 (0.17-7.08)*<br><br>Continuous personal noise exposure:<br>1.01 (0.95-1.07), per 1 dB(A) *(personal*	Age, sex, BMI, smoking cigarettes, alcohol drinking, exercise, family history of hyperten-sion, triglyceride and cholesterol level, educational level, working activity and regular exercise	The adjustment for "working activity" (the amount of physical exertion required by a job) was highly associated with noise exposure: 91.2 % of workers exposed to noise (91.2%) and 7.7 % office workers (reference Group 2) had high activity jobs.<br>The adjustment for working activity may be

					communication, Ta-Yuan Chang)<br><br>*An increase of noise exposure of 30 dB(A) is associated with an OR of 1.35*<br><br><br>Duration of exposure<br>There was no clear relationship between duration of exposure in the noise exposed group and hypertension. The risk was highest after 2-4 year of exposure: (4.43 (1.21-16.15) and decreasing to 1 in the group with ≥6 yr. of exposure.<br><br>Duration as continuous variable: 1.02 (0.87-1.20) per 1 yr. of noise exposure		causing over-adjustmen t.

					(personal communication, Ta-Yuan Chang)<br><br>OR (95% CI) with noise frequency (adjusted):<br>Noise level ≥ 70 dB(A)* at 2,000 Hz1.92 (0.76-4.82)<br><br>Noise level ≥ 70 dB(A) at 4,000 Hz<br>2.05 (0.82-5.12)<br><br>Noise level ≥ 70 dB(A)* at 8,000 Hz<br>2.34 (0.89-6.16)<br><br>**70 dB(A) is the median of the noise exposure in production-line workers in the frequencies between 2,000-8,000 Hz*		

**Chang et al. 2013**	Incidence	High-exposure:<br>38/152 (25.0)<br><br>Intermediate-exposure:<br>59/221 (26.7)	44/205 (21.5)	RR	High-exposure:<br>crude<br>1.38 (0.89-2.13)<br>Model 1<br>1.39 (0.90-2.15)<br><br>Model 2<br>1.96 (1.18-3.27)<br><br>**Model 3**<br>**1.93 (1.15-3.22)**<br><br>Intermediate-exposure:<br>crude<br>1.28 (0.87-1.89)<br><br>Model 1<br>1.28 (0.87-1.90)<br><br>Model 2<br>1.81 (1.14-2.89)<br><br>**Model 3**<br>**1.75 (1.09-2.81)**	Model 1: age at baseline<br><br>Model 2: age at baseline, BMI, employment duration<br><br>Model 3: age at baseline, BMI, employment duration, educational level, cigarette use, alcohol intake, regular exercise	*Cox regression models*<br><br>Mentioned Effect values refer to the outcome "total hypertension"; the authors additionally calculated RR`s for the two subgroups "diagnosed hypertension" and "measured hypertension" Exposure estimate corrected for use of hearing protective devices
**Chen et al. 2005**	Prevalence	(dB(A)) x yrs.<br>85 -<90 dB(A) x yrs.<br>2/47 (4.3)	70 dB(A) x yrs.<br>5/100 (5)	OR	1.047 (1.003-1-092) per dB(A) x yrs.	Age, BMI, hypertension in the family,	*The unit for CNE was given as dB(A) x Years*

		90 -<90 dB(A) x yrs. 22/286 (7.7)<br><br>95 dB(A) x yrs.<br>76/592 (12.8)<br><br>100 dB(A) x yrs.<br>32/161 (19.9)<br><br>105 dB(A) x yrs.<br>9/19 (47.4)<br><br>Total: 146/1205 (12.1)			Unadjusted PR*<br><br>70 dB(A) x yrs.<br>1.0 (Ref.)<br><br>85-<90 dB(A) x yrs.<br>0.85 (0.17 - 4.23)<br><br>90 -<95 dB(A)x yrs.<br>1.54 (0.60 - 3.95)<br><br>95 -<100 dB(A) x yrs.<br>2.57 (1.07 - 6.19)<br><br>**100 -<105 dB(A) x yrs.**<br>**3.98 (1.60 - 9.86)**<br><br>≥105 dB(A) x yrs.<br>9.47 (3.57 - 25.17)<br>*The highest category was not used because of the small number of cases and the extremely high noise level.*	alcohol intake, and ingestion of salted fish	*and sometimes dB(A).*<br><br>**self-calculated*

**De Souza et al. 2015**	Prevalence	**75-85 dB(A)**<br>223/871 (25.60)<br><br>**≥85 dB(A)**<br>131/470 (27.87)	**≤75 dB(A)**<br>76/388 (19.59)	OR	Crude<br>≤75 dB(A)<br>1 (Ref.)<br><br>75-85 dB(A)<br>1.41 (1.05-1.89)<br><br>≥85 dB(A)<br>1.58 (1.15-2.19)<br><br><u>Adjusted</u><br>≤75 dB(A)<br>1 (Ref.)<br><br>75-85dB(A)<br>1.56 (1.13-2.17)<br><br>**≥85 dB(A)**<br>1.58 (1.10-2.26)<br><br><u>**corrected adjusted PR**</u><br><u>**1.42 (1.08-1.81)**</u>	Age, sex, BMI	The noise classes of control group in table 1 (≤75 dB(A) and low exposed workers (75-85 dB(A) are overlapping and including both 75 dB(A). The same is true for the low and high exposed workers. Both classes include 85 dB(A).<br><br>Education and socioeconomic condition (based on availability of running water, refuse collection,

							sewage system and electricity) were not statistically significantly associated with the prevalence of hypertension.
**Fogari et al. 1994**	Prevalence	Total group:<br>87/733 (11.9)*<br><br>Matched group:<br>39/242 (16.1)	Total group:<br>606/8078 (7.5)<br><br>Matched group:<br>22/242 (9.1)	PR#	Total group:<br>1.59 (1.28-1.95)<br><br>Matched group:<br>1.77 (1.08-2.90)<br><br>*These results were not included in the meta-analysis, because same population was described in Fogari et al. 1995 publication.*	Total groups: no adjustment for age and sex<br><br>Matched groups: matched for age (±1 yrs.), duration of exposure (±1 yrs. of employment at the site) and BMI (± 0.5 kg/m²), female	*(n/N self-calculated using the percentages given)<br><br>#self-calculated<br><br>*The authors call their matched analysis a "case versus control analysis", however they matched workers with higher and lower noise exposure in the sense of a*

						workers and workers exposed to a noise level in the range 80-85 dB were excluded.	*cross-sectional study and did not compare hypertensive cases with non-hypertensive controls as a case-control study.*
**Fogari et al. 1995**	Prevalence	*86/733 (11.8)	*604/8078 (7.5) in both control groups combined	PR*	**1.57 (1.27-1.94)**	No adjustment for age and sex	*self-calculated
**Fokin et al. 2018**	Incidence	4/21 (19.0)	10/28 (35.7)	OR, RR, PR*	OR 0.42 (0.11-1.61)<br>RR 0.58 *(CI not given)*<br><br>**PR* 0.53 (0.19-1.47)**	No adjustment for age and sex	*self-calculated

**Giordano et al. 2001**	Prevalence	Age 20-35 yrs.<br>0/33 (0.0)<br><br>Age 35-45 yrs.<br>8/35 (22.9)<br><br>Age 45-60 yrs.<br>8/32 (25.0)	Age 20-35 yrs.<br>0/29 (0.0)<br><br>Age 35-45 yrs.<br>5/36 (13.9)<br><br>Age 45-60 yrs.<br>3/35 (8.6)	PR*	*PR age-adjusted<br>**2.12 (0.91-4.95)**	No adjustment for age and sex<br><br>(sex stratified)	*self-calculated (age-adjusted)
**Ha and Kim, 1991**	Prevalence	Hypertension:<br>SBP/DBP<br>≥ 160/100 mmHg<br>116/1034 (11.2)<br><br>Borderline<br>≥150 to <160 /<br>≥95 to <100 mmHg<br>44/1039 (4.2)	Hypertension:<br>SBP/DBP<br>≥ 160/100 mmHg<br>37/390 (9.5)<br><br>Borderline<br>≥150 to <160 /<br>≥95 to <100 mmHg<br>19/390 (4.9)	PR*	*PR unadjusted<br>1.08 (0.79 - 1.46)<br><br>*PR age-adjusted<br>**0.99 (0.73 – 1.35)**	Age<br><br>*(linear regression with blood pressure values adjusted for further factors)*	*self-calculated
**Hwang et al. 2012**	Incidence rate	50-64 dB(A):<br>3-15 yrs.: 41/162<br>(274.85/10,000 PY)<br><br>>15 yrs.: 46/162<br>(139.77/10,000 PY)<br><br>65-80 dB(A):	40-49 dB(A):<br>48/211<br>(147.35/10,000 PY)	IRR	**Unadjusted IRR**<br>Low (50-64 dB(A))<br>3-15 yrs.<br>1.85 (1.22-2.82)<br><br>>15 yrs.<br>0.95 (0.63-1.42)	Adjusted for age, sex, BMI, low-density lipoprotein, high-density lipoprotein, triglyceride, daily salt	*Additive interaction between AGT gene polymorphisms and noise exposure also examined with*

		3-15 yrs.: 35/90 (372.65/10,000 PY)<br><br>>15 yrs.: 17/88 (94.62/10,000 PY)<br><br>>80 dB(A):<br>3-15 yrs.: 9/27 (344.56/10,000 PY)<br><br>>15 yrs.: 9/54 (86.70/10,000 PY)			Medium (65-80 dB(A))<br>3-15 yrs.<br>2.53 (1.64-3.91)<br><br>>15 yrs.<br>0.64 (0.37-1.12)<br><br>High (81-102 dB(A))<br>3-15 yrs.<br>2.34 (1.15-4.77)<br><br>>15 yrs.<br>0.59 (0.29-1.20)<br>---------------------------<br>Adjusted IRRs<br>Low (50-64 dB(A))<br>3-15 yrs.<br>1.71 (1.11-2.63)<br><br>>15 yrs.<br>0.83 (0.54-1.27)<br><br>Medium (65-80 dB(A))<br>3-15 yrs.<br>2.32 (1.38-3.90)<br><br>>15 yrs.	intake, HPD used, and alcohol consumption	*Rothmann Synergy Index (S = 1.05; 95% CI 0.92-1.19)*

					0.51 (0.28-0.95)<br><br>**High (81-102 dB(A))**<br>**3-15 yrs.**<br>**2.53 (1.14-5.65)**<br><br>**>15 yrs.**<br>**0.56 (0.26-1.23)**		
**Jegaden et al. 1986**	Prevalence	31/164(18.9)	34/291 (11.68)	PR	Unadjusted<br>1.62 (1.03-2.53)<br><br>Adjusted respectively for:<br>**adipositas**<br>**1.89 (1.06-3.35)**<br>alcoholism<br>1.82 (1.02-3.18)<br>genetic predisposition<br>1.81 (1.00-3.30)	Adipositas, alcoholism, or genetic predisposi-tio n	

**Liu et al. 2016**	Incidence	≥80 dB(A)<br>90/312 (28.8)	75-79 dB(A)<br>42/203 (20.7)<br><br>< 75 dB(A)<br>116/487 (23.8)	RR	< 75 dB(A)<br>1.00 (Reference)<br><br>Model 1:<br>75-79 dB(A)<br>1.00 (0.70-1.43)<br><br>≥80 dB(A)<br>1.43 (1.06-1.93)<br><br>Model 2:<br>75-79 dB(A)<br>0.98 (0.68-1.42)<br><br>≥80 dB(A)<br>1.33 (1.00-1.77)<br><br>Model 3:<br>75-79 dB(A)<br>0.98 (0.68-1.42)<br><br>**≥80 dB(A)**<br>**1.38 (1.02-1.85)**	Model1:<br>Age and sex<br><br>Model2:<br>Age, sex, triglyceride level, HPD use (yes vs. no)<br><br>Model 3:<br>Age, sex, triglyceride level HPD use(yes vs. no), body mass index, smoking, alcohol consumption, regular exercise, family history of hypertension	*Significant correlation between frequencies and the prevalence of hypertension were observed at frequencies of 250, 1000, 2000, 4000 and 8000Hz*:<br><br>250Hz:<br>Model 1<br>1.16 (0.93-1.44)<br>Model 2<br>1.26 (1.01-1.54)<br>Model 3<br>1.29 (1.02-1.64)<br><br>1kHz:<br>Model 1<br>1.13 (0.91-1.41)<br>Model 2<br>1.23 (0.98-1.55)<br>Model 3<br>1.25 (0.99-1.59)

							2kHz:<br>Model 1<br>1.07 (0.85-1.33)<br>Model 2<br>1.15 (0.92-1.46)<br>Model 3<br>1.17 (0.92-1.50)<br><br>4kHz:<br>Model 1<br>1.19 (0.92-1.53)<br>Model 2<br>1.29 (0.99-1.69)<br>Model 3<br>1.34 (1.01-1.77)<br><br>8kHz:<br>Model 1<br>1.19 (0.95-1.50)<br>Model 2<br>1.28 (1.01-1.61)<br>Model 3<br>1.32 (1.03-1.69)
**Melamed et al. 2001**	Prevalence	High Noise Exposure<br>Low Job Complexity:<br>26/120 (21.7)	Low Noise Exposure<br>Low Job Complexity:<br>56/246 (22.8)	PR*	***PR 1.21 (0.87-1.68)**<br><br>Logistic regression	age (years), Sex, BMI, HPD use,	**self-calculated adjusted only for job complexity*

		High Job Complexity:<br>26/85 (30.6)	High Job Complexity:<br>66/337 (19.6)		with Noise (Low/High) and an interaction term with Job complexity OR (95% CI)<br><br>Noise (Low/High)<br>0.22 (0.05-0.82)<br><br>Job complexity (Low/High):<br>0.31 (0.09-1.06)<br><br>Noise x Job complexity:<br>2.66 (1.11-6.35)<br><br>high vs low noise [low job complexity] *OR adj.<br>1.71 (0.19-18.0)<br><br>high vs low noise [high job complexity] *OR adj.<br>4.55 (1.22 - 20.0)	Ambient temperature (ºC), White/blue collar, Family history of hypertension	*1.28 (0.88-1.85)*

**Parameswa-rappa and Narayana 2015**	Prevalence	Rolling Mill section, 102 dB: 27/69 (39.1)<br><br>Blast furnaces & Sinter plants, 95 dB: 42/110 (37.8)<br><br>Steel Melting Section, 93 dB: 26/77 (33.8)<br><br>Power plant, utility, civil, QAD, 90 dB: 14/50 (28.0)<br><br>18-20 yrs.: 0/2 (0)<br>21-30 yrs.: 22/96 (22.9)<br>31-40 yrs.: 59/126 (46.8)<br>41-50 yrs.: 19/60 (31.7)<br>51-60 yrs.: 8/21 (38.1)	Control group, 49 dB: 12/55 (21.81)<br><br>18-20 yrs.: 0/0 (0)<br>21-30 yrs.: 1/9 (11.1)<br>31-40 yrs.: 4/14 (28.6)<br>41-50 yrs.: 4/21 (19.1)<br>51-60 yrs.: 4/11 (36.4)	PR*	Rolling Mill vs. Control Group<br>1.83 (0.64-5.19)<br><br>Blast furnaces vs. Control Group<br>1.78 (0.64-5.00)<br><br>Steel Melting Section vs. Control Group<br>1.58 (0.55-4.51)<br><br>Power Plant vs. Control Group<br>1.31 (0.44-3.91)<br><br>**age-adjusted >90dB vs. 49dB**<br>**PR* 1.52 (0.84-2.74)**	No adjustments	*self-calculated
**Pilawska et al. 1977**	Prevalence	23/1,826 (1.26)	32/5,825 (0.55)	PR*	**2.29 (1.35 – 3.91)**	No adjustments	*self-calculated

**Shaykhlisla-mova et al. 2018**	Prevalence	Production of crude oil:<br>Oil drillers<br>114*/277 (41.2)<br>Well-workover operators<br>54*/137 (39.4)<br>Oil and gas production operators<br>39*/162 (24.1)<br>Machine drivers<br>81*/225 (36.0)<br><br>All crude oil production workers<br>288*/801 (36.0)<br><br>Ore minerals mining workers:<br>Fixers<br>31*/167 (18.6)<br>Walkers<br>39*/161 (24.2)<br>Loading and delivery machine drivers<br>43*/289 (14.9)<br>Excavator driver<br>21*/63 (33.3)	Professional workers in oil production<br>22*/133 (16.5)<br><br>Professional workers in ore minerals mining:<br>24*/242 (9.9)	PR	All noise exposed*<br>**2.32 (1.75-3.08)**<br><br>oil drillers*<br>2.33 (1.11-4.89)<br><br>Well-workover* operators<br>2.23 (1.05-4.75)<br><br>Oil and gas production operators*<br>1.36 (0.63-2.96)<br><br>machine drivers*<br>2.04 (0.97-4.30)<br><br>walkers*<br>2.46 (1.10-5.52)<br><br>fixers*<br>1.89 (0.83-4.30)<br><br>Loading and delivery machine drivers*<br>1.51 (0.67-3.39)		*self calculated

		Total ore minerals mining workers<br>134*/680 (19.7)			excavator drivers*<br>3.39 (1.47-7.82)		
**Souto Souza et al. 2001**	Prevalence	119/472 (25.2)	43/303 (14.2)	OR	1.6 (1.0-2.4)<br><br>**Corrected adjusted PR 1.47 (1.00-2.00)**<br><br>Unadjusted<br>1.8 (1.3-2.4)	Age, obesity, schooling, shift work	
**Stokholm et al. 2013**	Incidence	male workers<br>6,051/87,959 PY (6.9)<br><br>75-79 dB(A) x yrs.<br>717/67,633 PY<br><br>80-84 dB(A) x yrs.<br>1,399/102,333 PY<br><br>85-89 dB(A) x yrs.<br>1,567/132,794 PY<br><br>90-94 dB(A) x yrs.	male workers<br>1,536/20,443 PY (7.5)<br><br>< 75 dB(A) x yrs.<br>350/46,123 PY<br><br>female workers<br>2,205/21,060 PY (10.5<br><br>< 75 dB(A) x yrs.<br>503/37,315 PY	IRR	male workers<br>>80 vs. < 75 dB(A)<br>Crude<br>0.96 (0.91-1.02)<br>Adjusted<br>1.06 (0.98-1.14)<br><br>< 75 dB(A) x yrs.<br>1.00 (Reference)<br><br>75-79 dB(A) x yrs.<br>Crude<br>1.27 (1.12-1.45)	Adjusted for age, socioecono-mic status, calendar year, employment status, and duration of exposure	

		2,444/178,060 PY<br><br>95-99 dB(A) x yrs.<br>1,030/48,278 PY<br><br>≥ 100 dB(A) x yrs.<br>80/1,979 PY<br><br><br>female workers<br>1,603/15,728 PY (10.2)<br><br>75-79 dB(A) x yrs.<br>926/50,123 PY<br><br>80-84 dB(A) x yrs.<br>1,238/55,144 PY<br><br>85-89 dB(A) x yrs.<br>452/22,525 PY<br><br>90-94 dB(A) x yrs.<br>537/21,573 PY<br><br>95-99 dB(A) x yrs.<br>152/4,406 PY			Adjusted<br>1.03 (0.90-1.18)<br><br>80-84 dB(A) x yrs.<br>Crude<br>1.69 (1.50-1.90)<br>Adjusted<br>1.00 (0.88-1.14)<br><br>85-89 dB(A) x yrs.<br>Crude<br>1.45 (1.29-1.63)<br>Adjusted<br>1.04 (0.91-1.18)<br><br>90-94 dB(A) x yrs.<br>Crude<br>1.52 (1.35-1.70)<br>Adjusted<br>1.06 (0.92-1.22)<br><br>95-99 dB(A) x yrs.<br>Crude<br>2.36 (2.09-2.67)<br>Adjusted<br>0.98 (0.84-1.15)		

					≥ 100 dB(A) x yrs.<br>Crude<br>4.66 (3.63-5.97)<br>Adjusted<br>**0.99 (0.75-1.31)**<br><br><br><br>female workers<br>>80 versus < 75 dB(A)<br>Crude<br>1.07 (1.00-1.14)<br>Adjusted<br>1.17 (1.09-1.26)<br><br>< 75 dB(A)-yrs.<br>1.00 (Reference)<br><br>75-79 dB(A) x yrs.<br>Crude<br>1.30 (1.16-1.45)<br>Adjusted<br>1.03 (0.89-1.18)<br><br>80-84 dB(A) x yrs.<br>Crude<br>1.57 (1.41-1.74)<br>Adjusted		

					1.10 (0.96-1.25)<br><br>85-89 dB(A) x yrs.<br>Crude<br>1.44 (1.26-1.63)<br>Adjusted<br>1.12 (0.97-1.28)<br><br>90-94 dB(A) x yrs.<br>Crude<br>1.70 (1.50-1.93)<br>Adjusted<br>1.21 (1.03-1.42)<br><br>95-99 dB(A) x yrs.<br>Crude<br>2.40 (1.99-2.89)<br>Adjusted<br>**1.29 (1.03-1.60)**		
**Talijancic and Mustac 1989**	Prevalence	90-102 dB<br>30/90 (33.3)<br><br>60-90 dB<br>10/90 (11.1)	≤ 50 dB<br>2/90 (2.2)	PR*	PR unadjusted*<br>90-102 dB<br>**15.0 (3.69-60.90)**<br><br>60-90 dB<br>5.00 (1.13-22.18)	No adjustments	*self-calculated

**Zhao et al. 1991**	Prevalence	86-90 dB(A)<br>18/428 (4.2)<br><br>96 dB(A)<br>25/294 (8.5)<br><br>104 dB(A)<br>25/164 (15.2)	75-80 dB(A)<br>11/215 (5.1)	OR	1.031 per 1 dB<br><br>*An increase of noise exposure of 30 dB(A) is associated with an OR of 2.48*<br><br>PR unadjusted*<br>86-90 dB(A)<br>0.76 (0.26-2.17)<br><br>96 dB(A)<br>1.53 (0.55-4.26)<br><br>**104 dB(A)**<br>**2.74 (0.99-7.60)**	age, years worked, use of salt (low, normal, or high), family history of hypertension	

Abbreviations: BMI = Body Mass Index, CI = confidence interval; n = sample size; NR = not reported, OR = odds ratio; RR = relative risk, PR = prevalence ratio yr. = year(s), TLV = Threshold Limit Value, CNE = Cumulative Noise Exposure

a) Mantel-Haenszel Estimates

<u>A collection of the abbreviations used in any of the tables above:</u>

h, hour(s); yrs., year(s); n, sample size; NR, not reported; SD, standard deviation; yr., year(s); TWA, time-weighted average; HPD Hearing Protective Devices; BMI, Body Mass Index; CI, confidence interval; n, sample size; NR, not reported; OR, odds ratio; RR, relative risk; PR, prevalence ratio; IRR, incidence rate ratio; PY, person-years; LDL, low-density lipoprotein; HDL, high-density lipoprotein; AGT, angiotensinogen; ROC, Republic of China; Ref., Reference

Table S7: Risk of bias schema

Major risk of bias domains*	Risk	Criteria	Hints/ notes
**1. Recruitment procedure & follow-up (in cohort studies)**:<br><br>***For cohort studies***<br><br>*HINT: We are looking for selection bias:*<br>- *Was the cohort representative of a defined population? #*<br>- *Was everybody included who should have been included? #*<br>- *If response rate is slightly <50% but does not indicate selection bias, it will be listed as a demerit in extraction table.*<br><br>*PRELIMINARY RULING:*<br>- *If the cohort recruitment is based on a convenient/ self-reported sampling OR if response is <10% or not reported, the study will be excluded from analysis.*	low	☐ Cohort recruitment was acceptable.#<br>☐ Baseline response is acceptable (50% or more) OR is <50% and >30%, but substantial differential selection could be excluded.<br>☐ Loss to follow-up is below 20% in total and not different between the two groups (up to 10% difference).*	
	high	☐ Cohort recruitment was not acceptable.#<br>☐ Response not reported/ not calculable.<br>☐ Total loss to follow-up is larger than acceptable (20% or more)* OR drop out differs between the groups by more than 10%* OR the reasons for drop out considerably differ between exposed and non-exposed groups.*	

Major risk of bias domains*	Risk	Criteria	Hints/ notes
For case-control studies<br><br>*HINT: We are looking for selection bias:*<br>- *Were the cases and control subjects representative of the same defined population ("study base"; geographically and/or temporally)? #*<br>- *Was there an established reliable system for selecting all the cases? #*<br>- *The same exclusion criteria are used for both cases and controls. #*<br>- *Comparison is made between participants and non-participants to establish their similarities or differences. #*<br>- *If response rate is slightly <50% but does not indicate selection bias, it will be listed as a demerit in extraction table.*<br><br>*PRELIMINARY RULING:*<br>- *If the recruitment of the study population is based on a convenient/ self-reported sampling OR if response is <10% or not reported, the study will be excluded from analysis.*	low	☐ Case selection and recruitment was acceptable.#<br>☐ Control subjects' selection and recruitment was acceptable.#<br>☐ Non-response was less than 50% for cases and/or control subjects OR it was >50% and <70%, but substantial differential selection of cases and control subjects could be excluded*	
	high	☐ Case selection and recruitment was not acceptable.#<br>☐ Control subjects' selection and recruitment was not acceptable.#<br>☐ Non-response was >70% for cases or control subjects OR it was >50% and<70%, but substantial differential selection of cases and control subjects could not be excluded.*<br>☐ Response not reported/ not calculable	

Major risk of bias domains*	Risk	Criteria	Hints/ notes
For cross-sectional studies<br>*HINT: We are looking for selection bias:*<br>- *Was the study population representative of a defined population? #*<br>- *Was everybody included who should have been included? #*<br>- *If response rate is slightly <50% but does not indicate selection bias, it will be listed as a demerit in extraction table.*<br>*PRELIMINARY RULING:*<br>- *If the recruitment of the study population is based on a convenient/ self-reported sampling OR if response is <10% or not reported, the study will be excluded from analysis.*	low	☐ Recruitment of the study population was acceptable.#<br>☐ Non-response was less than 50% OR it was >50% and <70%, but substantial differential selection of the study population could be excluded.*	
	high	☐ Recruitment of the study population was not acceptable.#<br>☐ Non-response was >70% OR it was >50% and <70%, but substantial differential selection of the study population could not be excluded.*<br>☐ Response not reported/ not calculable.	
**2. Exposure definition and measurement**	low	☐ Exposure was accurately measured to minimize bias (e.g. average daily noise exposure level/LEX, measurement instrument and measurement period, expert estimates based on measurements).#	
	high	☐ Exposure was not accurately measured.#<br>☐ Different methods were used to measure exposure in different groups/ cases and control subjects (***in case-control studies***).§	
	unclear	☐ Not reported.	

Major risk of bias domains*	Risk	Criteria	Hints/ notes
**3. Outcome "rate of/ risk to develop arterial hypertension". Source and validation**	low	☐ Outcome was accurately/ objectively measured to minimize bias[#]<br>☐ Measurement methods were similar in the different groups.[#]	
	high	☐ Outcome was not accurately or subjectively measured (self-reported).[#]<br>☐ Measurement methods were different in the groups.[#]	
	unclear	☐ Not reported.	
**4. Confounding and effect modification**	low	☐ If risk estimators were calculated, major confounding factors (at least age, sex; maybe also pre-existing hypertension, usage of ear protection, stress levels, amount of physical work, sound quality…) were considered.<br>☐ If only prevalence or incidence was assessed, at least sex and age are described.	
	high	☐ Major confounding factors (age, sex) were not considered.	
	unclear	☐ Not reported.	
**5. Analysis method: methods to reduce research specific bias**	low	☐ Authors used adequate statistical models to reduce bias (e.g., standardization, matching, adjustment in multivariate model, stratification, propensity scoring).[§]	
	high	☐ Authors did not use adequate statistical models to reduce bias.	
	unclear	☐ Not reported.	
**6. Chronology**	low	☐ Incident diseases were included.[#]<br>☐ Temporal relation may be established (exposure precedes the outcome).[#]<br>☐ No hypertension known at baseline OR exclusion of prevalent hypertensive people ***(in cohort and case-control-studies).***	

**Major risk of bias domains***	**Risk**	**Criteria**	**Hints/ notes**
	high	☐ Prevalent hypertensive people were included OR people with prevalent hypertension of baseline were not excluded ***(in cohort studies)***.[#]<br>☐ Temporal relation cannot be established.<br>☐ blood pressure is unknown at baseline.	
	unclear	☐ Not reported.	

**Minor risk of bias domains***	**Risk**	**Criteria**	**Hints/ notes**
**7. Blinding of assessors**	low	☐ Assessors were reported or indicated to be blind for individual exposure-status in cohort and cross-sectional studies and to case status in case-control and cross-sectional studies	
	high	☐ Assessors were reported or indicated <u>not</u> to be blind for individual exposure-status in cohort and cross-sectional studies and to case status in case-control and cross-sectional studies	
	unclear	☐ Not reported.	
**8. Funding**	low	☐ Grant/ non-profit-organizations*<br>☐ Study was clearly not affected by sponsors.*	
	high	☐ Sponsoring organization participated in data analysis.<br>☐ Study was probably affected by sponsors.	
	unclear	☐ Industry, combined industry+grant*, unclear if study was affected by sponsors.<br>☐ Not reported.	
**9. Conflict of interest**	low	☐ Reported not having conflict of interest or clear from report/ communication that study	

**Minor risk of bias domains***	**Risk**	**Criteria**	**Hints/ notes**
		was not affected by author(s) affiliation.*	
	high	☐ Conflict of interest exists (at least one author).*	
	unclear	☐ Not reported.	

**Overall risk of bias assessment**		**Low Risk**	**High Risk**	**Unclear Risk**
Major domains	1. Recruitment procedure & follow-up (in cohort studies)			
	2. Exposure definition and measurement			
	3. Outcome "rate of/ risk to develop arterial hypertension". Source and validation			
	4. Confounding and effect modification			
	5. Analysis method: methods to reduce research specific bias			
	6. Chronology			
Minor domains	7. Blinding of assessors			
	8. Funding			
	9. Conflict of interest			
**General rule for rating:**	**Low risk of bias:** low risk in all major domains<br>**High risk of bias:** if not low risk<br>**Overall assessment:**			

Table S8: Leave-one-out analysis

**Study excluded**	**Pooled ES**	**95% Confidence Interval**
Attarchi 2014	1.64	1.42 -1.89
Brahem 2019	1.69	1.45 -1.97
Chang 2010	1.69	1.45 -1.96
Chang 2012	1.74	1.49 - 2.03
Chang 2013	1.72	1.47 -2.02
Chen 2005	1.69	1.45-1.96
De Souza 2015	1.75	1.49-2.06
Fogari 1995	1.75	1.49-2.06
Fokin 2018	1.75	1.51-2.03
Giordano 2001	1.72	1.47-2.00
Ha and Kim 1991	1.77	1.52-2.06
Hwang 2007	1.74	1.50-2.03
Jegaden 1986	1.72	1.47-2.01
Liu 2016	1.75	1.49-2.05
Melamed 2001	1.76	1.50-2.06
Parameswarappa and Narayana 2015	1.73	1.48-2.02
Pilawska 1977	1.70	1.46-1.98
Shaykhlislamova 2018	1.66	1.40-1.97
Siagian 209	1.71	1.47-1.99
Souto Souza 2001	1.74	1.49-2.04
Stokholm 2013	1.81	1.54-2.13
Talijancic and Mustac 1989	1.67	1.45-1.93
Zhao 1991	1.71	1.47-1.99
